# Silica‐Supported Mononuclear Methyl‐Platinum Complexes Stabilized by Trimethylphosphine—Synthesis, Structure, and Reactions Under Reducing Conditions

**DOI:** 10.1002/chem.71409

**Published:** 2026-07-10

**Authors:** Lea S. Kopietz, Sven Schaefer, Oksana Storcheva, Gabriele Raudaschl‐Sieber, Johannes Bitzer, Wolfgang Kleist, Klaus Köhler

**Affiliations:** ^1^ Department of Chemistry, TUM School of Natural Sciences, and Catalysis Research Center Technical University of Munich Garching Germany; ^2^ Department of Chemistry RPTU Kaiserslautern‐Landau Kaiserslautern Germany

**Keywords:** EXAFS spectroscopy, immobilization, isomerization, P ligand, platinum hydride

## Abstract

The chemistry of single‐site platinum surface complexes with phosphine ligands is rarely understood, in contrast to its well‐known chemistry in solution. This report describes the synthesis of a supported bis(phosphine)methylplatinum(II) complex on silicon dioxide (PtMe(PMe_3_)_2_/SiO_2_) and its reactivity with carbon monoxide, hydrogen, and under inert gas. The parent compound and reaction intermediates are characterized by IR‐, solid‐state NMR, and X‐ray absorption spectroscopy. The sterically non‐demanding monodentate phosphine ligands stabilize the platinum centers, allowing the formation of several isolated *trans* complexes on silica. One of them is a platinum hydride that effectively catalyzes the isomerization of 1‐hexene. The stabilization of the molecular hydride contrasts with the reactivity of known supported platinum methyl complexes, such as PtMe(cod)/SiO_2_, which tend to form supported platinum nanoparticles under the applied mild conditions.

## Graphical abstract

The article describes the synthesis and molecular transformations of a silica‐supported bis(phosphine)methylplatinum(II) complex under reactive (H_2_, CO) and inert gas atmospheres. The focus lies on the stabilizing effect of the sterically non‐demanding trimethylphosphine ligand, which alters the reactivity of the surface sites, compared to other supported platinum methyl complexes, and provides stepwise structural information by solid‐state ^31^P NMR spectra and EXAFS.

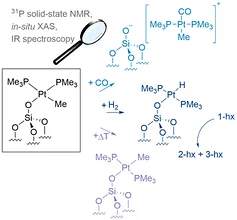

## 1 Introduction

Supported platinum belongs to the most interesting catalysts due to its relevance in a wide range of industrially important reactions, such as hydrogenation, hydrosilylation, CO oxidation, and C─H activations [1, 2, 3, 4, 5, 6, 7, 8, 9]. In principle, platinum‐catalyzed reactions can occur on the surface of a Pt nanoparticle or on a molecular Pt single‐site [3, 10, 11, 12]. Accordingly, interest in atomically dispersed Pt catalysts, often referred to as “single‐atom catalysts”, has grown over the last decade with the aim of effectively using every supported precious metal site [12, 13]. While constructing such sites is often regarded as a bridge between homogeneous and heterogeneous catalysis, the influences of the immediate surroundings on the metal site and its reactivity, commonly known as ligand effects in homogeneous catalysis, are rarely discussed, apart from those coming from the support materials [5, 8, 14, 15, 16].

A challenge for single‐atom catalysts is stabilizing the highly dispersed metal sites [17]. Structural evolution into clusters under reaction or pre‐treatment conditions can impact catalytic activity and the assignment of structure‐activity relationships [13, 18, 19, 20, 21]. To overcome this challenge, different strategies have been applied, e.g., strong‐metal‐support interactions, overcoating, atom‐trapping, or confinement [8, 9, 17, 22, 23, 24]. However, investigating the first steps of cluster formation from the essentially molecular sites as part of their reactivity might elucidate further factors and intermediates during cluster formation, providing more insights and control over the process [1, 15, 25, 26, 27, 28]. Model systems, which consist of supported, atomically dispersed, well‐defined metal centers with structural uniformity, but which can evolve to metal particles or clusters, are a good choice to gain information on this phenomenon [1, 25, 27, 29, 30].

In this context, surface organometallic chemistry (SOMC) provides an approach for constructing model systems for supported catalysts, offering useful insight into reactivity at metal−surface boundary sites and into routes to the formation of supported‐metal clusters and nanoparticles [2]. Within this method, molecular single sites with a well‐defined ligand sphere, as well as particles, can be constructed on oxide surfaces [31]. The latter are characteristic of late transition metals, specifically for noble metals, under reducing conditions [28, 32]. Site‐isolated transition‐metal catalysts—the subject of the present work—are mostly prepared by protonolysis of the transition‐metal center by surface OH groups of oxides, accompanied by elimination of a suitable leaving group [31]. In the case of platinum complexes, this synthetic approach has been successfully applied, using several different precursors and dehydroxylated oxide supports [1, 2, 23, 33, 34, 35, 36, 37, 38, 39, 40, 41, 42, 43]. Starting from these isolated platinum surface complexes, the synthesis of highly dispersed platinum clusters or nanoparticles by SOMC is achieved by controlled reduction or thermal decomposition [2, 33, 34, 36, 41, 42, 44, 45]. Recent developments focused on valuable structural information on the surroundings of the molecular platinum sites by fast‐developing modern techniques of ^195^Pt solid‐state NMR spectroscopy and Extended X‐ray Absorption Fine Structure (EXAFS) [35, 46, 47, 48, 49, 50]. Further, immobilized platinum hydride species have been reported, which catalyze relevant reactions in organic synthesis, but are also proposed as intermediates of cluster formation during reduction [3, 11, 32, 44, 46, 51]. Inspired by that, we anticipated the possibility of capturing structural rearrangements by an appropriate choice of the supported platinum complex for the model system. In that case, insights into the early stages of platinum cluster formation come from surface reactions at well‐defined molecular sites under reducing conditions.

To choose an appropriate model system, we considered different strategies that have been used to stabilize isolated noble metal or platinum surface complexes. These involve, e.g., working under specifically mild reaction conditions, the nature and degree of hydroxylation of the oxide support material, and the careful choice of the precursor complex with particular focus on stabilizing ligands [32, 52, 53, 54, 55]. In view of precursors and stabilizing ligands, bulky, bi‐ or multidentate phosphine ligands were often proposed to optimize stability [2, 11, 23, 52, 54, 55, 56]. While being beneficial for these supported late transition metal sites, soft and acceptor ligands, like phosphines and CO, are also expected to trigger reductive elimination as the first step of aggregation [32]. Steric hindrance, however, also influences the reactivity of a catalytically active complex, particularly at a sterically demanding surface. Phosphines, in general, can serve as spectroscopic handles in ^31^P NMR spectroscopy or as probe molecules, stabilize clusters, and aid in the formation of interesting new phosphide materials [57, 58, 59, 60, 61, 62, 63, 64, 65, 66]. Still, the chemistry of supported phosphine complexes of platinum has not been studied in detail yet, in contrast to supported rhodium entities and homogeneous catalysis [54, 67, 68]. There, platinum complexes with phosphine ligands, including non‐sterically demanding ones, are well‐known. After the first synthetic procedures were reported in the late 1950s [69], numerous mechanistic and kinetic studies provided a detailed picture of this chemistry [70, 71, 72, 73, 74, 75].

With this in mind, it is very interesting to apply [Pt^II^Me_2_(PMe_3_)_2_] in grafting as a model system. It combines a small, sterically non‐demanding representative of a phosphine with methyl‐metal groups, which are reactive in grafting and form methane as a leaving group. The surface site is then stabilized by sterically non‐demanding phosphine ligands, which serve as the basis for surface reactivity studies.

This work focuses on the synthesis, characterization, and reactivity of a highly reactive platinum surface complex on silica, with particular emphasis on the role of the small trimethylphosphine (PMe_3_) ligands. The genesis of the surface complexes starts with grafting of [Pt^II^Me_2_(PMe_3_)_2_]. Reactions of the surface complexes with carbon monoxide and in an inert gas atmosphere were studied under mild conditions and compared with the well‐known chemistry in solution. The decomposition under hydrogen at increasing temperature was investigated, comparing the bis(phosphine)platinum surface complexes with those of chelating ligands, such as 1,2‐bis(dimethylphosphino)ethane (dmpe) and 1,5‐cyclooctadiene (cod). One specific aspect was the formation of platinum hydrides and their role in agglomeration and isomerization catalysis (1‐hexene). Educts and products of the surface reactions were characterized extensively, combining techniques for molecular structures (IR, MAS‐NMR) with those for potential Pt clusters (EXAFS, chemisorption) and desorbed species (on‐line mass spectrometry). The techniques were applied, when possible, during the reactions (in‐situ measurements) or after each reaction step, assuring a multi‐perspective, detailed picture of the ongoing transitions.

## 2 Results and Discussion

### 2.1 Synthesis and Characterization of PtMe(PMe_3_)_2_ on Silicon Dioxide

For the synthesis of the surface complex, the dimethyl platinum precursor was used. It can be obtained by several reported procedures [69, 72, 76]. For the materials discussed here, the original method by Chatt and Shaw was adapted [69]. Originally, PtCl_2_(PMe_3_)_2_ is methylated by methyllithium at room temperature in benzene. As we intended to construct isolated, mononuclear noble metal sites on silica, we preferred keeping the temperature throughout the synthetic steps and during storage of the precursor and grafted material mostly below room temperature to minimize the risk of self‐reduction and unwanted aggregation. The modified precursor synthesis at 0°C in toluene still yielded mostly the *cis*‐isomer of PtMe_2_(PMe_3_)_2_ (details in Supporting Information) [69]. This can be concluded from the ^31^P NMR signal at −24.0 ppm in C_6_D_6_ solution (^1^ *J*_PtP_  = 1785 Hz, Figure S2) [72, 76]. In analogy, the reaction of PtCl_2_(PMe_3_)_2_ with a solution of methyl‐d_3_‐lithium yielded mostly *cis*‐Pt(CD_3_)_2_(PMe_3_)_2_, which has a ^31^P NMR signal at −23.9 ppm in C_6_D_6_ (Figure S2, ^1^H NMR spectra of both compounds in Figure S1). The formation of minor side products could not be avoided in the adapted syntheses of the precursors.

Both precursors were immobilized on silicon dioxide, which has been partially dehydroxylated at 700°C under high vacuum (SiO_2_^700^). This pre‐treatment of the support material leads to isolated silanol groups on the surface, which react with the dimethyl complexes (grafting) to yield site‐isolated platinum‐methyl surface complexes [31]. Formation of methane during the immobilization of PtMe_2_(PMe_3_)_2_ was detected in a study of the grafting reaction on an NMR scale (Figure S5). Grafting on a larger scale was performed in *n*‐pentane for 24 h under cooling by an ice‐water bath (Scheme 1). Washing and drying in vacuo led to the colorless materials PtMe(PMe_3_)_2_/SiO_2_^700^ (**A**) and the d_3_‐analog Pt(CD_3_)(PMe_3_)_2_/SiO_2_^700^ (**A‐d_3_**). The latter material was supposed to give a clear differentiation of the reaction of the Pt‐Me group and the P‐Me groups in mass spectrometry and IR spectroscopy. The amounts of the precursors for grafting were chosen to give supported materials with 1.0–2.5 wt% platinum loading. In this range, not all surface ≡SiOH groups are supposed to react, but site‐isolation of the surface complexes is further assured. Due to that, the reactions of the mononuclear surface sites can be studied after their characterization, and potential aggregation can be traced back to the reductive conditions applied.

**SCHEME 1:**
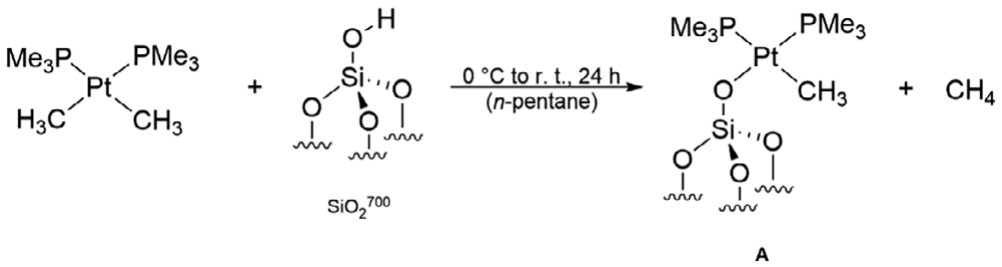
Grafting reaction of dimethylbis(trimethylphosphine)platinum(II) with silica‐700: formation of supported, isolated platinum phosphine complexes on silicon dioxide (only the major species depicted).

An IR spectrum of a self‐supporting disc of **A** shows bands between 3000–2800 cm^−1^, and between 1500–1400 cm^−1^ (Figures 1 and S7, dark blue). The first region is typical for C─H stretching, the second region for deformation vibrations of the organic ligands of the platinum surface complexes [2, 36, 43, 67, 68]. The stretching bands at 2979 and 2918 cm^−1^, and the deformation bands at 1437 and 1423 cm^−1^ correspond to the reported ones for immobilized rhodium trimethylphosphine complexes (Figure S7) [67, 68]. Further, SiO─H stretching of isolated silanol groups on the surface of silica‐700 is observed as the sharp band at 3747 cm^−1^ [31]. The Si─O overtone and combination vibrations are located at 1977, 1868, and 1647 cm^−1^, as expected [2]. An IR spectrum of **A‐d_3_** shows, additionally to C─H stretching and deformation bands of PMe_3_, C─D stretching bands of low intensity (Figures S6 and S10). Supporting harmonic frequency calculations of geometry‐optimized DFT models are in good accordance with the experimental spectra (details in Supporting Information).

**FIGURE 1:**
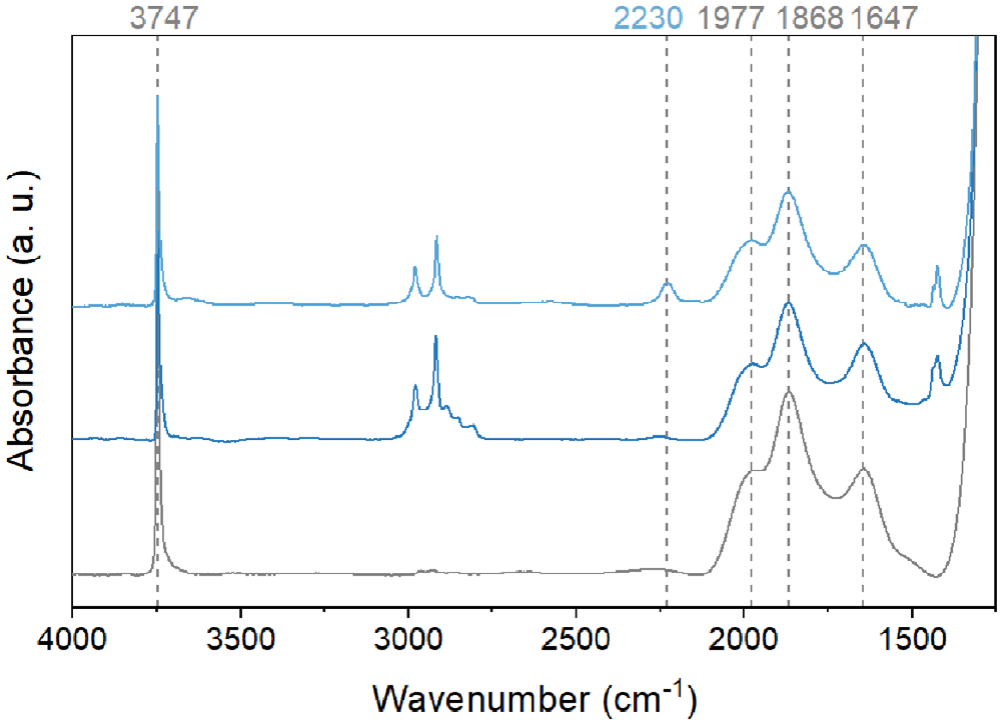
FT‐IR spectra of self‐supporting discs of SiO_2_^700^ (grey), PtMe(PMe_3_)_2_/ SiO_2_^700^ (**A**, dark blue), and PtMe(PMe_3_)_2_/SiO_2_^700^ after a pre‐treatment in 10% hydrogen in helium at 150°C for 30 min (**A‐H**_**2**_ **‐150**, light blue).

The chemical composition and platinum loadings of **A** and **A‐d_3_** were tested by elemental analysis. The results are summarized in Table S9. Expected and found contents lay closely and support the composition of **A**, which is indicated in the formula and in Scheme 1. Coverages of 20%–49% of the isolated SiOH groups with the surface complexes are expected (Table S12). The stoichiometries of the second entry (Pt_1.0_C_8.8_H_25.1_P_2.0_) would also fit to ≡Si‐Me groups, in addition to the surface complex (PtC_7_H_21_P_2_ and ‐CH_3_). The groups form in a grafting mechanism proceeding via breaking up strained siloxane rings of the surface, as proposed by Gioffré *et al.* for dimethyl platinum precursors [43]. Detection of methane during grafting and no clear indication of the ≡Si‐Me groups in the solid‐state ^1^H NMR spectrum (δ = −0.14 ppm [43], Figure S14) render this mechanism unlikely in the present case. Rather, residues from the synthetic procedure (solvent, grease) cause slightly higher molar ratios of carbon and hydrogen with respect to platinum.

The structure of the surface complex is deduced from the solid‐state ^31^P{^1^H} NMR spectrum (Figure 2a). It shows two signal groups: one at −35 ppm and one at −11 ppm with a shoulder at −9 ppm. In general, two signals indicate inequivalent phosphorous atoms, in accordance with a *cis* geometry of the phosphine ligands. In combination with the platinum satellites, the signal at ‐35 ppm and the shoulder at ‐9 ppm are assigned to the *cis* structure. The ^195^Pt‐^31^P coupling constants amount to 4208 and 1628 Hz (see Figure 2 and Table 1). The large coupling constant is diagnostic for a phosphine with a very weak *trans* donor, which is the siloxy ligand [72, 75]. The small coupling constant corresponds to trimethylphosphine with a *trans* standing carbon, which is here the methyl group [72, 75]. The coupling constants are comparable to the molecular *cis* isomers of the monoalkyl bis(phosphine) platinum complexes, detected at low temperatures in solution (Table S15, entries 2 and 4) [72, 75]. The signal at—11 ppm indicates that a second geometry with equivalent phosphine ligands is present on the surface. Platinum satellites can be assigned to the signal, resulting in a ^195^Pt‐^31^P coupling of 2870 Hz. The coupling constant is lower than those of the reported bis(grafted) trimethyphosphine species (Lit.: 3607 and 3692 Hz) [40], or the molecular bis(siloxy) complex Pt(OSiMe_3_)_2_(PMe_3_)_2_ (Lit.: 3400 Hz) [77]. It is comparable to the *trans* form of the solvento complexes (Table S15, entries 3 and 5), which form by *cis‐*to‐*trans* isomerization at low temperature in solution [72, 75]. Consequently, a surface complex in the parent material may have the *cis* or the *trans* orientation of the phosphine ligands. From the signal intensities in the spectrum, the *cis* geometry is preferred (ratio: 2:1, *cis*:*trans*). It retains the structure of the precursor during grafting onto the surface. It exemplifies the stabilizing effect of the surface, compared to polar solutions.

**FIGURE 2:**
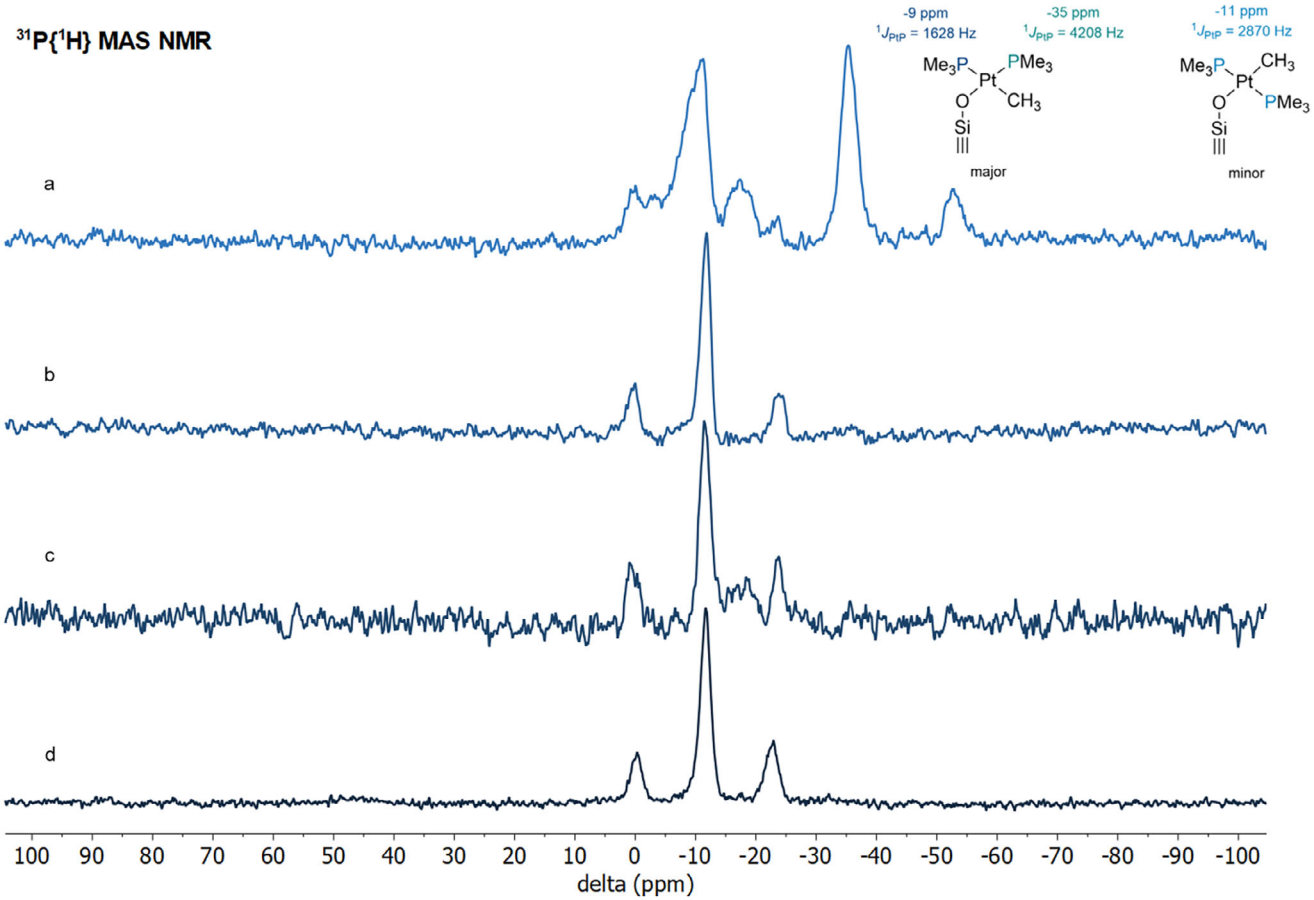
Proton‐decoupled, solid‐state ^31^P NMR spectra (121 MHz) of densely‐packed rotors (4 mm) with magic‐angle spinning (MAS) (a) as‐synthesized PtMe(PMe_3_)_2_/SiO_2_^700^ (**A**, MAS: 15 kHz), (b) PtMe(PMe_3_)_2_/SiO_2_^700^ after a pre‐treatment in helium flow (50 mL min^−1^, 200°C, 5 min), denoted as **A‐He‐200** (MAS: 13 kHz), (c) PtMe(PMe_3_)_2_/SiO_2_^700^ after a pre‐treatment in carbon monoxide (1% in helium, 50 mL min^−1^, r. t., 30 min), denoted as **A‐CO** (MAS: 13 kHz), and (‐d) PtMe(PMe_3_)_2_/SiO_2_^700^ after a pre‐treatment in hydrogen (5 mL min^−1^, mixed with helium 45 mL min^−1^, 150°C, 30 min), denoted as **A‐H**_**2**_ **‐150** (MAS: 12 kHz).

**TABLE 1:** Summary of discussed surface species, pre‐treatments, CO stretching vibrations, and chemical shifts in solid‐state ^31^P NMR spectra with ^195^Pt‐^31^P coupling constants. For all compounds, including molecular complexes from literature, see Table S15.

Entry	Species	Pre‐treatment (gas^a^, temperature (°C)^b^, dwell time (min))	υ_CO_ (cm^−1^)	δ_P_ (ppm)	Coupling constant ^1^ *J*_Pt‐P_ (Hz)
1	≡SiO‐PtMe(PMe_3_)_2_ (**A**)	None			
	(*cis*)			−35, −9	4208, 1628
	(*trans*)			−11	2870
2	**A‐CO**	1% CO, r. t., 30			
	≡SiO^−^ [PtMeCO(PMe_3_)_2_]^+^		2092	−11	2960
	≡SiO ‐Pt(Me)(CO)(PMe_3_)		2071, 2078 (shoulder)	−18 (?)	
3	**A‐He‐200** ≡SiO‐PtMe(PMe_3_)_2_ (*trans*)	Helium, 200, 5		−12	2884
4	**A‐He‐200_CO**		2092		
5^c^	**A‐H_2_‐150**	10% H_2_, 150, 30			
	≡SiO‐Pt(H)(PMe_3_)_2_ (*trans*)			−12	2747
	Pt_x_(PMe_3_)_y_/SiO_2_				
6	**A‐H_2_‐150_CO**				
	≡SiO‐Pt(H)(PMe_3_)_2_ (*trans*)		Decomp.		
	Pt_x_(PMe_3_)_y‐n_(CO)_n_/SiO_2_		2065, 1990, 1780		
7	**A‐H_2_‐350**; Pt_x_P_y_/SiO_2_	10% H_2_, 350, 5			
8	**A‐H_2_‐350_CO**; Pt_x_P_y_/SiO_2_		2040		

^a^ Balance in helium, pre‐treatment in flow, in total 50 mL min^−1^.

^b^ For temperatures different from r. t., the sample was heated up with 5 Kmin^−1^ in the respective gas (mixture), before holding the given dwell time.

^c^ υ_Pt‐H_: 2230 cm^−1^, δ_H_: −22 ppm (Pt‐H, ^1^ *J*_Pt‐H_ = 1167 Hz)

Further structural insights come from X‐ray absorption analysis at the L_3_‐edge of the platinum centers (Figure S11, S12, and Table S13). The whiteline intensity and edge position of **A** are higher than those of the foil, indicating oxidized centers in the grafted sample. In the Fourier‐transformed spectrum, no Pt‐Pt contacts were detected. It proves that the synthesis and immobilization proceed without the formation of platinum aggregates. Fitting of the EXAFS data was performed using the data of the crystal structure of the molecular complex *cis*‐PtMe(OPh)(PMe_3_)_2_ as a starting point (Table S13) [73]. The complex is an appropriate description of the immediate surroundings of the Pt^II^ center (Figure S12). It has two light scatterers (Pt‐C/O) at a distance of 2.08 Å, and two further scatterers at a distance of 2.27 Å (Pt‐P) in its first coordination shell (Table S13). It underlines the molecular character of the supported platinum(II) sites in good agreement with the well‐resolved solid‐state NMR spectra.

### 2.2 Reactivity With Carbon Monoxide: Formation of a Surface Bound [PtMeCO(PMe_3_)_2_]^+^ Complex

After the characterization of the supported, mononuclear platinum sites on silica‐700, their reactivity with carbon monoxide at room temperature is discussed in the following. The in‐situ IR spectra show two intense, sharp bands at 2071 and 2092 cm^−1^ (Figure 3 and Table 1). The shift of their normalized intensities over time is depicted in Figure 3b. Generally, CO stretching vibrations of linearly chemisorbed CO molecules on platinum particles (Pt^0^‐CO) are expected in this region [1, 3, 20, 23, 30, 63, 78]. Particularly, an intensity shift from 2070 to 2090 cm^−1^ has been reported for CO chemisorption on platinum particles, when going from low to high coverages [79]. The bridging CO band at 1850 cm^−1^ with weaker intensity is in line with platinum particles. However, CO bands of comparable molecular platinum(II) carbonyl complexes (e.g., Table S15, entries 7 and 8) and of silica‐supported rhodium carbonyl complexes lie in the same region as Pt^0^‐CO [68, 75, 80]. Since reductive decomposition and evolution of the oxide‐supported surface complexes to clusters is possible with carbon monoxide at room temperature, [52, 81, 82, 83, 84] X‐ray absorption spectroscopy was applied on a pre‐treated sample to test for a change of the oxidation state and the possible presence of platinum clusters. The edge position and whiteline intensity are still different from the platinum foil, indicating oxidized platinum centers in the sample (Figure S23). The Fourier‐transformed EXAFS spectrum shows no Pt‐Pt contacts and no significant changes in the coordination sphere around the platinum scattering center with respect to the parent material (Figure S23). The same is true for pre‐treatments under carbon monoxide at 100°C and 150°C (Figure S24). Hence, the bands in the region of terminal CO vibrations arise from molecular carbonyl phosphine complexes rather than from supported platinum aggregates (Scheme 2).

**FIGURE 3:**
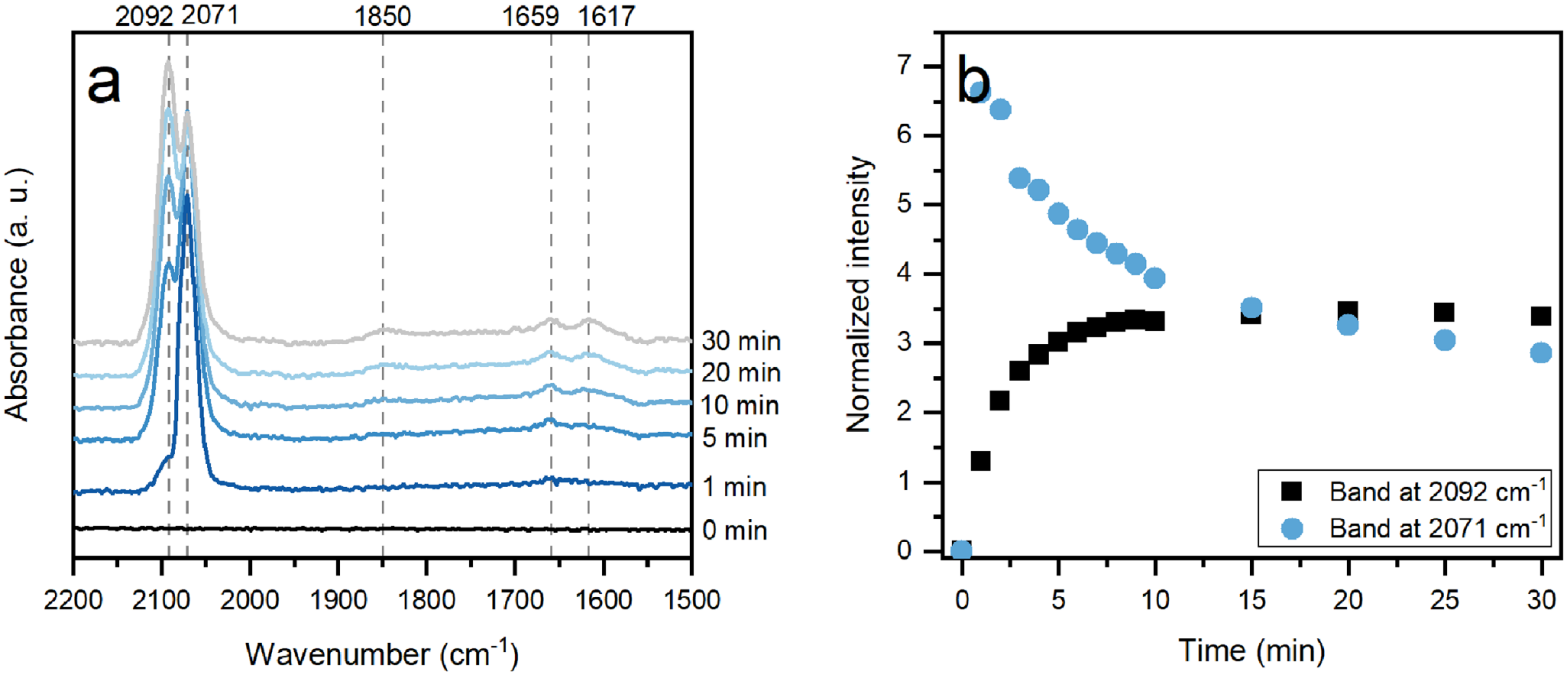
(a) Time‐dependent IR spectra of PtMe(PMe_3_)_2_/SiO_2_^700^ in a flow of carbon monoxide (1% in helium, 20 mL min^−1^) at room temperature in the region of C–O stretching vibrations: a spectrum of CO passing over SiO_2_^700^ has been subtracted, and (b) temporal evolution of the intensities of terminal CO stretching bands, normalized to the band of gas‐phase CO at 2171 cm^−1^. The sample was transferred and measured, avoiding air contact.

**SCHEME 2:**
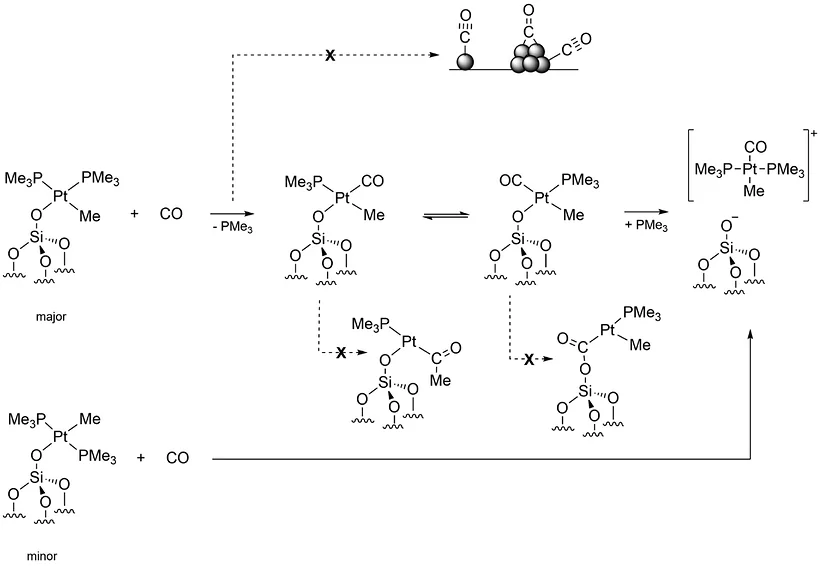
Proposed reaction mechanism of the surface complex **A** with carbon monoxide at room temperature.

Insertion of CO into the Pt‐C bond from the orientation of CO and the methyl ligand *cis* to each other would lead to an acyl species. Its CO stretching band is expected to be around 1635 cm^−1^ [80, 85]. If carbon monoxide inserts into the Pt‐O bond, like in platinum complexes with fluorinated alkoxide ligands [73], or siloxide ligands in moist methanol [86], a band in the same region will arise. For the supported platinum methyl complex, the CO insertion is not clearly observed, even though a weak intensity band at 1617 cm^−1^ forms (Figure 3 and Scheme 2). This reactivity of the platinum methyl surface complex contrasts with the silica‐supported, molecular palladium and rhodium entities, which insert CO into the metal‐carbon bond [52, 67].

Ligand exchange between the phosphine and carbonyl ligand during the presence of CO in the IR cell is indicated by the negative C─H stretching bands in the subtracted IR spectra (Figures S20 and S21). Mass spectrometric analysis of the out‐streaming gas flow during the treatment of **A** in a flow of carbon monoxide shows little desorption of the phosphine (Figure S22), but primarily the phosphine stays on the surface.

In order to identify the structure of the molecular species formed under carbon monoxide, solid‐state NMR spectroscopy was applied to a sample, which has been pre‐treated in a flow of carbon monoxide (1% in helium, 50 mL min^−1^) at room temperature for 30 min (**A‐CO**). The proton‐decoupled, ^31^P spectrum shows a signal at −11 ppm (^1^ *J*_PtP_ = 2960 Hz), in line with a *trans* orientation of the phosphine ligands (Figure 2c). The chemical shift is close to the molecular complex [PtMe(CO)(PMe_3_)_2_]^+^ in solution (δ_P_ = −13.6 ppm; Table S15, entry 7) [75]. It forms from the exchange of acetone‐d_6_ in the solvento complex [PtMe(acetone‐d_6_)(PMe_3_)_2_]^+^ with carbon monoxide at low temperature [75]. The IR band of the carbonyl ligand in the molecular complex is likewise at 2092 cm^−1^ [75]. The formation of similar species, electrostatically attached to the surface, is feasible from the present data. It could form directly by ligand exchange with the surface siloxy ligand from the *trans* isomer, or by ligand exchange and isomerization from the *cis* isomer [87]. The proposed reactions with the structures are summarized in Scheme 2 and Table 1 (entry 2).

### 2.3 Reactivity Under Inert Gas: *Cis*‐to‐*Trans* Isomerization

The influences of *cis*‐*trans* isomerization of bis(phosphine)platinum(II) complexes in solution have been extensively studied [71, 72, 75, 88]. Solvento complexes with methanol and acetone ligands are examples (Table S15, entries 2–5) [72, 75], which assimilate the surroundings of the supported platinum center. The solvento complexes isomerize at low temperatures when the solution is gradually warmed via a T‐shaped transition state after the dissociation of the solvent molecule. Surface organometallic investigations on supported platinum complexes in this direction have not yet been carried out. Due to that, the supported bis(trimethylphosphine)platinum(II) complex was treated under an inert atmosphere with stepwise increasing temperature, mimicking the warming of a polar solution. To define the limit of the supported molecular species, a temperature‐programmed reaction and desorption with mass spectrometric analysis of the gas phase products was carried out. It indicated that compound **A** decomposes above 250°C in an inert gas flow (Figure S25). In‐situ X‐ray absorption spectroscopy was applied to define the influence of gradual reduction and formation of platinum metal particles [36]. It corroborates the stability of **A** up to at least 210°C under inert gas flow, as no reduction or formation of platinum aggregates was detected in the spectra (Figure S26). The findings are comparable to the thermolytic chemistry of other supported, molecular platinum centers, which were likewise stable up to 150°C–200°C under inert conditions [36]. In‐situ IR spectroscopy shows small changes in the region of C─H stretching vibrations below 200°C (Figure S27). It implies a molecular rearrangement. To identify if, in fact, a molecular rearrangement took place, a sample was pre‐treated under helium gas flow (50 mL min^−1^) at 200°C for 5 min (**A‐He‐200**). The obtained ^31^P solid‐state spectrum of the sample shows the signal of the *trans* structure at −12 ppm (Figure 2b and Table 1, entry 3). The coupling constant is 2884 Hz. The spectrum clearly supports the formation of the *trans* isomer under inert gas [72, 75]. The nature of the species was further investigated with CO chemisorption at room temperature as an in‐situ IR experiment (**A‐He‐200_CO**, Table 1). In Figure S16, the spectrum after the CO chemisorption and a helium purge of the IR cell is depicted. The band at 2092 cm^−1^ has formed, while the band at 2071 cm^−1^ has not. It contrasts the spectrum of the parent material after its reaction with CO (dark blue, Figure S16). It implies that the *trans* isomer reacts directly to the surface‐bound carbonyl species, and the *cis* species was primarily responsible for the band at 2071 cm^−1^ (compare Scheme 2).

### 2.4 Reactivity With Hydrogen: Generation of an Immobilized Platinum‐Hydride Species

Classically, supported noble metal sites form highly‐dispersed metal nanoparticles under hydrogen [3, 27, 29, 31, 41, 44, 45, 51, 57]. It was shown in former studies on supported platinum complexes that the ligand could alter the reactivity or the material that forms upon the treatment [2, 3, 44, 45]. In order to identify whether the monodentate phosphine ligand has an impact on the platinum cluster and nanoparticle formation under hydrogen, the reaction of **A** with hydrogen was tested.

First, a temperature‐programmed reaction with mass spectrometric analysis of the out‐stream shows that **A** reacts with hydrogen between 150°C and 200°C under desorption of methane (m/z 15, 16, Figure S29). **A‐d_3_** reacts under the same conditions, but CD_3_H (m/z 19) desorbs instead of CH_4_ (Figure S30). It proves the hydrogenolysis of the Pt‐Me bond, in analogy to the supported complex PtMe(CpMe)/Zn/SiO_2_ [3]. At temperatures above 250°C, the ligand decomposes with dealkylation, as fragments of the trimethylphosphine ligand were detected in the outstream (m/z 61, 76 Figures S29 and S30) with further methane formation (m/z 15, 16) [58].

To identify the impact of the phosphine in the reduction of the platinum centers and the formation of Pt‐Pt contacts, we performed X‐ray absorption spectroscopy of **A** during heating in a flow of hydrogen in nitrogen (Figure 4). The whiteline intensity in the spectra assimilates that of the platinum foil, whereas the edge positions of the grafted sample and the foil differ. Pt‐Pt contacts in the EXAFS spectra are not clearly identified. It proves that the phosphine‐supported platinum sites react differently under hydrogen than in the generation of platinum nanoparticles.

**FIGURE 4:**
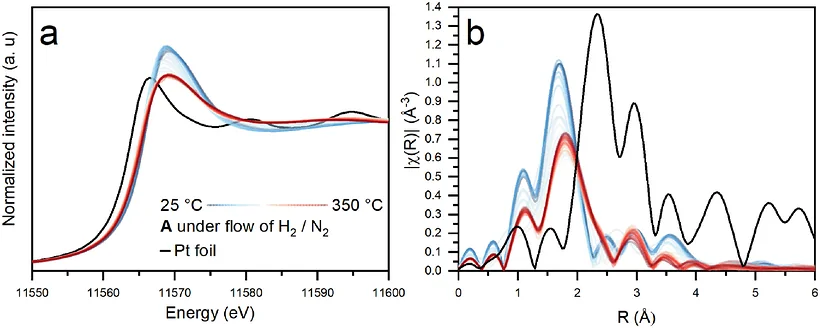
(a) In‐situ X‐ray absorption spectra of **A** filled into a quartz glass capillary under a flow of H_2_ (10% in nitrogen, 20 mL min^−1^) with 5 K min^−1^ heating rate to 350°C (color) in contrast to a spectrum of a platinum foil (black), and (b) Fourier‐transformed spectra thereof.

The changes of the molecular structure were further tested by in‐situ IR spectroscopy under a flow of hydrogen in helium (20 mL min^−1^ in total, Figure S31). The temperature‐dependent spectra between 120°C and 220°C show a band at 2230 cm^−1^. It formed at this position, regardless of whether **A** or **A‐d_3_** was treated under hydrogen (Figure S33). The negative C─H stretching bands indicate the dissociation of the organic ligands, in line with the detection of the organic fragments in mass spectrometry. Hydrogenolysis of the Pt‐Me group is verified by negative C─D stretching bands in the spectra of **A‐d_3_** (Figure S33), in agreement with mass spectrometric analysis under hydrogen. To identify the incorporation of H_2_ from the gas phase, an analogous experiment was carried out under deuterium flow (Figure S32). The band at 2230 cm^−1^ did not form, but surface SiO─H groups were exchanged to SiO─D, which is observed by the band at 2758 cm^−1^ (Figure S32) [1]. The process is observed in the spectra starting at 70°C.

The IR spectroscopic data indicates the formation of a Pt‐H species under hydrogen. The Pt‐H stretching band at 2230 cm^−1^ is close to the reported one of the molecular complex *trans*‐PtH(OCN)(PEt_3_)_2_ (Lit.: 2234 cm^−1^) [89]. An isotope‐shifted Pt‐D band is not clearly observed in the spectra, likely due to the bands of the Si‐O overtone vibrations changing with temperature (Figure S32).

Then, it was tested if **A** can be specifically pre‐treated between 120°C–220°C to form the supported Pt‐H species, or if it is only stable under hydrogen flow [3]. After the pre‐treatment of **A** at 150°C in a flow of hydrogen (5 mL min^−1^, in helium 45 mL min^−1^) for 30 min (**A‐H_2_‐150**), a self‐supporting disc was pressed and transferred to the IR spectrometer under inert conditions. The spectrum (Figure 1, light blue) still shows the Pt‐H stretching band at 2230 cm^−1^, proving the formed species is present independent of the hydrogen atmosphere. It allowed the collection of solid‐state NMR spectra of **A‐H_2_‐150** to identify structural features (^1^H, ^31^P). In the proton‐decoupled ^31^P solid‐state NMR spectrum (Figure 2d), a molecular compound with *trans* orientation of the phosphine ligands is identified by the signal at δ = −12 ppm. The coupling constant (^1^ *J*_PtP_ = 2747 Hz, Table 1) and chemical shift are comparable to the molecular complex [PtH(acetone‐d_6_)(PMe_3_)_2_]^+^ (δ_P_ = −10.5 ppm, ^1^ *J*_PtP_ = 2540 Hz; Table S15, entry 12) [74]. In the solid‐state ^1^H NMR spectrum (Figure S34), the platinum hydride is identified by the signal in high field at δ = −22 ppm (^1^ *J*_PtH_ = 1167 Hz, Table 1). It is equivalent to the ^1^H NMR signals of [PtH(OH)(PMe_3_)_2_] in solution at low temperature (δ = −22.08 ppm, ^1^ *J*_PtH_ = 1136 Hz) [74].

During the pre‐treatment under hydrogen, the color of the material changes from colorless to brownish. The solid‐state ^31^P NMR spectrum (Figure 2d) also shows a broader signal between 40 and 50 ppm. Both observations indicate different species form next to the molecular platinum hydride. EXAFS analysis of **A‐H_2_‐150** confirms the formation of mononuclear, surface‐anchored Pt species (Table S16 and Figure S35). The best fit is obtained with two scattering paths for the PMe_3_ ligands and one Pt‐oxygen contribution, which is consistent with the silica surface coordination (Table S16). Compared to precursor **A**, the data validate the loss of the methyl group. Including an additional Pt‐Pt scattering shell did not improve the quality of the fit and resulted in physically nonsensical values, demonstrating the absence of Pt‐Pt interactions and ruling out the formation of Pt nanoparticles or clusters.

**A‐H_2_‐150** was further tested by CO chemisorption as an in‐situ IR experiment at room temperature to identify the nature of the site. Upon contact with CO, the Pt‐H band vanishes (negative band at 2230 cm^−1^, Figure S16), corresponding to the decomposition of the hydride. A broad band at 1785 cm^−1^ forms, next to lower intensity bands at 2065 and 1990 cm^−1^ (**A‐H_2_‐150_CO**, Table 1, entry 6). The band positions are in line with bridging and terminal CO vibrations of platinum carbonyl phosphine clusters (Table S15, entry 14–16), which contradicts EXAFS fitting results [59, 90]. However, the formation of these clusters could explain the color change of the material. Pre‐treatment at higher temperatures leads to the decomposition of the phosphine‐decorated platinum clusters and the molecular Pt‐H site. A phosphorus‐modified platinum material, supported on silica, results. As an example, **A** was pre‐treated in the mixture of hydrogen and helium at 350°C for 5 min (**A‐H_2_‐350**), and a self‐supporting disc of the material was pressed under inert conditions. The IR spectrum of **A‐H_2_‐350** after CO chemisorption at room temperature gives a band at 2040 cm^−1^ (Figure S16, **A‐H_2_‐350_CO**, Table 1, entry 8). It is red‐shifted with respect to Pt^0^‐CO on platinum particles, indicating terminal chemisorption on electron‐rich platinum sites [62, 63]. No bridging CO bands are observed, probably due to blocking of neighboring sites. The reactions of **A** with hydrogen are summarized in Scheme 3.

**SCHEME 3:**
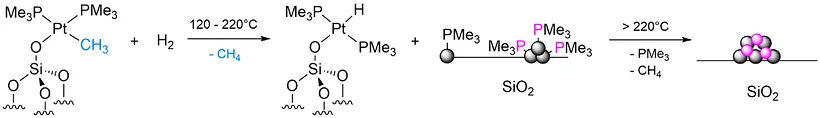
Reaction of **A** with hydrogen at elevated temperatures: formation of the immobilized hydride, phosphine‐decorated clusters, and their decomposition to P‐modified, supported platinum aggregates.

### 2.5 Ligand‐Dependent Reactivity With Hydrogen: Comparison to Other Platinum Methyl Surface Complexes

To evaluate the influence of the ligand sphere of the platinum site, two other methyl platinum surface complexes were prepared, namely PtMe(dmpe)/SiO_2_^700^ (**B**) and PtMe(cod)/SiO_2_^700^ (**C**) (Figure 5). **B** contains a phosphine ligand but does not allow the stabilization of the *trans* orientation of the surface and the hydride. This is supposed to lead to easier reductive elimination of ≡SiO─H from the hydride form, proposed as one driving force leading to the instability of late transition metal hydrides on silica [32]. **C** is a typical example of a supported platinum site, which has been reported to form platinum nanoparticles under hydrogen [41, 45].

**FIGURE 5:**
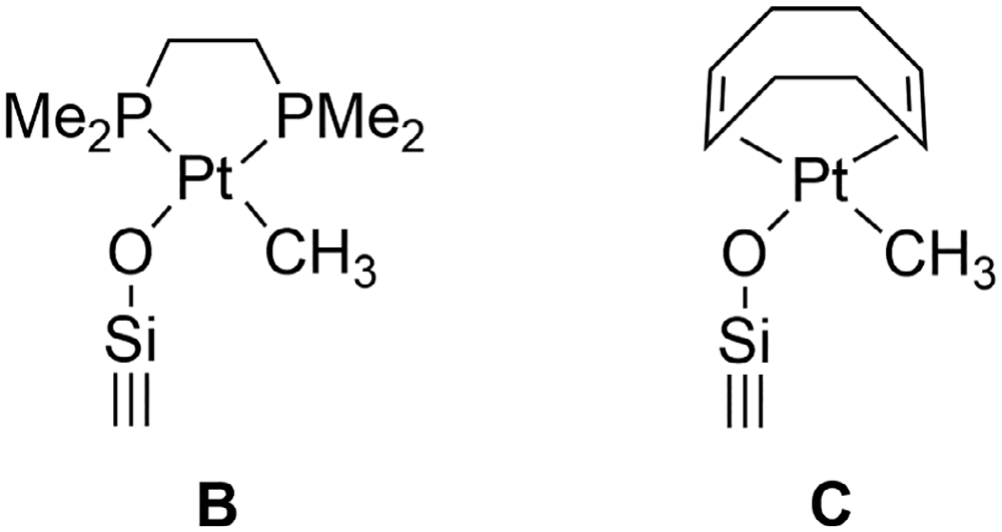
Schematic representation of the silica‐supported platinum methyl complexes PtMe(dmpe)/SiO_2_^700^ (**B**) and PtMe(cod)/SiO_2_^700^ (**C**).

Both grafted materials were obtained from the reaction of SiO_2_^700^ with the dimethyl precursors, as described above. C─H and C═C stretching, as well as deformation bands, are present in the IR spectra of self‐supporting discs (Figures S6 and S7) [2, 43, 45]. Comparable platinum loadings and coverages to **A** were found in elemental analysis of **B** and **C** (Tables S10–S12). The proton‐decoupled ^31^P solid‐state NMR spectrum of **B** has been reported [35]. The spectrum in Figure S15 shows clearly the signal of the phosphine standing *trans* to the surface at 10 ppm (^1^ *J*_PtP_ = 4162 Hz) and that of the P atom *trans* to the methyl group at 35 ppm (^1^ *J*_PtP_ = 1504 Hz). The coupling constants are similar to the *cis* form of **A**. EXAFS data of **C** were analyzed using structural parameters of PtMe(OH)(cod) as starting values for the fitting process (Table S14 and Figure S13) [91]. The complex describes the close surroundings of the Pt(II) center in **C** appropriately (Table S14), while platinum metal foil was not appropriate as a fit.

**B** and **C** react with hydrogen also via the hydrogenolysis of the Pt‐Me bond (Figures S36 and S37) [44]. For compound **C**, the cod ligand desorbs from the surface almost simultaneously with methane formation, already below 100°C. It has been shown that the cod ligand gets hydrogenated under the conditions in an autocatalytic reaction [44]. The alkene ligand is therefore not further able to stabilize the platinum centers. In analogy, lacking of further stabilization by the tmeda ligand of supported palladium on silica led to the faster decomposition of the isolated sites and formation of palladium clusters under CO at room temperature [52].

The formation of platinum nanoparticles from **C** is clearly shown in the X‐ray absorption under hydrogen (Figure S38). The shift of the edge towards the foil and the lower whiteline intensity are indicative of the reduction of the platinum sites (Figure S38). It starts between 40°C and 50°C. The formation of Pt‐Pt contacts is observed in the EXAFS spectra, starting at 65°C. After this reduction, a cluster size of ca. 1.3 nm is deduced from the spectra [92, 93].

Due to the change of the phosphine ligand from monodentate to bidentate in **B**, the in‐situ IR spectrum under hydrogen flow (10 % in helium, 20 mL min^−1^) changes (Figure S39). The band at 2230 cm^−1^ is not observed, neither is a comparable one. In the analogous experiment with deuterium flow (Figure S40), exchange of the ≡SiO─H groups to ≡SiO─D is observed as well [1]. The reaction with **B** starts already at room temperature, leading to the conclusion **B** does in fact react with hydrogen (or deuterium) more easily in the formation of Pt^0^ sites, which are active in the H/D exchange [1]. A material of **B**, which has been pre‐treated at 150°C in a flow of hydrogen (5 mL min^−1^, helium 45 mL min^−1^) for 30 min (**B‐H_2_‐150**), was investigated by solid‐state NMR spectroscopy (Figures S41 and S42). The spectra are comparable to those of the parent material (Figures S14 and S15). In the ^31^P spectrum, the signals of the supported complex at 10 and 35 ppm are observed [35]. The ^1^H MAS NMR spectrum does not show hydride signals in the high field (Figure S41), supporting the hypothesis that the stabilization of the molecular Pt‐H species is not possible by the dmpe ligand.

### 2.6 Isomerization of 1‐Hexene as a Test Reaction

Maier *et al.* described isomerization and cycloisomerization reactions, catalyzed by immobilized Pt‐H species, which were obtained by the oxidative addition of Pt(P*^t^* Bu_3_)_2_ on oxide materials [11]. The authors have shown that the isomerization of 1‐hexene does not proceed with supported platinum particles. The molecular platinum hydride was active, but less than supported, isolated Pt(0) sites, which suffered from aggregation after one cycle [11]. The reaction conditions are summarized in Scheme 4.

**SCHEME 4:**
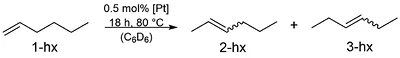
Isomerization of 1‐hexene, catalyzed by supported isolated platinum sites.

The properties of the reaction made it an ideal test for the formed species from **A**, **B**, and **C** under hydrogen (Table 2, for details on the calculations, see Supporting Information). Due to the similarities between the reported species [11] and the found form of **A** under hydrogen, similar activity was expected. In fact, the conversion and TON for **A‐H_2_‐180** are similar to the reported ones on silica and silica‐alumina (Lit.: conversion 5.1 % and 7.0 %, TON 10 and 14) [11]. Pre‐treatment of **A** was necessary for the activity in the reaction, showing the Pt‐H cannot be formed from the Pt‐Me site under reaction conditions. The isolated Pt(0) sites in **A‐H_2_‐350** give higher conversions. Equally, the pre‐treatment of **B** under hydrogen led to higher conversions, in line with the formation of Pt(0) sites. Their nature was tested with CO chemisorption at room temperature. The red‐shifted terminal CO chemisorption band at 2023 cm^−1^ (Table 2) supports this hypothesis. Spectra are in the supporting information (Figure S18). Based on the solid‐state NMR spectra of **B‐H_2_‐150**, a mixture of the supported complex and Pt(0) is expected after the treatment in hydrogen. Reduction of **C** and the formation of platinum metal nanoparticles is responsible for the inactivity of **C**, pre‐treated in hydrogen at 180°C.

**TABLE 2:** Summary of platinum catalysts and results of the isomerization of 1‐hexene.

Catalyst	Pre‐treatment under hydrogen^a^ (temperature (°C), dwell time (min))	Conversion (%)	Selectivity 2‐hexene (%)	TON	TOF (h^−1^)	Terminal υ_CO_ (cm^−1^)
SiO_2_^700^	None	0				
**A**	None	1	75	4	0.2	2092, 2071
**A‐H_2_‐150**	150, 30	3	88	4	0.2	2065, 1990
**A‐H_2_‐180**	180, 30	8	92	11	0.6	2065, 1990
**A‐H_2_‐350**	350, 1	34	92	69	3.8	2040
**B**	None	1	70	3	0.2	2104
**B‐H_2_‐150**	150, 30	43	91	25	1.4	2102, 2023
**B‐H_2_‐180**	180, 30	49	87	62	3.4	2023, 1990
**C**	None	1	61	3	0.2	2086
**C‐H_2_‐150**	150, 30	3	86	8	0.5	2078
**C‐H_2_‐180**	180, 30	1	86	4	0.2	2076

^a^ 5 mL min^−1^, mixed with helium (45 mL min^−1^), heating rate: 5 K min^−1^

Recycling experiments with pre‐treated materials at 180°C have been carried out to test the stability and potential aggregation of the supported platinum sites (Table S18). Leaching of the phosphine complexes was not studied in more detail because it is not suggested from low turnover frequencies, ^31^P NMR spectra of the filtrates, or the reported study [11].

The activity of **B‐H_2_‐180** lowers over the three cycles (Table S18), indicating aggregation, but still active Pt(0) sites in the third cycle. The conversion of **A‐H_2_‐180** drops after the second cycle, which suggests a lack of stability of the supported Pt‐H species in combination with clusters. The Pt‐H species is possibly not sufficiently reformed, which indicates an IR spectrum after an isomerization cycle (Figure S44). **C‐H_2_‐180** is inactive in all cycles, as expected for supported platinum metal nanoparticles. Overall, the test reaction shows that the ancillary ligand influences the species formed under hydrogen and consequently the capability to isomerize 1‐hexene. It suggests the potential for supported PtP_x_ systems in other C─H activation reactions [10, 61].

## 3 Conclusion

Dimethylbis(trimethylphosphine)platinum(II) reacts with partially dehydroxylated silica to form a well‐defined isolated mononuclear surface complex. It represents an interesting model for molecular single‐site platinum catalysts with high reactivity due to the absence of sterically demanding ligands. The monodentate trimethylphosphine ligands can serve as a spectroscopic handle for ^31^P solid‐state NMR spectroscopy; they stabilize the platinum center against agglomeration and strongly influence its reactivity. The persistence of the molecular platinum surface site has been demonstrated in reductive environments, such as hydrogen and carbon monoxide, thereby forming *trans* phosphine complexes. The reactions of the platinum surface species are analogous to the well‐studied chemistry of platinum phosphine complexes in homogeneous solution. Their reactivity contrasts with that of platinum sites bearing chelating ligands (cod, dmpe), which are more readily reduced under hydrogen and tend to agglomerate. The species that form under hydrogen differ with the ancillary ligand of the starting complex. Their different structures and reactivities affect their performance as catalysts for isomerizing 1‐hexene, which requires isolated sites rather than extended metal particles. Hence, taking advantage of phosphine ligands, which are not easily removable, enriches the toolkit for designing catalysts, as they provide stepwise structural information and tend to form isolated sites rather than metal nanoparticles from single‐source precursors.

## 4 Experimental Section

### 4.1 General Annotations

All manipulations involving dimethyl complexes, SiO_2_^700^, and grafted materials were carried out under dry, inert conditions (*Schlenk* line, glovebox) in flame‐dried glassware. The organometallic materials were stored in a freezer (−35°C), inside the glovebox (*MBraun*, Labstar Pro). Argon (purity 5.0, inert gas for the *Schlenk* line and glovebox), helium (purity 4.6), hydrogen (purity 5.0), and 1% CO in helium (purity CO 2.0, purity He 4.6) were purchased from *Westfalen AG*, D_2_ (purity 2.8) from *AirLiquide*. The gases were used without further purification. *N*‐pentane, toluene, and C_6_D_6_ were purchased as anhydrous solvents, degassed by three cycles of *Freeze‐Pump‐Thaw*, and stored over 4 Å molecular sieves. Potassium tetrachloridoplatinate(II) (98 %), trimethylphosphine (1.0 M solution in tetrahydrofuran), methyllithium (1.6 M solution in diethyl ether), methyl‐d_3_‐lithium (0.5 M solution in diethyl ether), fumed silica (0.2‐0.3 µm average particle size), PtMe_2_(cod) (97 %), and 1‐hexene (99 %) were purchased from Sigma–Aldrich, 1,2‐bis(dimethylphosphino)ethane (97 %) from *Arcos Organics*, and ferrocene (99.5 %) from *Alfa Aesar*. The synthesis of PtMe_2_(PMe_3_)_2_ and Pt(CD_3_)_2_(PMe_3_)_2_ was oriented on the literature procedure (details on the proceeding and the characterization in the Supporting Information) [69]. PtMe_2_(dmpe) was prepared according to the literature (NMR spectra and signals in the Supporting Information) [94]. The isomerization of 1‐hexene and recycling experiments were conducted, as described in the literature [11].

### 4.2 Synthesis of Isolated Platinum Surface Complexes

Pre‐treatment of silica: fumed silica was agglomerated in deionized water, dried at 70°C for several days, crushed, and sieved to obtain grain sizes of 100–300 µm. The powder was calcined at 500°C for 8 h (heating rate: 5 K min^−1^). Partial dehydroxylation was carried out at 700°C for 16 h (heating rate: 2 K min^−1^) inside a quartz *Schlenk* tube, which has been interconnected to an oil diffusion pump (10^−5^ mbar, *Ilmvac* 100328). After cooling down, the evacuated flask was transferred into the glovebox. The support material, SiO_2_^700^, was further handled under inert conditions to avoid rehydroxylation [31].

Grafting: the desired amount of the dimethyl platinum(II) precursor (compare Table S8) was dissolved in 5 mL *n*‐pentane or toluene (for PtMe_2_(dmpe)) in a *Schlenk* tube. SiO_2_^700^ (500 mg, n(SiOH): 0.12 mmol [31]) was suspended in 5 mL of the same solvent in a second flask. During cooling by an ice‐water bath, the solution of the precursor was transferred via cannula filtration to the suspension of the support material. The tube and cannula were rinsed with additional 2 mL of the solvent. The mixture was allowed to react for 24 h under occasional shaking and addition of ice to the bath. Then, the supernatant was filtered off. The solid was washed with fresh solvent (*n*‐pentane or toluene, 3 × 5 mL), letting the solvent soak in for 10 min each time. After the last filtration, the colorless solid was dried in vacuo for several hours, yielding the grafted material. Solid‐state NMR, IR, and X‐ray absorption spectra, as well as elemental analysis, are given in the Supporting Information.

### 4.3 NMR Spectroscopy

Liquid‐state NMR spectroscopy: spectra were collected on a *Bruker* AV400 UltraShield NMR spectrometer. Samples were dissolved in at least 0.4 mL of deuterated solvents inside standard or screw‐cap NMR tubes. All chemical shifts are given in δ values (ppm). The residual proton signals of the solvents served as the reference for ^1^H NMR spectra.

Solid‐state NMR spectroscopy: solid‐state ^1^H MAS and ^31^P HPDEC spectra were recorded on a *Bruker* AV300 NMR spectrometer. Samples were densely packed into ZrO_2_ rotors (4 mm diameter) and sealed in the glovebox. The spectra were referenced externally. For ^1^H NMR spectra, adamantane was used (reference signal: 2.00 ppm). The adamantane standard itself was referenced against TMS. For ^31^P NMR spectra, ammonium dihydrogenphosphate was used as the standard (reference signal: 1.11 ppm, referenced to H_3_PO_4_).

### 4.4 Elemental Analysis

Combustion analysis was performed to determine carbon and hydrogen contents, using an elemental analyzer (Euro EA, *HEKATech GmbH*). Platinum and phosphorous contents were determined photometrically on an *Agilent* Cary 100 UV‐vis spectrometer. After boiling the samples in concentrated sulfuric acid under low vacuum, the residues were treated with concentrated H_2_SO_4_, HNO_3,_ and HCl. An aliquot was reacted with an ammonium vanadate solution to quantify the phosphate‐vanadate (absorption at 410 nm). For quantifying [PtCl_6_]^2−^, the sample was reacted with SnCl_2_. Alternatively, the platinum content was determined by Flame Atomic Absorption Spectrometry (F‐AAS, *Agilent* 280FS AA) at 265.9 nm after microwave digestion (*CEM* Discover SP‐D).

### 4.5 (In‐Situ) IR Spectroscopy

Samples were pressed into self‐supporting discs inside the glovebox and transferred inside the gastight IR cell to the *Varian* 670 FT‐IR spectrometer, which was equipped with a liquid‐nitrogen‐cooled mercury cadmium telluride (MCT) detector. The parameters of the measurement were adjusted to the properties of the pellet. In a typical setting, a resolution of 4 cm^−1^, a sensitivity of 8, an aperture value of 2 cm^−1^, and 25 % of the beam intensity were applied. Each spectrum consists of 8 accumulated scans with 25 kHz from 700 to 4000 cm^−1^. For the in‐situ measurements, the cell was connected to an external gas supply. The tubing was purged with helium to avoid air contact with the pellet. The spectrum of the parent material served as the background for the in‐situ spectra. Desorbing species appear as negative bands, and newly formed features as positive ones. The pellet was exposed to 20 mLmin^−1^ total gas flow, which consisted either of 1 % CO in helium, helium, or a mixture of helium with H_2_ or D_2_.

### 4.6 Pre‐Treatments as Temperature‐Programmed Reactions in Combination With Mass Spectrometry

For a temperature‐programmed reaction or pre‐treatment, a U‐shaped glass reactor was typically charged with 100 mg of sample in the glovebox. The reactor was connected to an external gas supply, and the tubing was purged with helium. After the reactor had also been purged with inert gas for 10 min, the flow of the desired gas mixture was set to 50 mL min^−1^, and the sample was heated by 5 K min^−1^ in a tubular furnace (*Carbolite*) to the desired temperature. The composition of the out‐stream was analyzed by mass spectrometry (*Pfeiffer* ThermoStar GSD 320).

### 4.7 (In‐Situ) X‐Ray Absorption Spectroscopy

Ex‐situ samples were placed in glass capillaries (*WJM‐Glas Müller*, Ø 0.7 mm, filling level 40 mm) in the glovebox and sealed by melting. In‐situ samples were placed in open quartz glass capillaries with quartz wool (*WJM‐Glas Müller*, Ø 1.5 mm, filling level 20 mm) and transferred to vials and *Schlenk* flasks under argon atmosphere to the measurement facility.

X‐ray absorption spectroscopy (XAS) experiments at the Pt L_3_‐edge (11564 eV) were carried out at PETRA III beamline P65 at DESY in Hamburg (Germany). The PETRA III storage ring at DESY operates at 6 GeV particle energy with a current of 100 mA in top‐up mode, keeping the flux stable within 1%. The in‐situ samples were measured in quartz glass capillaries without dilution. All spectra were recorded as continuous scans in both fluorescence and transmission modes in the range of −200 to 800 eV around the edge. The fluorescence data were used for data evaluation due to better quality. In addition, Pt‐foil was measured simultaneously during the sample measurements as a reference. For beamline P65, an 11‐period mini‐undulator was the photon source, which provided a moderate photon flux density, and the incident energy was selected using a water‐cooled Si(311) double crystal monochromator. All ex‐situ samples were measured in pure form in sealed capillaries. For every sample, both a X‐ray absorption near‐edge structure (XANES) and an extended X‐ray absorption fine structure (EXAFS) analysis were carried out.

The data treatment was performed using the Demeter software package [95]. The first inflection point was taken as edge energy E_0_. No phase shift corrections have been applied. The EXAFS analysis was performed using Artemis software. For the fitting procedure, the amplitude reduction factor S_0_^2^ for the Pt reference foil was determined and used as a fixed parameter for all materials.

### 4.8 Computational Details

Density functional theory (DFT) calculations were carried out, using the Gaussian software package [96]. Geometry optimizations and frequency calculations were performed with B3LYP as the functional. The LANL2DZ basis set and its associated effective core potential (ECP) were employed for Pt, while the other elements (C, H, O, Si, P) were described by 6–31+G(d). Isotopologues were specified by the Iso keyword in the input orientation. Further information, Cartesian coordinates, and selected harmonic frequencies are given in the Supporting Information.

## Conflicts of Interest

The authors declare no conflicts of interest.

## Supporting information




**Supporting File 1**: chem71409‐sup‐0001‐SuppMat.pdf

## Data Availability

The data that support the findings of this study are available from the corresponding author upon reasonable request.
