# Supplementary material for: Silica‐Supported Mononuclear Methyl‐Platinum Complexes Stabilized by Trimethylphosphine—Synthesis, Structure, and Reactions Under Reducing Conditions
Source: Chemistry. 2026 Jul 10;32(39):e71409. doi: 10.1002/chem.71409 (PMC13715464; doi:10.1002/chem.71409)
Supplement: Supplementary file 1 [file CHEM-32-e71409-s001.pdf]

Silica-Supported Mononuclear Methyl-Platinum Complexes Stabilized by Trimethylphosphine – Synthesis, Structure, and Reactions under Reducing Conditions

Supporting Information

Lea S. Kopietz, [a] Sven Schaefer, [b] Oksana Storcheva, [a] Gabriele Raudaschl-Sieber, [a] Johannes Bitzer, [b] Wolfgang Kleist, [b] and Klaus Köhler*[a]

[a] Department of Chemistry, TUM School of Natural Sciences, Lichtenbergstr. 4, 85748 Garching, Catalysis Research Center, Ernst-Otto-Fischer-Str. 1, 85748 Garching, Technical University of Munich, Germany

[b] Department of Chemistry, RPTU Kaiserslautern-Landau, Erwin-Schrödinger-Str. 54, 67663 Kaiserslautern, Germany

* Corresponding author: klaus.koehler@tum.de

Contents

Supplementary material for the synthesis and characterization of platinum surface complexes 2

Synthesis and characterization of dimethyl precursors 2

Grafting reaction of $PtMe_2(PMe_3)_2$ on NMR-scale 6

Characterization of silica-supported platinum surface complexes 7

Supplementary material: Reactivity of A with carbon monoxide 23

Supplementary material: Reactivity of A under inert gas 26

Supplementary material: Reactivity of A with hydrogen 29

Supplementary material: Reactivity of B and C with hydrogen 34

References 41

Supplementary material for the synthesis and characterization of platinum surface complexes

Synthesis and characterization of dimethyl precursors

$PtCl_2(PMe_3)_2$: (adapted)[1] Under argon atmosphere, potassium tetrachloridoplatinate(II) (1.0 g, 2.4 mmol, 1.0 eq.) was stirred with trimethylphosphine (5.3 mL of a 1.0 M solution in tetrahydrofuran, 5.3 mmol, 2.2 eq.) in Ar-saturated, deionized water at room temperature for 3 h. After removing the solvent, diethyl ether was added. It dissolves yellow *trans*-$PtCl_2(PMe_3)_2$, which forms in small amount. It is removed by filtering off the ethereal solution. The dried, beige residue, which contains *cis*-$PtCl_2(PMe_3)_2$, together with potassium chloride and possibly unreacted $K_2[PtCl_4]$, was used for methylation. For the stoichiometric calculation of the following reactions, $PtCl_2(PMe_3)_2{\bullet}2KCl$ (M: 567.24 mg $mmol^{-1}$) was assumed.

$PtMe_2(PMe_3)_2$: (adapted)[2] *Cis*-$PtCl_2(PMe_3)_2$ (700 mg, 1.2 mmol Pt, 1.0 eq.) was suspended in toluene (15 mL). After cooling with an ice-water bath, methyllithium (2.5 mL of a 1.6 M solution in diethyl ether, 4.0 mmol, 3.3 eq.) was added dropwise to the mixture. The reaction is stirred for 1.5 h. *N*-pentane (10 mL) was added before slowly hydrolyzing the excess of methyllithium with water (7 mL). Freezing out the aqueous phase allowed filtering off the organic layer, which was evacuated to dryness under cooling. It yielded a colorless solid (200 mg, 0.5 mmol, 43 %), which was analyzed by elemental analysis and NMR spectroscopy in solution (^1H, ^{31}P, Figure S 1, Figure S 2).

Elemental analysis: calc.: found (wt%): C 25.47: 26.13, H 6.41: 6.59.

^{1}H NMR (400 MHz, C_6D_6, r. t.): δ (ppm) = 1.15- 1.10 (m, 6 H, $^2J_{PtH}$ = 66 Hz, Pt-Me), 1.05 (d, 12 H to 14 H instead of 18 H: shorter integration limits due to overlay with ^{195}Pt coupled signal, $^2J_{PH}$ = 7.7 Hz, $^3J_{PtH}$ = 20 Hz, Pt-PMe_3). **^{31}P{^{1}H} NMR** (162 MHz, C_6D_6, r. t.): δ (ppm) =-24.0 (s, $^1J_{PtP}$ = 1785 Hz).

$Pt(CD_3)_2(PMe_3)_2$: The procedure follows the description of $PtMe_2(PMe_3)_2$ in smaller scale. 250 mg *cis*-$PtCl_2(PMe_3)_2$ (0.4 mmol Pt, 1.0 eq.) in 5 mL toluene were reacted with methyl-d_3-lithium (2 mL of a 0.5 M solution in diethyl ether, complex with lithium iodide, 1.0 mmol, 2.5 eq.). After stirring for 2 h, hydrolysis, filtration, and drying, a colorless solid was obtained.

Elemental analysis: ($Pt(CD_3)_2(PMe_3)_2{\cdot}0.05\ PtI(CD_3)(PMe_3)_2$) calc.: found (wt%): C 14.05: 16.34, H 4.40: 4.82.

NMR: Figure S 1, Figure S 2. **^{1}H NMR** (400 MHz, C_6D_6, r. t.): δ (ppm) = 1.05 (d, 18 H, $^2J_{PH}$ = 7.6 Hz, $^3J_{PtH}$ = 20 Hz, Pt-PMe_3). **^{31}P{^{1}H} NMR** (162 MHz, C_6D_6, r. t.): δ (ppm) =-23.9 ($^1J_{PtP}$ = 1776 Hz).

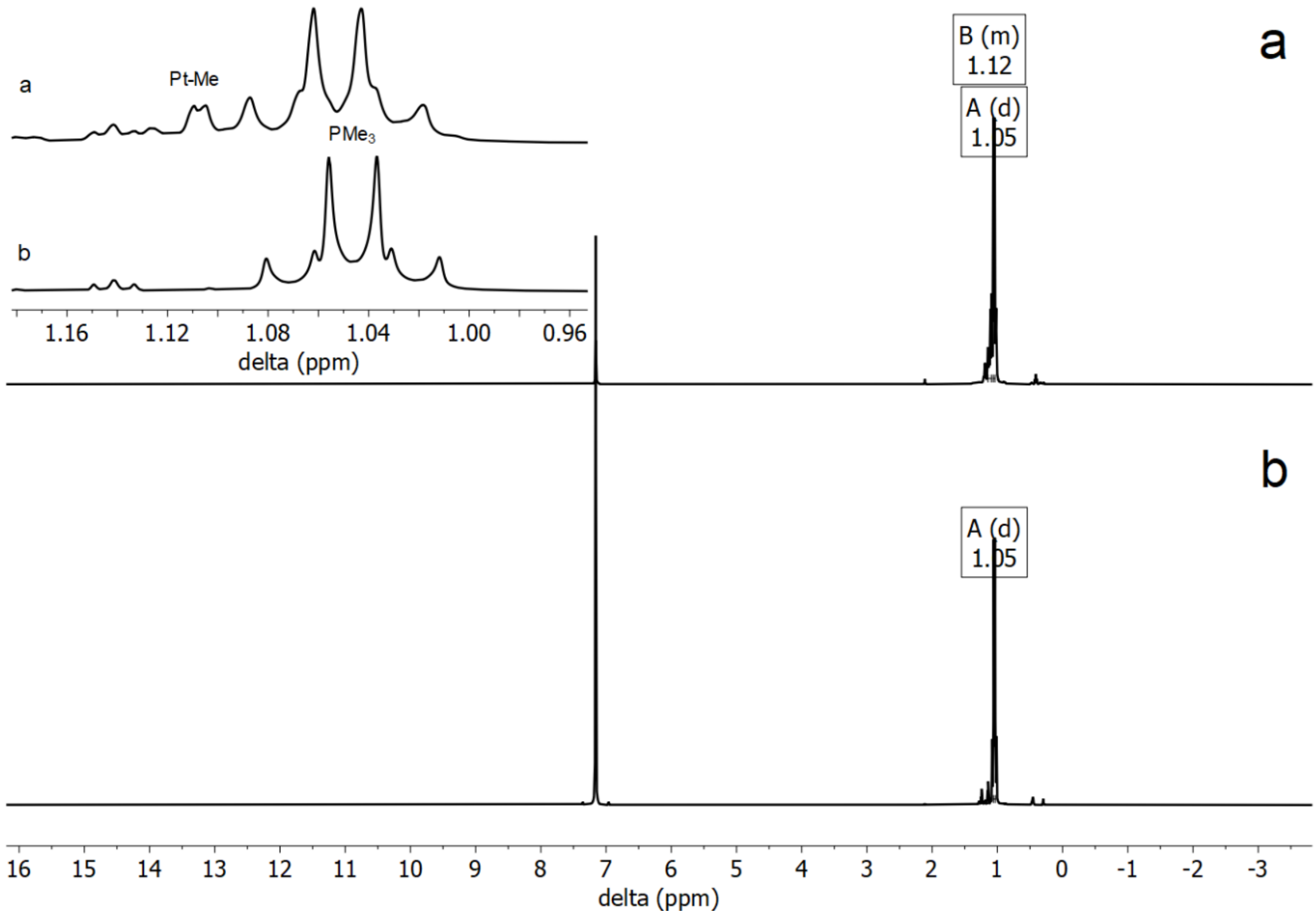

Figure S 1. ^{1}H NMR spectra (400 MHz, C_6D_6, r. t.) of $PtMe_2(PMe_3)_2$ (a) and $Pt(CD_3)_2(PMe_3)_2$ (b): A -P(C<u>H</u>$_3$)$_3$, B -C<u>H</u>$_3$.

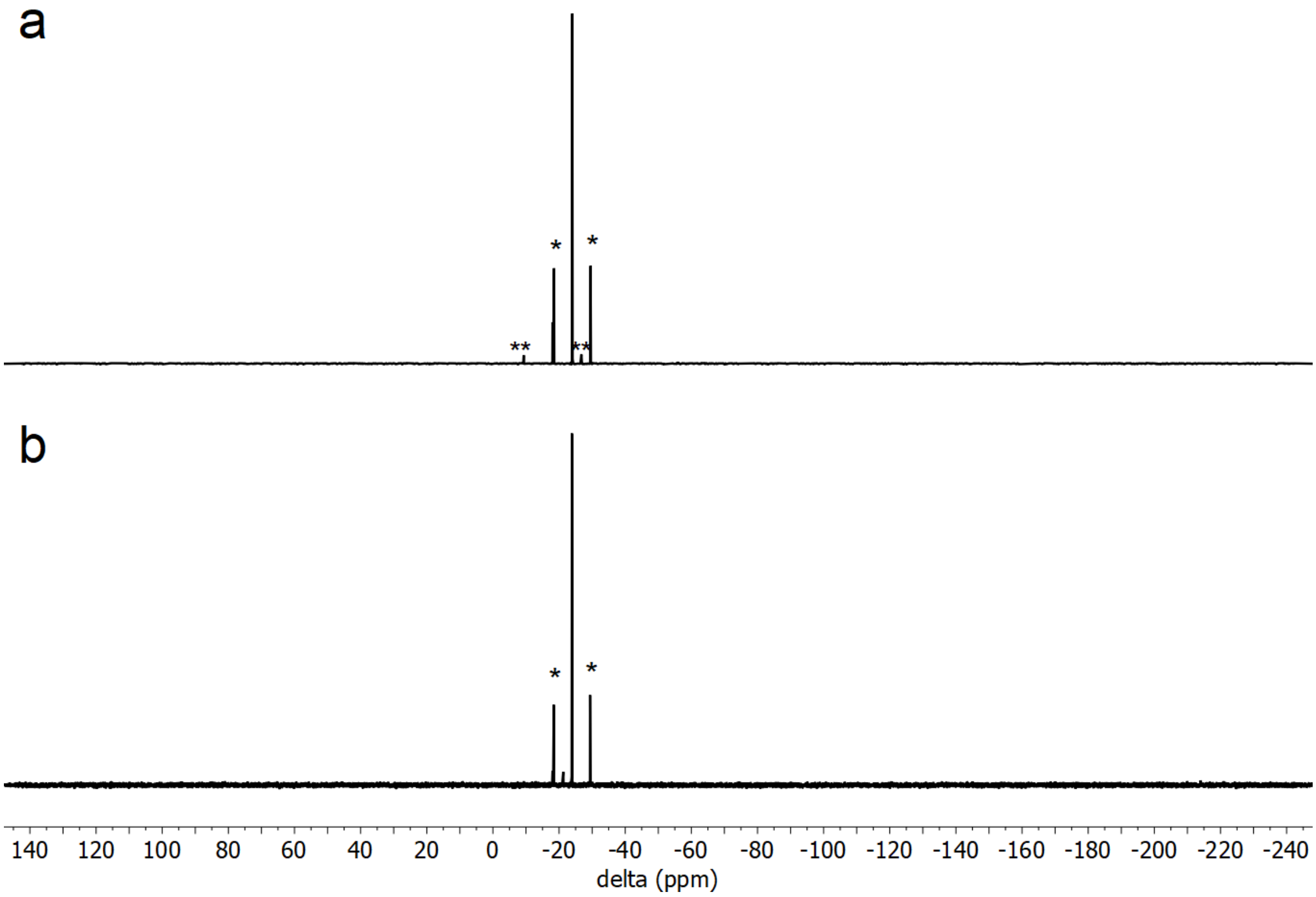

Figure S 2. $^{31}P\{^1H\}$ NMR spectra (162 MHz, C_6D_6, r. t.) of a) *cis*-$PtMe_2(PMe_3)_2$: δ = -24.0 ppm ($^1J_{PtP}$ = 1785 Hz), and b) *cis*-$Pt(CD_3)_2(PMe_3)_2$: δ = -23.9 ppm ($^1J_{PtP}$ = 1776 Hz); ^{195}Pt satellites marked with an asterisk; minor signals are possibly mono-methylated species.

$PtMe_2$(dmpe): $PtMe_2$(dmpe) was synthesized *via* ligand exchange of $PtMe_2$(cod) with 1,2-bis(dimethylphosphino)ethane, according to literature.[3] The obtained colorless solid was analyzed by elemental analysis and NMR spectroscopy in solution (^{1}H, ^{31}P, Figure S 3, Figure S 4). The methods showed small impurities of silicone grease and the free cod ligand. **Elemental analysis:** calc./ found (wt%): C 25.60: 27.01, H 5.91: 6.05.

^{1}H NMR (400MHz, C_6D_6, r. t.): δ (ppm) = 1.26 (*pseudo*-t, $^3J_{PH}$ = 7.9 Hz, $^2J_{PtH}$ = 69.0 Hz, 6 H, Pt−Me), 0.99 (d, $^2J_{PH}$ = 8.4 Hz, $^3J_{PtH}$ = 21.9 Hz, 12 H, Pt-PMe$_2$), 0.84–0.78 (m, 4 H, Pt-P-CH$_2$). **^{31}P{^{1}H} NMR** (162 MHz, C_6D_6, r. t.): δ (ppm) = 25.0 (s, $^1J_{PtP}$ = 1730 Hz).

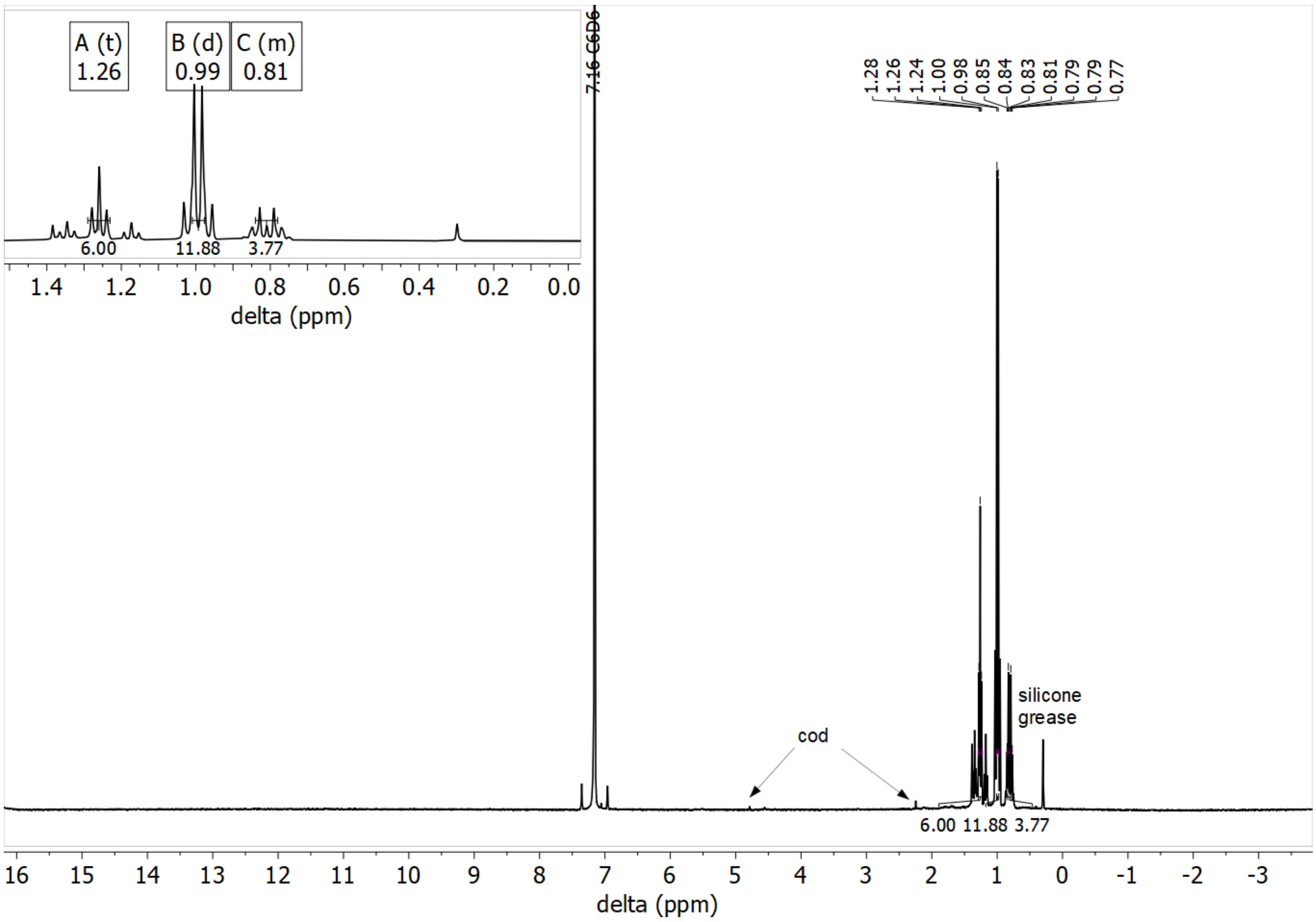

Figure S 3. ^{1}H NMR spectrum (400 MHz, C_6D_6, r. t.) of $PtMe_2$(dmpe).

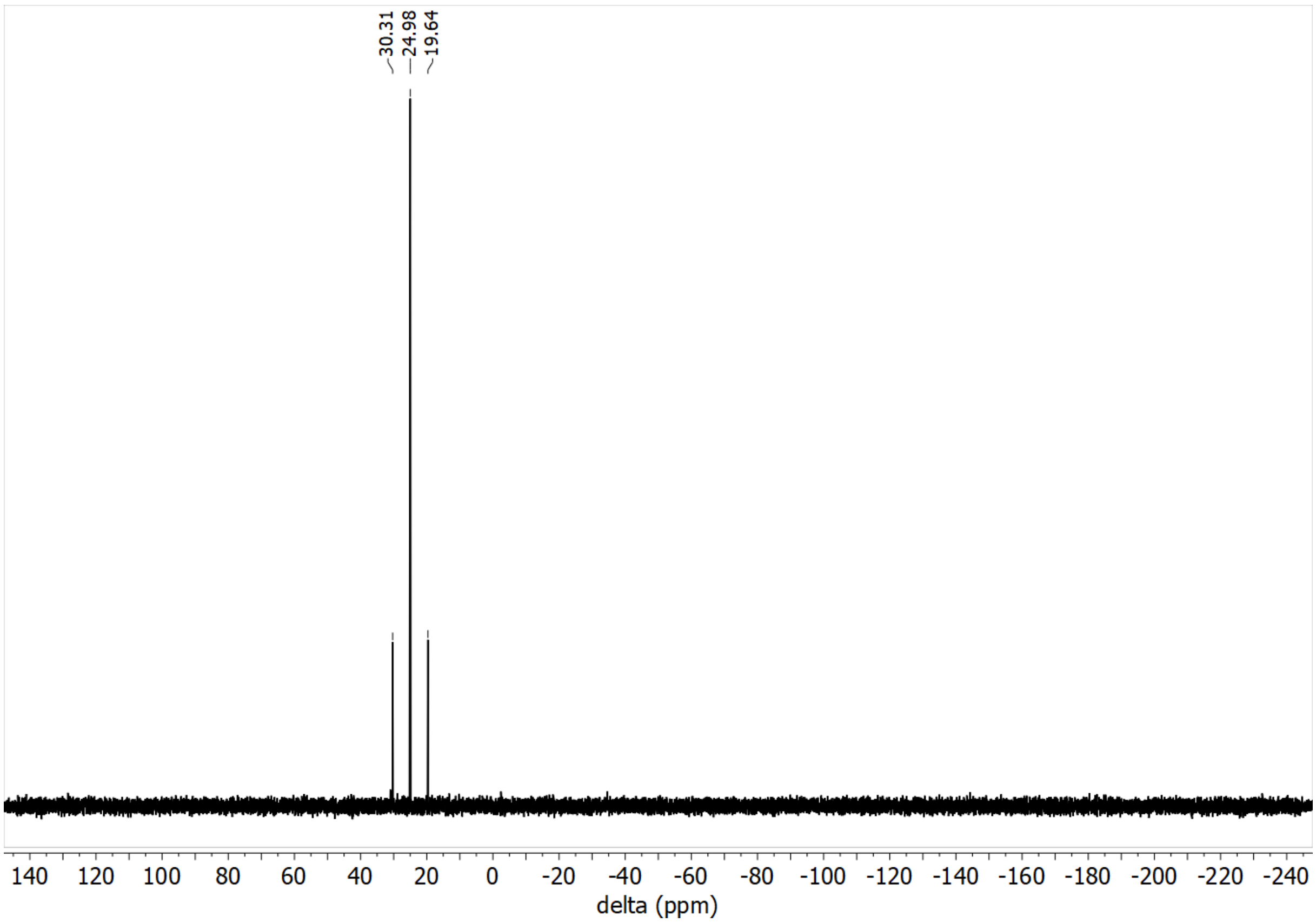

Figure S 4. $^{31}P\{^1H\}$ NMR spectrum (162 MHz, C_6D_6, r. t.) of $PtMe_2$(dmpe) with ^{195}Pt satellites at 19.6 ppm and 30.3 ppm ($^1J_{PtP}$ = 1730 Hz).

Grafting reaction of $PtMe_2(PMe_3)_2$ on NMR-scale

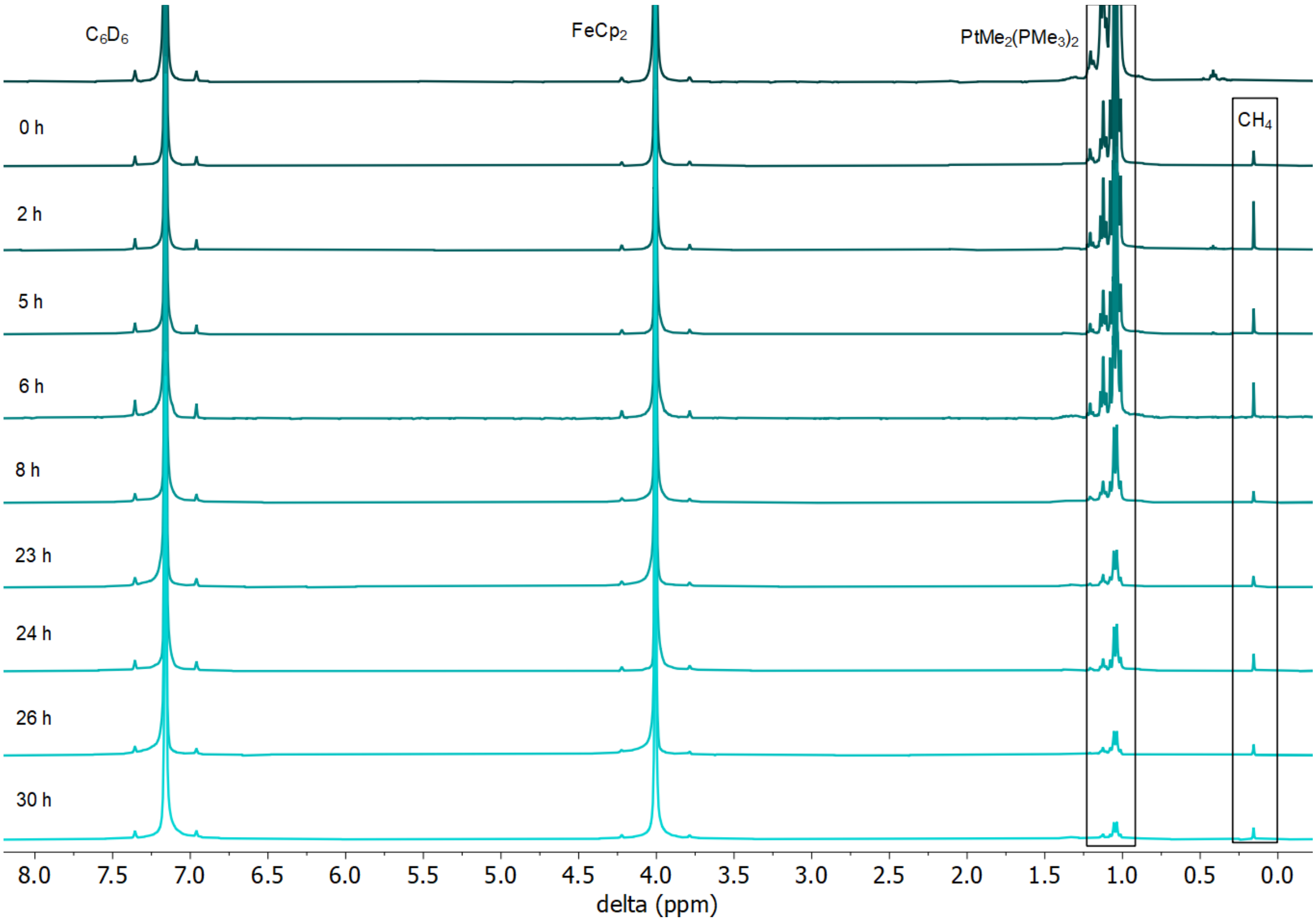

Figure S 5. Time-dependent ^1H NMR spectra (400 MHz, C_6D_6, r. t.): Monitoring the consumption of $PtMe_2(PMe_3)_2$ and the evolution of methane during the grafting reaction in NMR scale at room temperature, after the addition of SiO_2^{700} to the precursor solution at the relative time 0 h. Ferrocene is added as an internal standard.

Characterization of silica-supported platinum surface complexes

FT-IR spectra of $PtCD_3(PMe_3)_2/SiO_2^{700}$, $PtMe(dmpe)/SiO_2^{700}$, and $PtMe(cod)/SiO_2^{700}$

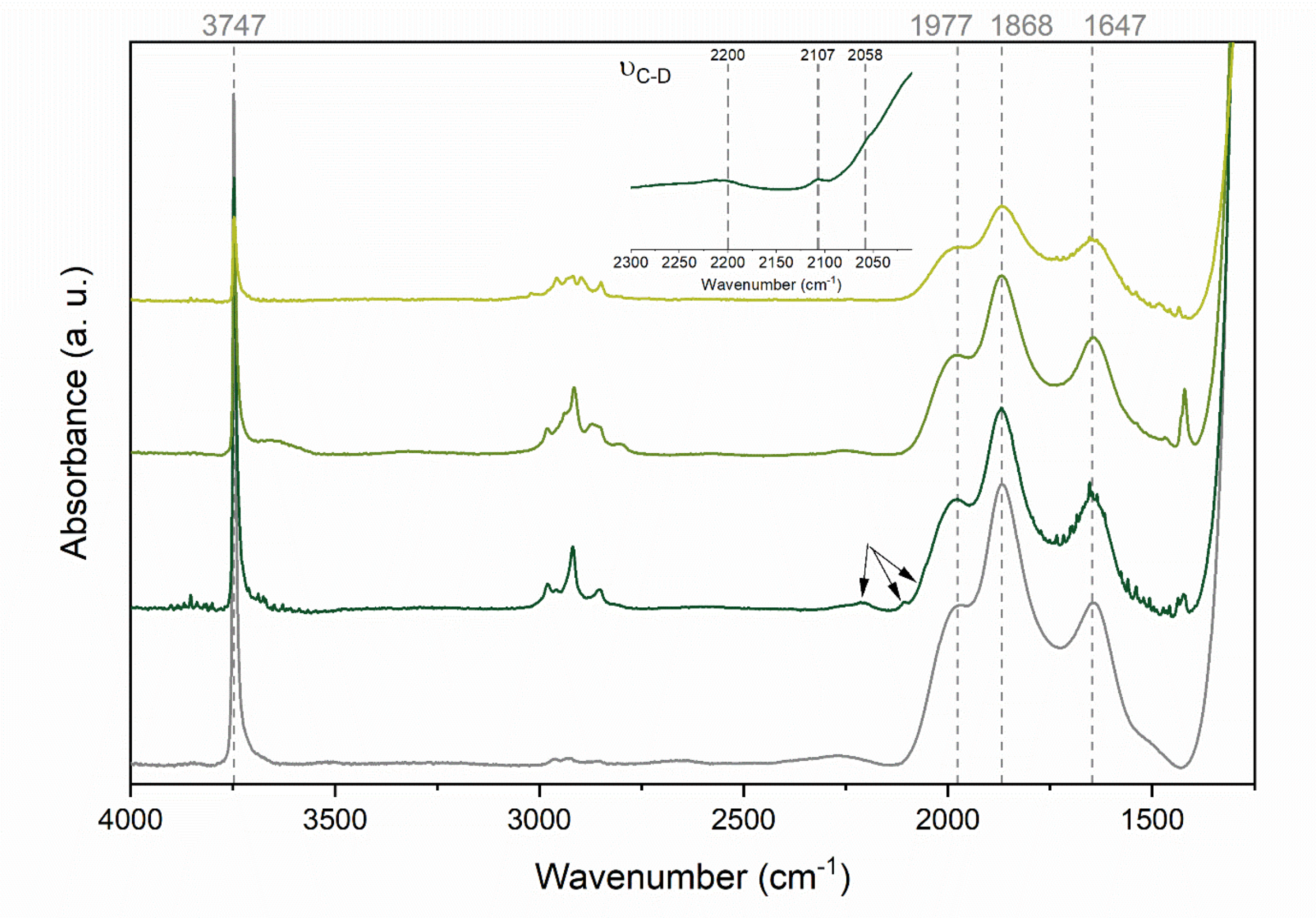

Figure S 6. FT-IR spectra of self-supporting discs of SiO_2^{700} (grey), $PtCD_3(PMe_3)_2/SiO_2^{700}$ (**A-d$_3$**, dark green), $PtMe(dmpe)/SiO_2^{700}$ (**B**, green), and $PtMe(cod)/SiO_2^{700}$ (**C**, light green); inset: enlarged region of the C-D stretching vibrations in **A-d$_3$**.

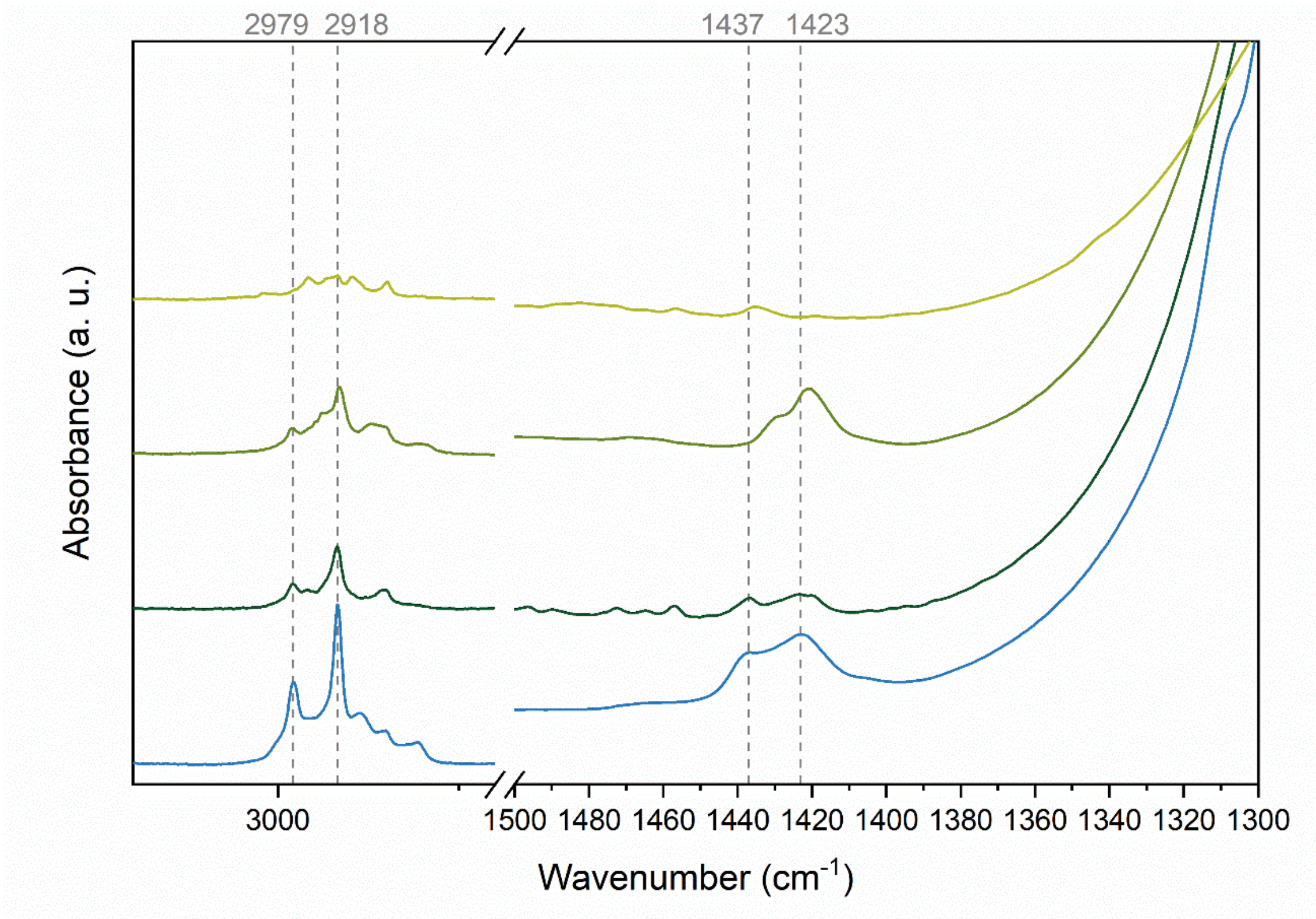

Figure S 7. FT-IR spectra of self-supporting discs of PtMe(PMe$_3$)$_2$/SiO$_2^{700}$R (**A**, blue), PtCD$_3$(PMe$_3$)$_2$/SiO$_2^{700}$ (**A-d$_3$**, dark green), PtMe(dmpe)/SiO$_2^{700}$ (**B**, green), and PtMe(cod)/SiO$_2^{700}$ (**C**, light green), enlarged in the region of C-H stretching, C=C stretching (1475, 1435 cm^{-1}),[4] and methyl deformation vibrations. Marked bands are assigned to P-Me groups, based on the reported values in ref.[5] and comparison of the spectra of **A**, **A-d$_3$**, and **B** (its deformation bands are slightly red-shifted)

Assignment of FT-IR bands of PtMe(PMe$_3$)$_2$/SiO$_2^{700}$ and Pt(CD$_3$)(PMe$_3$)$_2$/SiO$_2^{700}$, assisted by DFT calculations

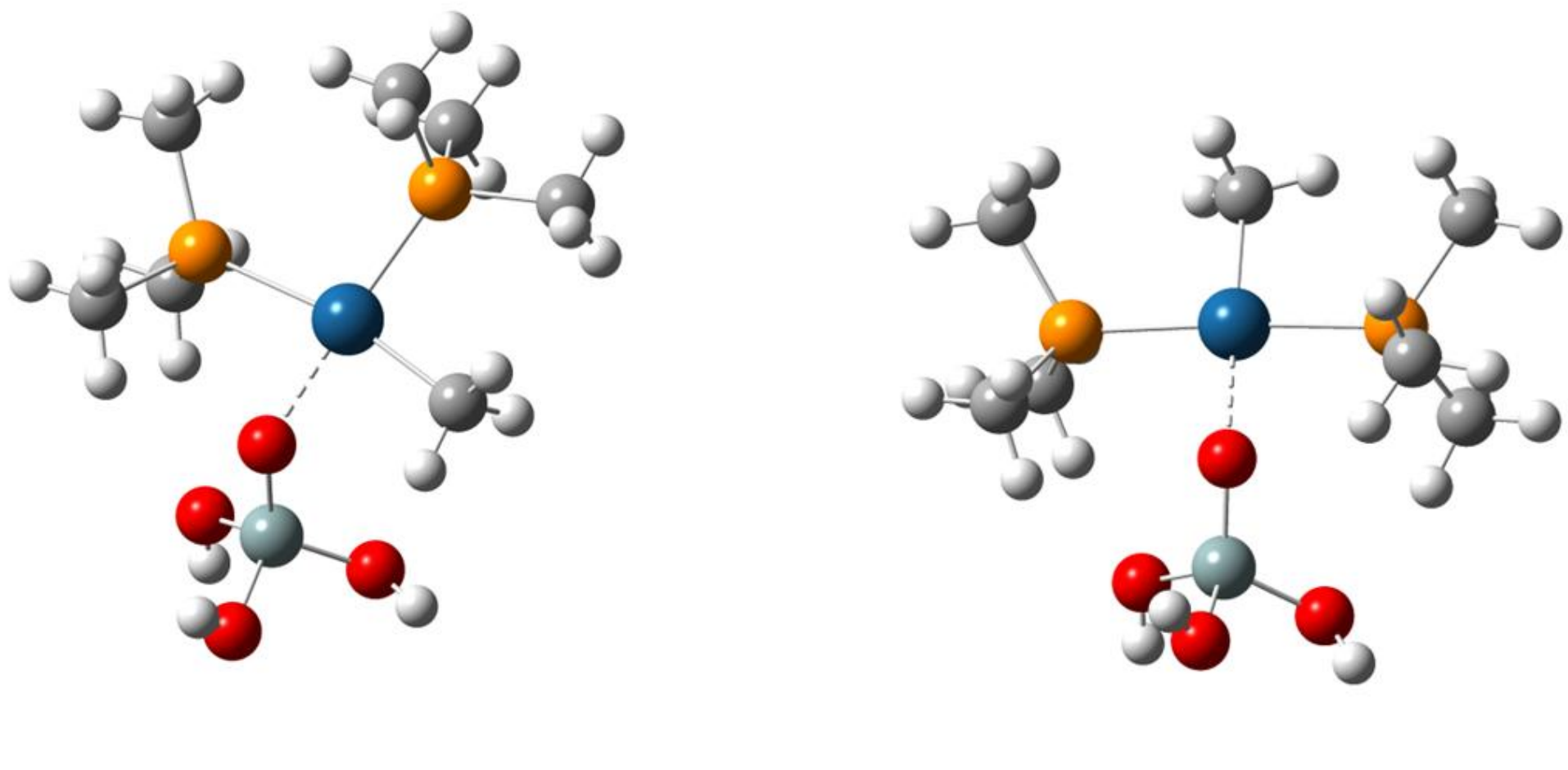

Figure S 8. Geometry-optimized DFT structures as models for the surface complexes in **A**: Pt depicted in blue, P in orange, C in grey, Si in grey, surrounded by O in red, and H in white; calculations were carried out with B3LYP as functional, LANL2DZ with the associated effective core potential for Pt and 6-31+G(d) for the other elements.

To assist assigning experimental IR bands of PtMe(PMe$_3$)$_2$/SiO$_2^{700}$ and Pt(CD$_3$)(PMe$_3$)$_2$/SiO$_2^{700}$, DFT model structures (PtMe(PMe$_3$)$_2$-OSi(OH)$_3$ with PMe$_3$ in *cis* and

trans orientation) were applied. Geometry-optimizations and harmonic frequency calculations were carried out in the Gaussian program suite,[6] using the B3LYP functional,[7,8] LANL2DZ with the associated effective core potential (ECP) for Pt,[9] and 6-31+G(d) for other elements as the mixed basis set.[10] The geometry-optimized structures **A-1** and **A-2** (Figure S 8, Table S 2, Table S 3) were identified as stationary points and confirmed as minima by the absence of imaginary frequencies. Based on these structures, calculations on the same level of theory were carried out for the isotopologues. In these structures, $-CH_3$ was substituted as $-CD_3$, using the Iso=2.01 keyword for each H of the platinum-methyl group in the input. The optimized structures (**A-1-CD₃**, Table S 4; **A-2-CD₃**, Table S 5) and calculated frequencies of the substituted models gave isotope shifts of 856 and 847 cm^{-1} for the symmetric C-H/ C-D vibrations (Table S 1). Involvement of both methyl groups, PMe_3 and Pt-Me, in asymmetric C-H stretching vibrations was grasped by visualizing the vibrations in *GaussView*. Calculated gas-phase IR spectra were scaled by 0.961,[11] and are in good accordance with the experimental ones (Figure S 9, Figure S 10). Especially in the C-H stretching region of **A-1** and **A-2**, bands of the calculated isomers overlay (Figure S 9). The bands around 2850 cm^{-1} and 2800 cm^{-1} are not present in the simulated spectra (Figure S 9, Figure S 10). They are assigned to the Fermi resonance of methyl deformation vibrations of trimethylphosphine (2*1423 cm^{-1}) and a methyl first overtone, which are not represented by the harmonic calculations.

Table S 1. Calculated symmetric C-H/ C-D stretching frequencies of the $Pt-CH_3$/ $Pt-CD_3$ groups

Optimized geometry	Calculated symmetric C-H stretching of $Pt-CH_3$ (cm^{-1})	Calculated symmetric C-D stretching of $Pt-CD_3$ (cm^{-1})	Calculated isotope shift Δ (cm^{-1})	Scaled frequencies (cm^{-1}) υ_s(C-H), υ_s(C-D)
Cis-standing PMe_3 ligands (**A-1**/ **A-1-CD₃**)	3022.34	2166.24	856.10	2904.47, 2081.76
Trans-standing PMe_3 ligands (**A-2**/ **A-2-CD₃**)	3001.46	2154.80	846.66	2884.40, 2070.76

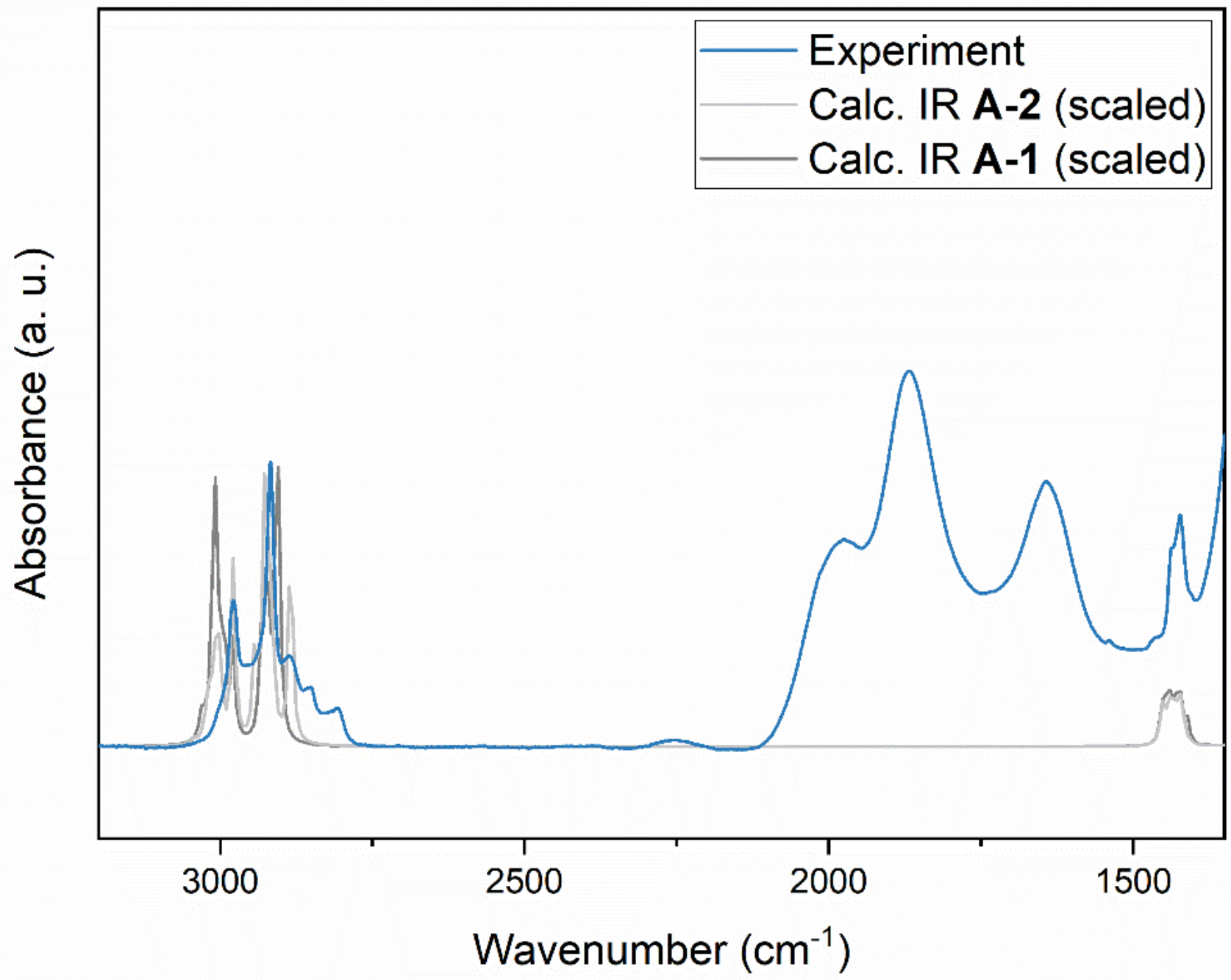

Figure S 9. Simulated gas-phase IR spectra of the geometry-optimized structures **A-1** and **A-2** (grey), compared to the experimentally obtained IR spectrum of **A** (blue). The calculated spectra are scaled by 0.961.

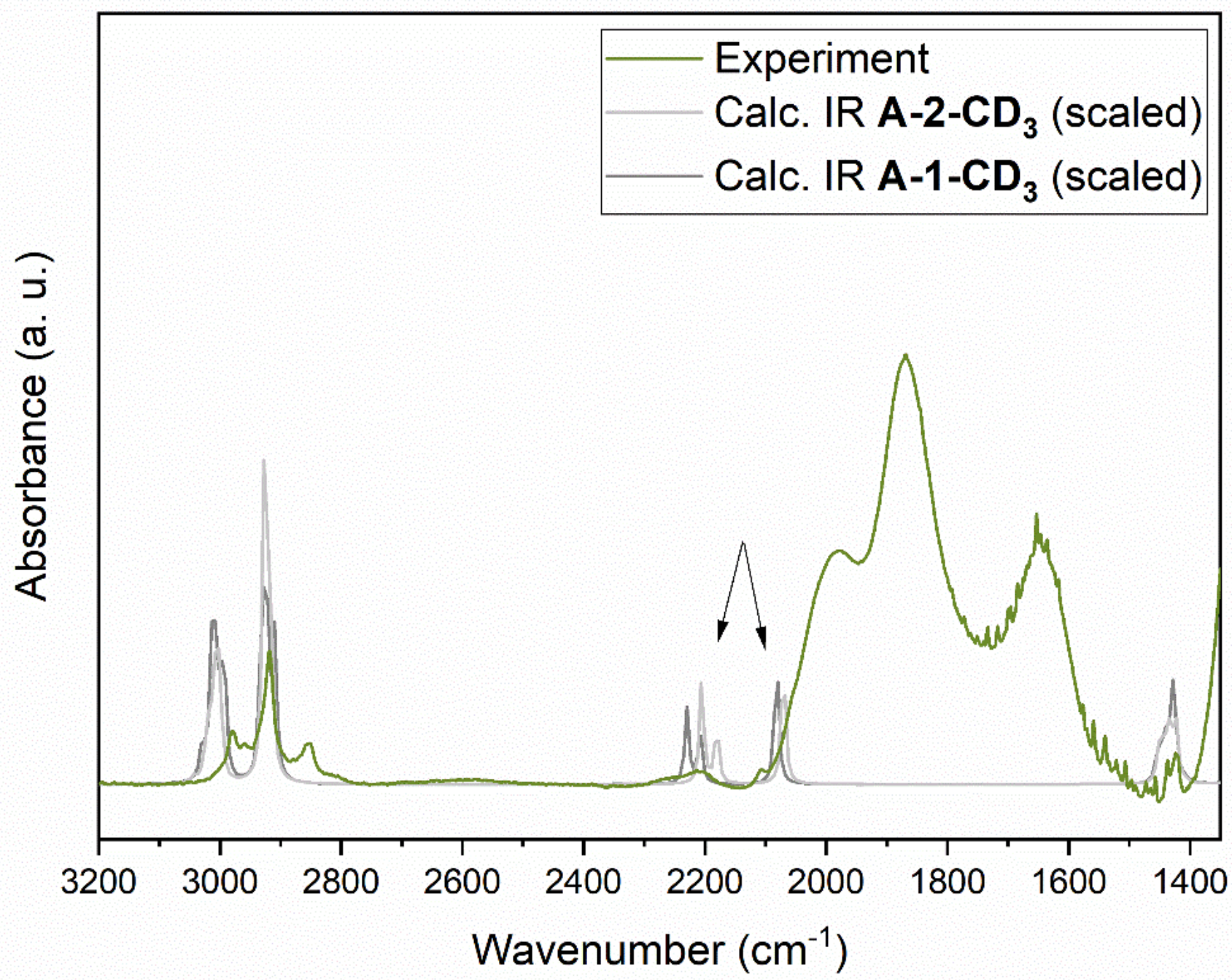

Figure S 10. Simulated gas-phase IR spectra of **A-1-CD$_3$** and **A-2-CD$_3$** (grey), which are scaled by 0.961 and compared to the experimental IR spectrum of **A-d$_3$** (green). The arrows mark calculated asymmetric and symmetric C-D stretching vibrations of the Pt-CD$_3$ groups in the isomers.

Table S 2. Cartesian coordinates of the geometry-optimized structure **A-1**

Center number	Atomic number	Coordinates (Angstroms)		
		X	Y	Z
1	78	-0.317588	-0.360225	-0.250780
2	8	1.733716	-0.156601	-0.662650
3	15	-0.142438	2.063342	-0.044227
4	15	-2.492460	-0.668192	0.231911
5	6	-0.063205	-2.409745	-0.560304
6	1	-0.059029	-2.958531	0.392359
7	1	-0.817395	-2.853883	-1.222977
8	1	0.917718	-2.512741	-1.037105
9	6	-3.714196	0.124070	-0.907569
10	1	-4.743406	-0.087004	-0.588375
11	1	-3.564289	1.208020	-0.932269
12	1	-3.566574	-0.264855	-1.921716
13	6	-3.135195	-2.396362	0.306813
14	1	-2.536387	-2.990759	1.004885
15	1	-4.181393	-2.393534	0.638368
16	1	-3.075069	-2.868385	-0.679292
17	6	-2.987346	-0.029267	1.894771
18	1	-2.780205	1.043346	1.968634
19	1	-4.055473	-0.200788	2.082289
20	1	-2.400074	-0.541853	2.665337
21	6	-1.599397	3.216634	-0.066057
22	1	-2.154638	3.107634	-1.005635
23	1	-2.281852	3.008271	0.766674
24	1	-1.265917	4.259779	0.017649
25	6	0.874844	2.747311	-1.420914
26	1	1.104551	3.808385	-1.256827
27	1	1.796694	2.161571	-1.477762
28	1	0.334525	2.635543	-2.368826
29	6	0.755923	2.611924	1.470039
30	1	1.722148	2.094679	1.517124
31	1	0.917701	3.698611	1.458109
32	1	0.173382	2.347267	2.361404
33	14	3.044691	-0.546219	0.173909
34	8	3.456873	0.730049	1.178711
35	1	4.167779	0.558937	1.808042
36	8	3.009734	-1.846814	1.220744
37	1	3.114601	-2.721815	0.830329
38	8	4.295234	-0.890395	-0.888608
39	1	4.149237	-0.597863	-1.795803

Table S 3. Cartesian coordinates of the geometry-optimized structure **A-2**

Center number	Atomic number	Coordinates (Angstroms)		
		X	Y	Z
1	78	0.025891	-0.757205	-0.029065
2	8	0.130126	1.316713	-0.715793
3	6	-0.029157	-2.737056	0.579291
4	1	-0.265366	-3.390466	-0.275730
5	1	0.921516	-3.082948	1.003143
6	1	-0.795492	-2.905533	1.351231
7	14	-0.062014	2.708511	0.029628
8	8	1.146775	3.207317	1.089025
9	1	1.659810	3.964370	0.781337
10	8	-0.110043	3.988636	-1.058961
11	1	-0.342147	3.757654	-1.965637
12	8	-1.465887	2.685481	0.954824

13	1	-1.574851	3.422007	1.568457
14	15	2.361501	-0.650926	-0.053163
15	15	-2.311977	-0.708285	-0.071846
16	6	3.350948	-2.207831	0.037493
17	1	4.420185	-1.988425	-0.079988
18	1	3.198471	-2.702308	1.003373
19	1	3.037865	-2.898749	-0.753795
20	6	2.978658	0.148461	-1.592620
21	1	2.415537	1.075968	-1.736303
22	1	4.055262	0.355344	-1.531966
23	1	2.783731	-0.508542	-2.448597
24	6	3.070681	0.369620	1.306970
25	1	4.156556	0.484613	1.185886
26	1	2.588035	1.354112	1.313008
27	1	2.867603	-0.118281	2.267979
28	6	-3.153454	-2.266758	-0.595558
29	1	-2.858759	-3.095994	0.056115
30	1	-4.244404	-2.150356	-0.556684
31	1	-2.857338	-2.516641	-1.621211
32	6	-3.046690	0.551283	-1.194638
33	1	-4.138894	0.442579	-1.229883
34	1	-2.789476	1.546207	-0.820257
35	1	-2.633433	0.434701	-2.202504
36	6	-3.078406	-0.325548	1.559359
37	1	-2.715535	0.658900	1.874848
38	1	-4.174497	-0.311629	1.490584
39	1	-2.771820	-1.073788	2.299480

Table S 4. Cartesian coordinates of the geometry-optimized structure **A-1-CD$_3$**, atomic numbers 1-D denote isotopologues

Center	Atomic	Coordinates (Angstroms)		
number	number	X	Y	Z
1	78	-0.317474	-0.360186	-0.251029
2	8	1.733695	-0.157178	-0.663146
3	15	-0.142231	2.063180	-0.044246
4	15	-2.492362	-0.668000	0.232054
5	6	-0.063499	-2.409757	-0.560588
6	1-D	-0.059411	-2.958605	0.392039
7	1-D	-0.817818	-2.853762	-1.223227
8	1-D	0.917372	-2.512941	-1.037454
9	6	-3.714283	0.123459	-0.907816
10	1	-4.743443	-0.087394	-0.588321
11	1	-3.564377	1.207394	-0.933362
12	1	-3.566794	-0.266214	-1.921697
13	6	-3.135005	-2.396169	0.308028
14	1	-2.536209	-2.990117	1.006495
15	1	-4.181201	-2.393094	0.639589
16	1	-3.074880	-2.868846	-0.677758
17	6	-2.987330	-0.028297	1.894606
18	1	-2.780283	1.044366	1.967988
19	1	-4.055452	-0.199815	2.082136
20	1	-2.400063	-0.540478	2.665445
21	6	-1.599404	3.216099	-0.068111
22	1	-2.153298	3.106727	-1.008447
23	1	-2.282971	3.007654	0.763682
24	1	-1.266393	4.259367	0.015888
25	6	0.876787	2.747464	-1.419471
26	1	1.105423	3.808761	-1.255322
27	1	1.799157	2.162404	-1.474739

28	1	0.338082	2.634979	-2.368214
29	6	0.754209	2.611638	1.471200
30	1	1.720623	2.094771	1.518879
31	1	0.915548	3.698396	1.459966
32	1	0.170979	2.346261	2.361897
33	14	3.044418	-0.546289	0.174024
34	8	3.455874	0.730404	1.178639
35	1	4.166570	0.559474	1.808276
36	8	3.009401	-1.846609	1.221185
37	1	3.114961	-2.721636	0.831008
38	8	4.295420	-0.890549	-0.887905
39	1	4.149952	-0.598012	-1.795178

Table S 5. Cartesian coordinates of the geometry-optimized structure **A-2-CD$_3$**, atomic numbers 1-D denote isotopologues

Center number	Atomic number	Coordinates (Angstroms)		
		X	Y	Z
1	78	0.025891	-0.757205	-0.029065
2	8	0.130126	1.316713	-0.715793
3	6	-0.029157	-2.737056	0.579291
4	1-D	-0.265366	-3.390466	-0.275730
5	1-D	0.921516	-3.082948	1.003143
6	1-D	-0.795492	-2.905533	1.351231
7	14	-0.062013	2.708511	0.029628
8	8	1.146776	3.207317	1.089025
9	1	1.659811	3.964370	0.781337
10	8	-0.110042	3.988636	-1.058961
11	1	-0.342146	3.757654	-1.965637
12	8	-1.465886	2.685481	0.954824
13	1	-1.574850	3.422007	1.568457
14	15	2.361501	-0.650926	-0.053163
15	15	-2.311977	-0.708285	-0.071846
16	6	3.350948	-2.207832	0.037493
17	1	4.420185	-1.988426	-0.079988
18	1	3.198471	-2.702309	1.003373
19	1	3.037865	-2.898750	-0.753795
20	6	2.978658	0.148460	-1.592620
21	1	2.415537	1.075968	-1.736303
22	1	4.055262	0.355343	-1.531966
23	1	2.783731	-0.508542	-2.448597
24	6	3.070681	0.369619	1.306970
25	1	4.156556	0.484612	1.185886
26	1	2.588035	1.354112	1.313008
27	1	2.867603	-0.118282	2.267979
28	6	-3.153454	-2.266757	-0.595558
29	1	-2.858759	-3.095993	0.056115
30	1	-4.244404	-2.150355	-0.556684
31	1	-2.857338	-2.516640	-1.621211
32	6	-3.046690	0.551284	-1.194638
33	1	-4.138894	0.442580	-1.229883
34	1	-2.789476	1.546208	-0.820257
35	1	-2.633433	0.434702	-2.202504
36	6	-3.078406	-0.325547	1.559359
37	1	-2.715535	0.658901	1.874848
38	1	-4.174497	-0.311628	1.490584
39	1	-2.771820	-1.073787	2.299480

Table S 6. Calculated sum of electronic and thermal free energies for the optimized structures **A-1**, **A-2**, **A-1-CD$_3$**, and **A-2-CD$_3$**

Optimized structure	Gibbs Free Energy (Hartree/ particle)
**A-1**	-1673.48
**A-2**	-1673.49
**A-1-CD$_3$**	-1673.49
**A-2-CD$_3$**	-1673.50

Table S 7. Selected, calculated harmonic frequencies (cm^{-1}) of all geometry-optimized structures, and their respective scaled values (0.961, in cm^{-1})

Calc. harmonic frequency **A-1**	Scaled **A-1**	Calc. harmonic frequency **A-2**	Scaled **A-2**	Calc. harmonic frequency **A-1-CD$_3$**	Scaled **A-1-CD$_3$**	Calc. harmonic frequency **A-2-CD$_3$**	Scaled **A-2-CD$_3$**
1464.88	1407.75	1464.55	1407.43	1464.91	1407.78	1464.69	1407.56
1466.93	1409.72	1466.80	1409.60	1468.00	1410.75	1467.30	1410.07
1470.11	1412.77	1476.69	1419.10	1478.12	1420.47	1479.81	1422.10
1479.08	1421.39	1478.84	1421.17	1479.79	1422.08	1480.54	1422.80
1480.61	1422.86	1480.64	1422.89	1481.79	1424.00	1481.43	1423.65
1482.05	1424.25	1482.09	1424.28	1482.21	1424.41	1482.44	1424.63
1482.31	1424.50	1483.36	1425.51	1485.74	1427.80	1484.59	1426.69
1483.45	1425.59	1485.56	1427.62	1486.83	1428.84	1485.92	1427.97
1489.65	1431.55	1491.44	1433.27	1491.45	1433.28	1492.71	1434.49
1490.92	1432.78	1494.62	1436.33	1496.09	1437.74	1495.43	1437.10
1496.56	1438.19	1497.34	1438.95	1503.03	1444.41	1505.23	1446.53
1503.08	1444.46	1506.26	1447.51	1512.17	1453.20	1510.65	1451.73
1506.51	1447.76	1508.84	1449.99	2166.24	2081.76	2154.80	2070.77
1512.24	1453.27	1512.89	1453.89	2297.79	2208.18	2268.90	2180.41
3022.34	2904.47	3001.46	2884.40	2320.54	2230.04	2294.78	2205.28
3027.57	2909.49	3032.27	2914.01	3027.55	2909.48	3032.27	2914.01
3037.46	2919.00	3038.56	2920.06	3037.46	2918.99	3038.56	2920.05
3042.05	2923.41	3041.78	2923.15	3042.07	2923.43	3041.78	2923.15
3045.76	2926.98	3044.42	2925.68	3045.77	2926.99	3044.41	2925.68
3046.52	2927.70	3044.85	2926.10	3046.52	2927.71	3044.95	2926.19
3052.47	2933.42	3046.26	2927.45	3052.37	2933.33	3046.23	2927.43
3099.78	2978.89	3061.83	2942.42	3113.38	2991.96	3119.63	2997.97
3113.35	2991.93	3099.41	2978.54	3115.39	2993.89	3124.32	3002.48
3115.39	2993.89	3119.64	2997.97	3123.00	3001.20	3125.64	3003.74
3122.98	3001.18	3124.33	3002.48	3129.07	3007.04	3126.78	3004.84
3129.07	3007.04	3125.67	3003.76	3129.99	3007.92	3127.30	3005.34
3129.12	3007.09	3126.80	3004.86	3131.17	3009.05	3132.30	3010.14
3130.13	3008.06	3127.43	3005.46	3134.22	3011.99	3138.91	3016.49
3131.20	3009.09	3132.29	3010.14	3137.07	3014.72	3141.73	3019.20
3134.34	3012.10	3138.91	3016.49	3148.83	3026.03	3146.79	3024.06
3137.33	3014.98	3141.87	3019.34	3153.69	3030.69	3150.13	3027.27
3148.84	3026.03	3146.83	3024.11	3155.15	3032.10	3157.61	3034.46
3153.72	3030.73	3150.13	3027.28	3165.87	3042.40	3167.16	3043.64
3155.37	3032.31	3157.61	3034.46				
3165.79	3042.32	3167.16	3043.64				

Elemental analysis of $PtMe(PMe_3)_2/SiO_2^{700}$ PtMe(dmpe) /SiO_2^{700}, and PtMe(cod) /SiO_2^{700}

For the synthesis of the platinum surface complexes, the following masses of precursors and SiO_2^{700} were used in grafting, which correspond to the given required contents (wt%) in Table S9-S11. For $PtMe(PMe_3)_2/SiO_2^{700}$, the experiments are ordered from lower to higher expected loading (equally to the order in Table S 9).

Table S 8. Weighed-in masses of dimethyl platinum precursors and SiO_2^{700}, which were used in the grafting reactions to prepare silica-supported platinum surface complexes

Compound	$m_{Precursor}$ (mg)	$m_{Silica\text{-}700}$ (mg)
$PtMe(PMe_3)_2/SiO_2^{700}$	10.2	500.9
	12.2	500.2
	15.3	503.3
	25.0	500.0
$Pt(CD_3)(PMe_3)_2/SiO_2^{700}$	15.1	500.6
PtMe(dmpe) /SiO_2^{700}	20.1	500.7
PtMe(cod) /SiO_2^{700}	38.6	1000.2

Table S 9. Results of the elemental analysis of **A,** platinum and phosphorous loading ±0.5 wt%, C and H content ±0.3 wt%

$PtMe(PMe_3)_2/SiO_2^{700}$ [a]	Pt	C	H	P
**Required/ found content (wt%)**	1.05/ 0.93	0.45/ 0.76	0.11/ 0.1	0.33/ 0.3
	1.26/ 1.23	0.54/ 0.67	0.14/ 0.16	0.40/ 0.4
	1.56/ 1.5	0.67/ 0.85	0.17/ 0.23	0.49/ 0.5
	2.58/ 2.2	1.11/ 1.30	0.28/ 0.20	0.82/ 0.7
**Expected/ found molar ratio**	1.0/ 1.0 [b]	7.0/ 13.3	21.0/ 20.8	2.0/ 2.0
	1.0/ 1.0	7.0/ 8.8	21.0/ 25.1	2.0/ 2.0
	1.0/ 1.0	7.0/ 9.2	21.0/ 29.6	2.0/ 2.1
	1.0/ 1.0	7.0/ 9.6	21.0/ 17.6	2.0/ 2.0

[a] $Pt(CD_3)(PMe_3)_2/SiO_2^{700}$: Pt content expected/found (wt%) 1.5/ 1.0; C, H, and P were not determined

[b] set to 1.0 for all

Table S 10. Results of the elemental analysis of PtMe(dmpe) /SiO_2^{700} (**B**), error of platinum and phosphorus content ±0.5 wt%; error of C, and H content ±0.3 wt%

**PtMe(dmpe) /SiO_2^{700}**	**Pt**	**C**	**H**	**P**
Required/ found content (wt%)	2.09/ 2.05	0.9/ 1.26	0.21/ 0.24	0.74/ 0.69
Expected/ found molar ratio	1.0/ 1.0 [a]	7.0/ 10.0	19.0/ 22.6	2.0/ 1.8
[a] set to 1.0				

Table S 11. Results of the elemental analysis of PtMe(cod) /SiO_2^{700} (**C**), error of platinum content ±0.5 wt%; error of C, and H content ±0.3 wt%

**PtMe(cod) /SiO_2^{700}**	**Pt**	**C**	**H**
Required/ found content (wt%)	2.26/ 1.71	1.25/ 1.29	0.18/ 0.20
Expected/ found molar ratio	1.0/ 1.0 [a]	9.0/ 12.3	15.0/ 24.5
[a] set to 1.0			

The molar amount of supported platinum was calculated from the Pt loading determined in elemental analysis. For calculating the coverage percentage of isolated SiOH groups, it was divided by the expected molar amounts of the isolated SiOH groups on 500 mg SiO_2^{700} (0.23 mmol g^{-1}),[12] i.e. 0.115 mmol.

Table S 12. Calculations of the coverage of isolated SiOH groups with the platinum surface complexes

Compound	Obtained Pt loading (wt%) in elemental analysis	Molar ratio [b] n_{Pt}(mmol)/n_{SiOH}(mmol)	Coverage of isolated SiOH groups (%)
$PtMe(PMe_3)_2/SiO_2^{700}$	0.93	0.0239/0.115	20.8
	1.23	0.0315/0.115	27.4
	1.5	0.0387/0.115	33.7
	2.2	0.0564/0.115	49.0
$Pt(CD_3)(PMe_3)_2/SiO_2^{700}$	1.0	0.0257/0.115	22.3
PtMe(dmpe) /SiO_2^{700}	2.05	0.0525/0.115	45.7
PtMe(cod) /SiO_2^{700}	1.71	0.0877/0.23 [a]	38.1
[a] grafting with 1000 mg SiO_2^{700}<br>[b] calculated molar amount of platinum on 500 mg SiO_2^{700} (or 1000 mg, in the case of [a]) from obtained Pt loading			

EXAFS Analysis of $PtMe(PMe_3)_2/SiO_2^{700}$ and $PtMe(cod)/SiO_2^{700}$

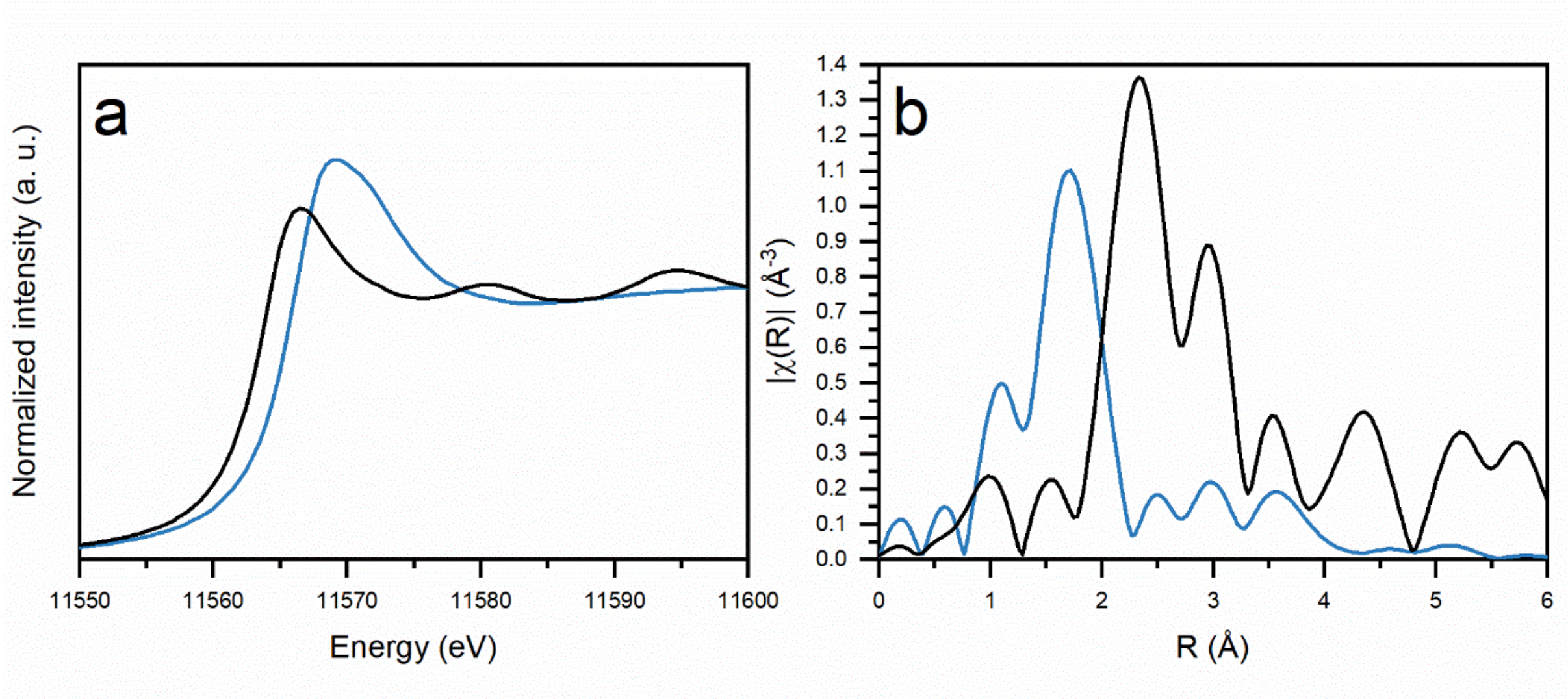

Figure S 11. a) Normalized X-ray absorption spectra at the Pt L_3-edge and b) the Fourier-transformations thereof for $PtMe(PMe_3)_2/SiO_2^{700}$ (**A**, blue), and a platinum foil (black).

For the analysis of EXAFS data of $PtMe(PMe_3)_2/SiO_2^{700}$, the structural data of *cis*-$[PtMe(OPh)(PMe_3)_2]$ (CCDC 1257257)[13] were used as the starting values for the fitting process. The complex describes the close surroundings of the Pt(II) scattering center in **A** appropriately (Table S 13, Figure S 12). In contrast, platinum metal foil was not appropriate as a fit.

Table S 13. Coordination numbers and experimental interatomic distances for $PtMe(PMe_3)_2/SiO_2^{700}$ by EXAFS analysis, compared to expected distances for the fit cis-$[PtMe(OPh)(PMe_3)_2]$

Scatter	Number of backscatterers (N_{BS})	$R_{expected}$ (Å)	$R_{experimental}$ (Å)	σ^2
Pt-C/O	2	2.08	2.08 ± 0.03	0.0034 ± 0.0009
Pt-P	2	2.36	2.27 ± 0.02	
S_0^2 = 0.7460; ΔE_0 = 4.16 ± 1.84 eV; X^2_{red} = 121.57; R^h = 0.041; N(path) = 2; N(par) = 4; k-range: 2 – 11; R-range: 1 – 3.				

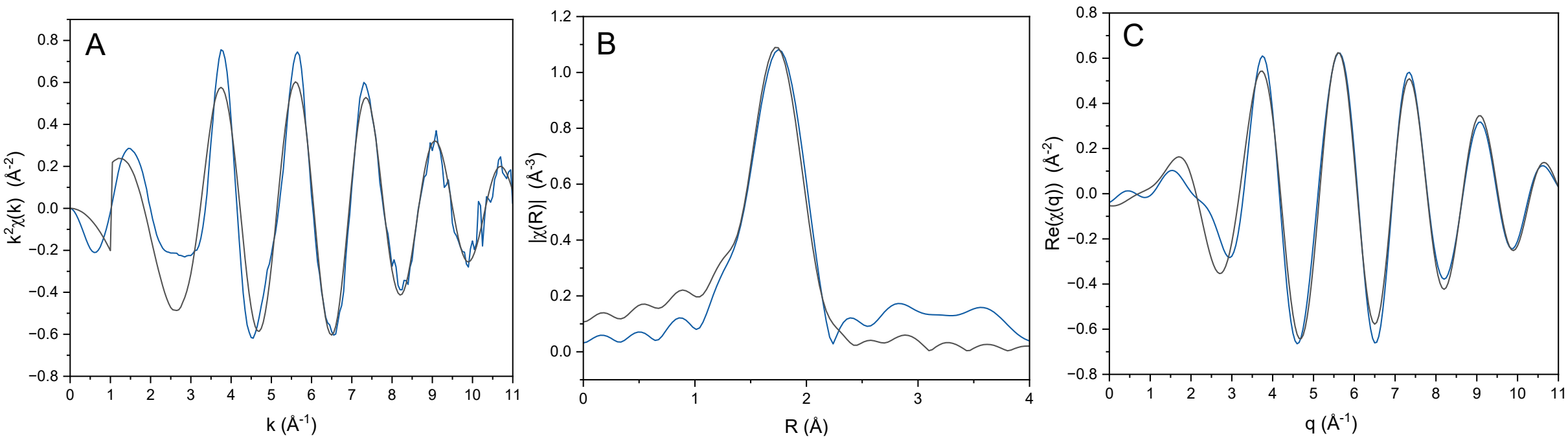

Figure S 12. Comparison of the Fourier-transformed EXAFS spectra for the surface complex $PtMe(PMe_3)_2/SiO_2^{700}$ (blue) and the best fit results based on the model complex cis-$[PtMe(OPh)(PMe_3)_2]$ (grey). EXAFS spectra (A) Fourier-transformed EXAFS spectra (B) and the back transformation thereof (C).

For the analysis of EXAFS data of PtMe(cod)/SiO_2^{700}, the structural data of [(cod)PtMe(OH)] (CCDC 133298)[14] was used as the starting values for the fitting process. The complex describes the close surroundings of the Pt(II) scattering center in **C** appropriately (Table S 14). The reliability of the model decreased substantially when extending the fit beyond the first coordination shell. Platinum metal foil was not appropriate as a fit.

Table S 14. Coordination numbers and experimental interatomic distances for PtMe(cod)/SiO_2^{700} by EXAFS analysis, compared to expected distances for the fit [(cod)PtMe(OH)]

Scatter	Number of backscatterers (N_{BS})	$R_{expected}$ (Å)	$R_{experimental}$ (Å)	σ^2
Pt-C	6.57 ± 1.16	2.13	2.10 ± 0.02	0.0062 ± 0.0028
S_0^2 = 0.7463; ΔE_0 = 9.37 ± 1.81 eV; X^2_{red} = 150.89; R^h = 0.062; N(path) = 1; N(par) = 4; k-range: 2 – 11; R-range: 1 – 3.				

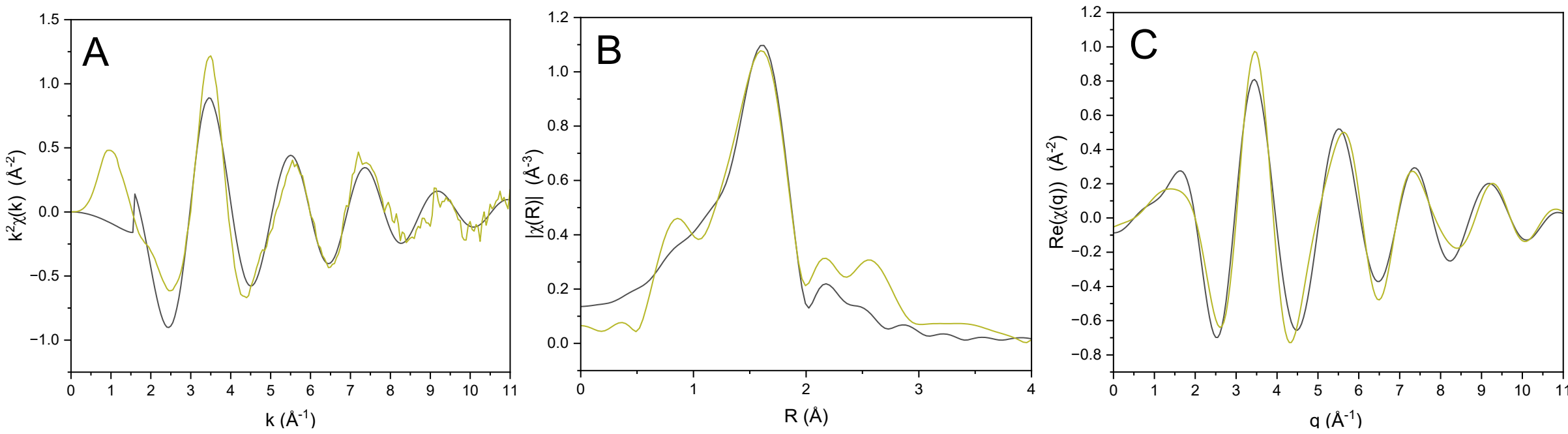

Figure S 13. Comparison of the Fourier-transformed EXAFS spectra for the surface complex PtMe(cod)/SiO_2^{700} (light green) and the best fit results based on the model complex [(cod)PtMe(OH)] (grey). EXAFS spectra (A) Fourier-transformed EXAFS spectra (B) and the back transformation thereof (C).

Solid-state NMR spectra and signals of $PtMe(PMe_3)_2/SiO_2$, PtMe(dmpe)/SiO_2, PtMe(cod)/SiO_2^{700}

PtMe(PMe_3)$_2$/SiO_2^{700} (**A**): **^{1}H MAS NMR** (300 MHz, 4 mm rotor, 15 kHz) δ (ppm) = 1.9 (Pt-P<u>Me</u>), 1.5 (Pt-P<u>Me</u>), 0.2 (broad, Pt-<u>Me</u>). **^{31}P HPDEC NMR**: see Table 1.

PtMe(dmpe)/SiO_2^{700} (**B**): **^{1}H MAS NMR** (300 MHz, 4 mm rotor, 13 kHz): δ(ppm) = 1.9 (Pt-P<u>Me</u>, Pt-PC<u>H$_2$</u>), 0.1 (Pt-<u>Me</u>). **^{31}P HPDEC NMR** (121 MHz, 4 mm rotor, 13 kHz): δ (ppm) = 10 ($^1J_{PtP}$ = 4162 Hz), 35 ($^1J_{PtP}$ = 1504 Hz);

PtMe(cod)/SiO_2^{700} (**C**): **^{1}H MAS NMR** (300 MHz, 4 mm rotor, 15 kHz): δ (ppm) = 3.6 (=C<u>H</u>), 2.0 (-C<u>H$_2$</u>-), 1.2 (Pt-<u>Me</u>)

Additionally, ≡SiO-<u>H</u> contribute to ^{1}H MAS NMR signals at δ = 1.4 ppm.

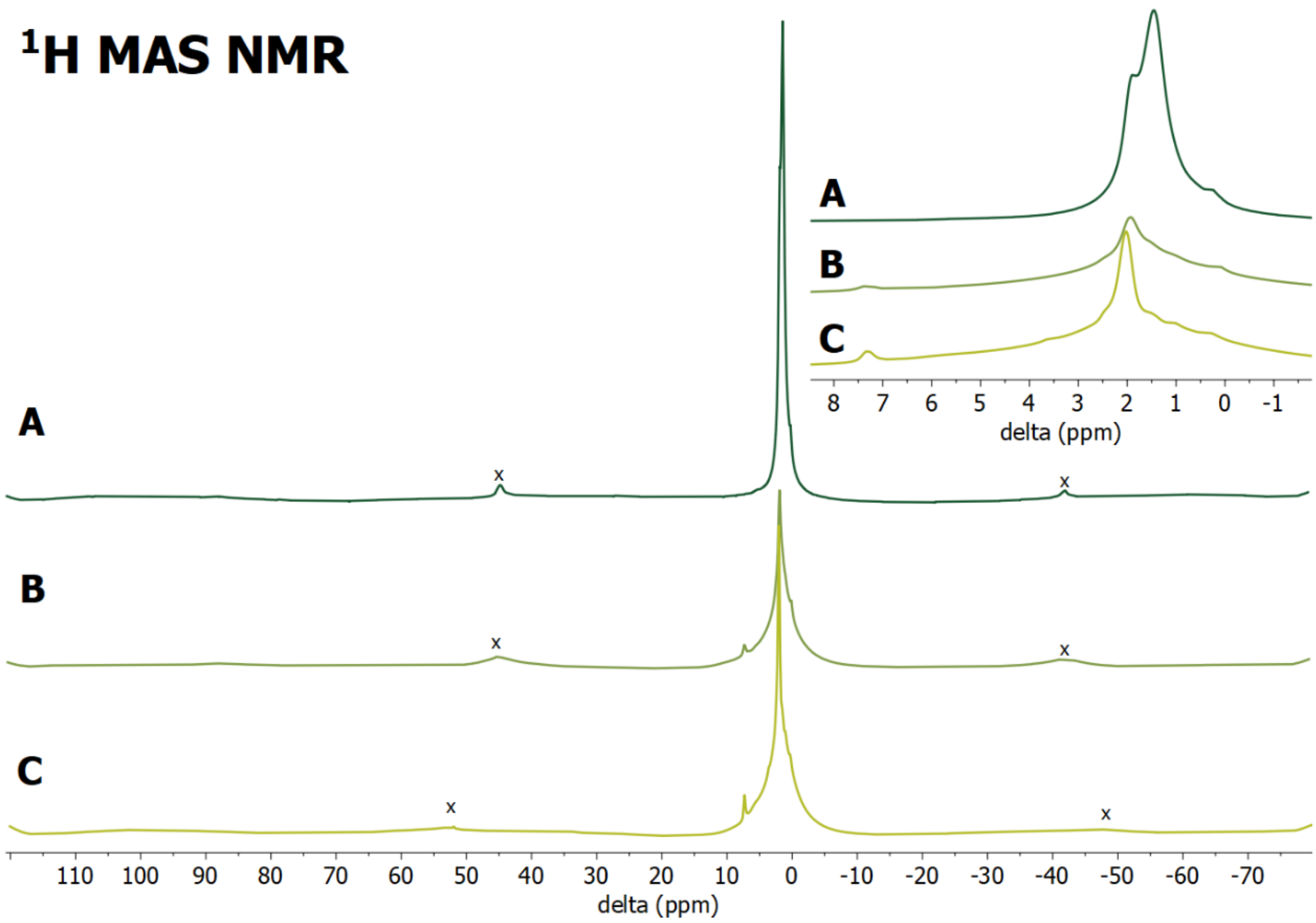

Figure S 14. Solid-state ^{1}H NMR spectra (300 MHz) of densely packed rotors (4 mm) with PtMe(PMe_3)$_2$/SiO_2 (**A**, dark green, MAS: 15 kHz), PtMe(dmpe)/SiO_2 (**B**, green, MAS: 13 kHz), and PtMe(cod) /SiO_2^{700} (**C**, light green, MAS: 15 kHz). The region between -1 and 8 ppm is enlarged in the upper right. Spinning side bands are marked with x.

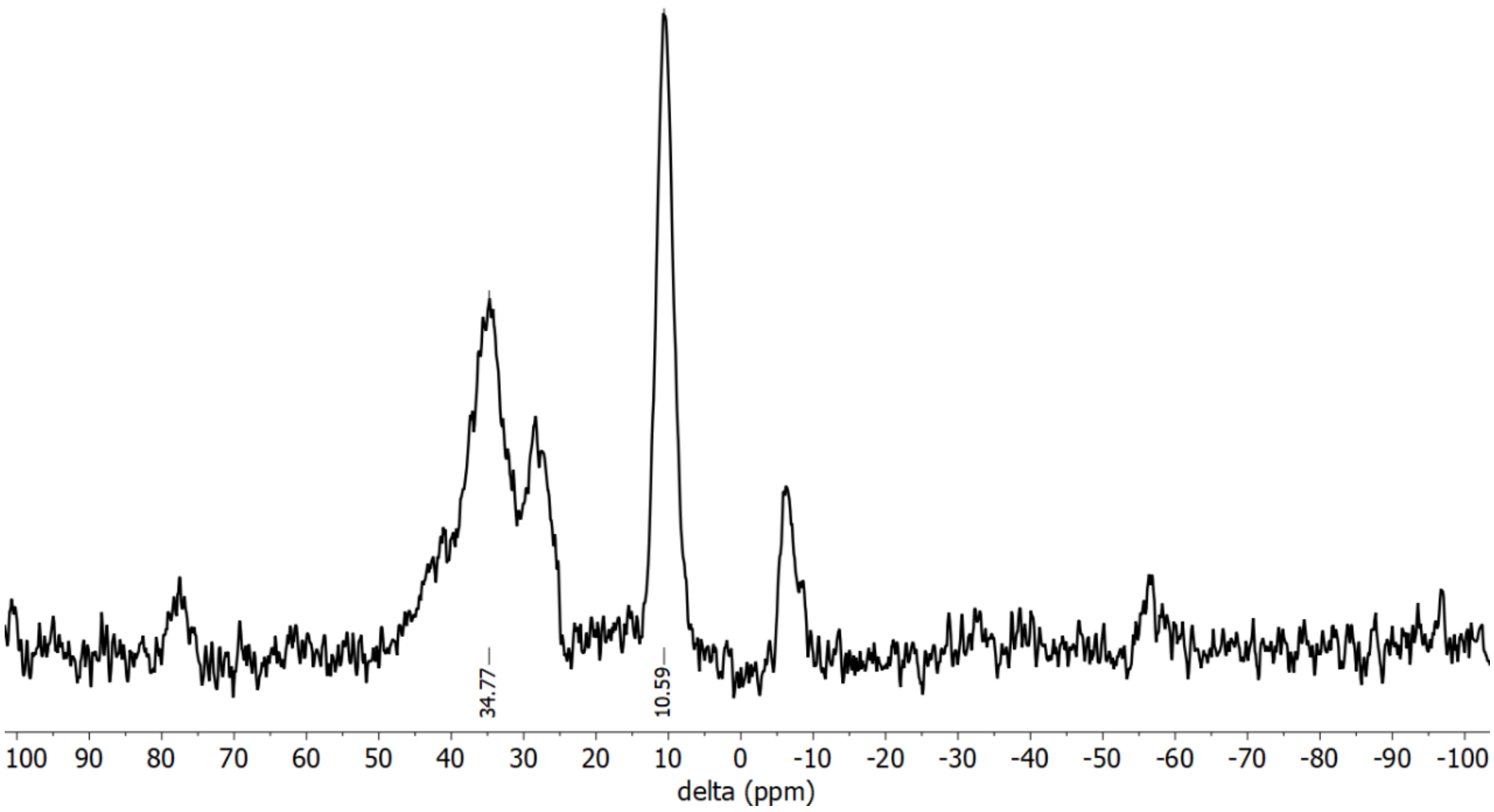

Figure S 15. Solid-state $^{31}P\{^{1}H\}$ spectrum (121 MHz) of PtMe(dmpe) /SiO_2^{700} in a 4 mm rotor with magic angle spinning (13 kHz).

IR spectra of CO chemisorption bands, characterizing the surface complexes and pre-treated materials

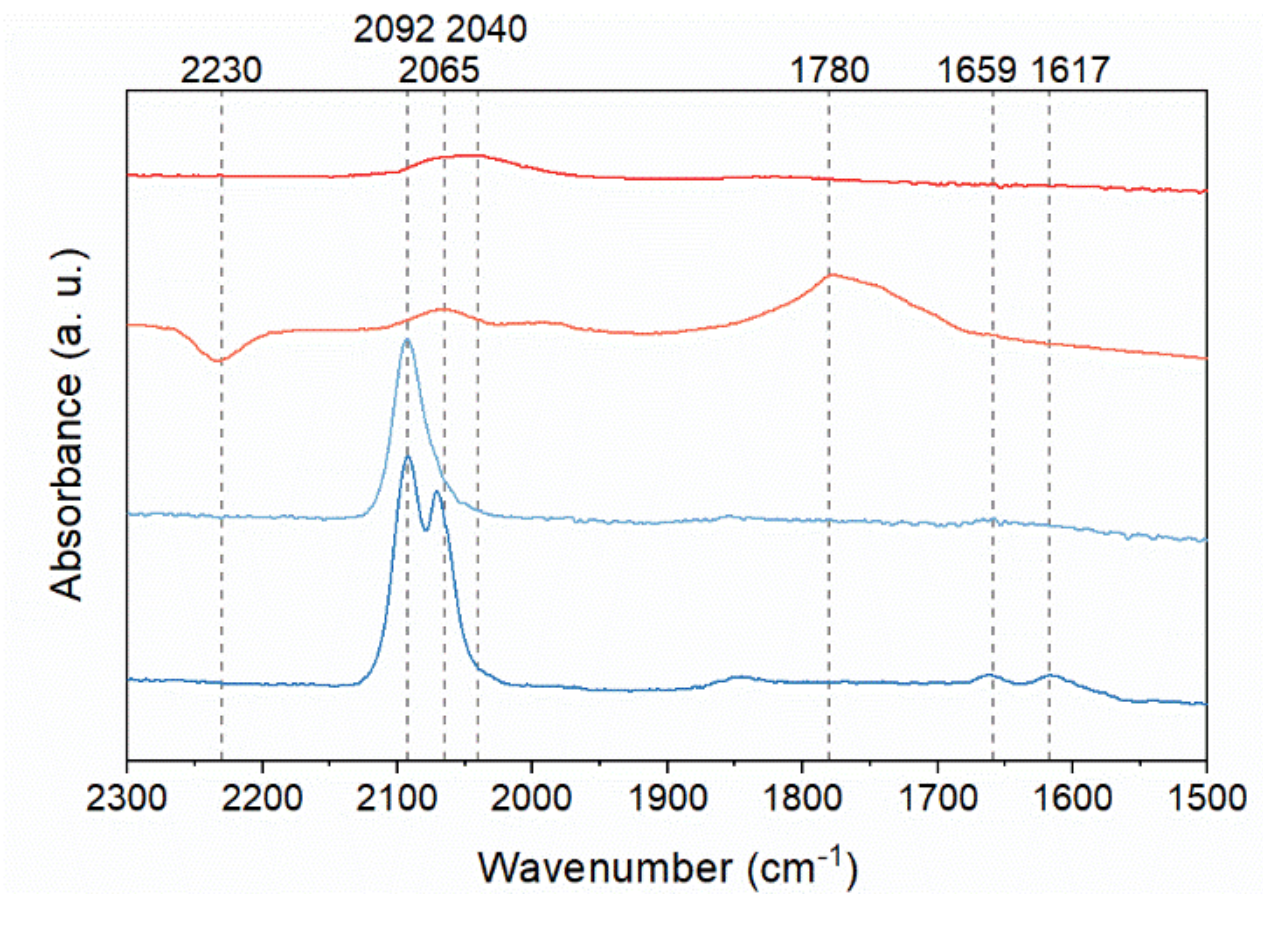

Figure S 16. FT-IR spectra of self-supporting discs after CO chemisorption for 30 min (1% CO in helium, 20 mLmin^{-1}), and a helium purge (5 min, 20 mLmin^{-1}) at room temperature: **A-CO** (dark blue), **A-He-200_CO** (light blue), **A-H_2-150_CO** (orange), and **A-H_2-350_CO** (red); a spectrum of the disc was subtracted prior to the submission of the reactive gas into the IR cell.

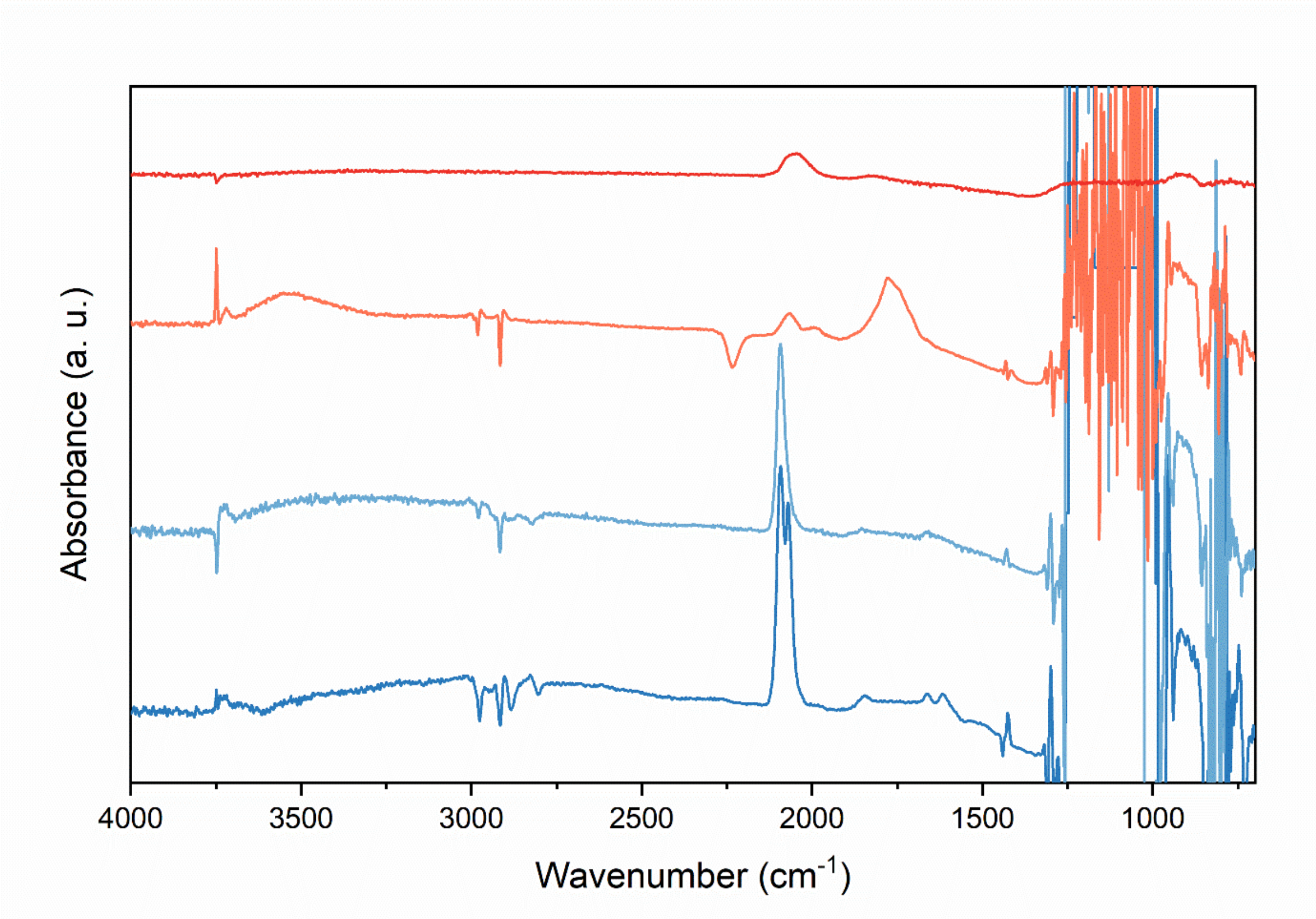

Figure S 17. Full FT-IR spectra after CO chemisorption for 30 min at room temperature at the materials **A** (dark blue), **A-He-200** (light blue), **A-H$_2$-150** (orange), **A-H$_2$-350** (red) with 1% CO in helium in flow (20 mLmin^{-1}), spectra measured after flushing the IR cell with helium (5 min, 20 mLmin^{-1}).

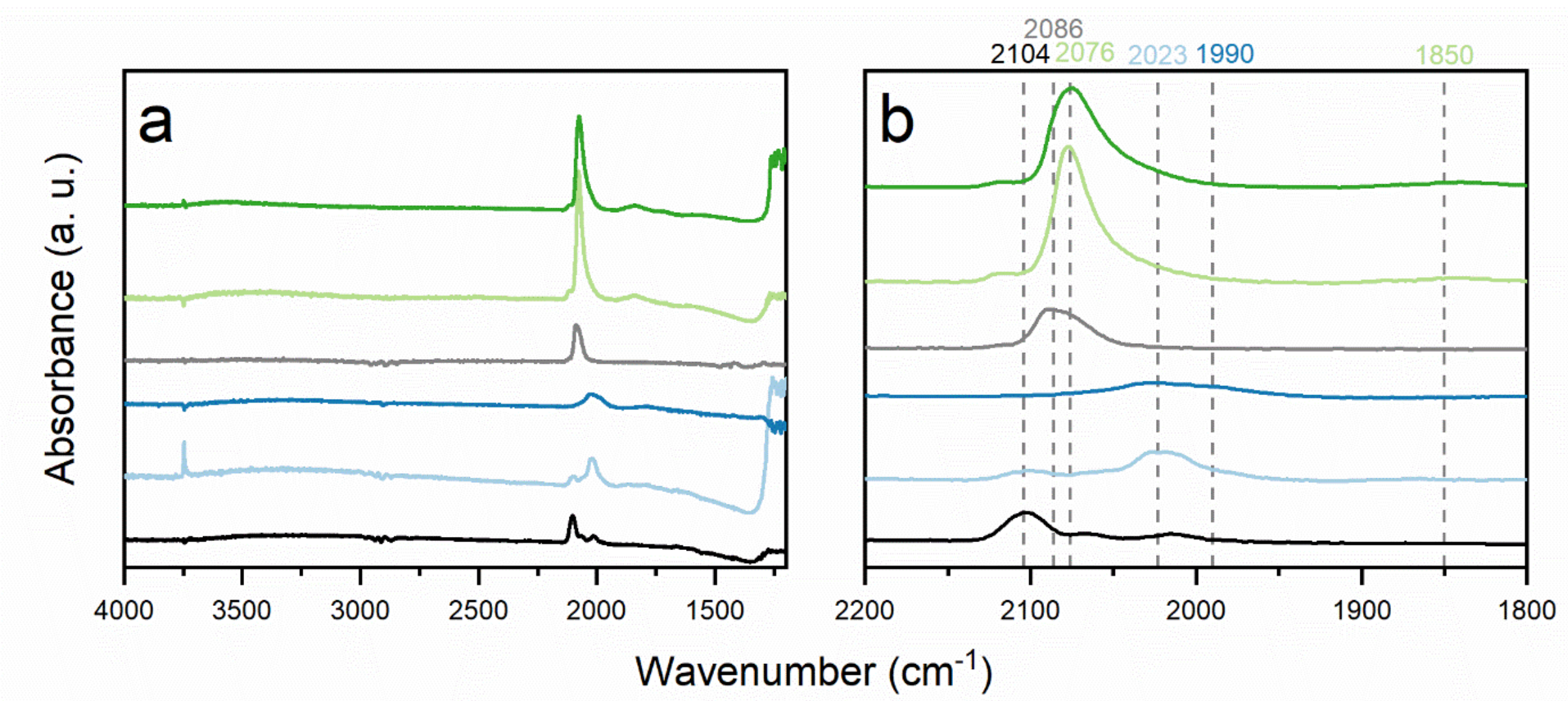

Figure S 18. a) FT-IR spectra after CO chemisorption for 30 min at room temperature at the materials **B** (black), **B-H$_2$-150** (light blue), **B-H$_2$-180** (dark blue), **C** (grey), **C-H$_2$-150** (light green), and **C-H$_2$-180** (dark green), with 1% CO in helium in flow (20 mLmin^{-1}), spectra measured after flushing the IR cell with helium (5 min, 20 mLmin^{-1}), and b) spectra enlarged in the region of terminal and bridged CO vibrations.

Table S 15. Summary of discussed compounds, pre-treatments, CO stretching vibrations, and chemical shifts in ^{31}P spectra with ^{195}Pt-^{31}P coupling constants

Entry	Species (surface or solution)	Pre-treatment (gas, temperature)	υ_{CO} (cm^{-1})	δ_P (ppm)	Coupling constant $^1J_{Pt-P}$ (Hz)	Reference
**1**	≡SiO-PtMe(PMe_3)$_2$ (**A**)	None				This work
	(cis)			-35	4208	
				-9	1628	
	(*trans*)			-11	2870	
**2**[a]	*cis*-[PtMe(MeOH)(PMe_3)$_2$]$^+$			-29.6	4367	[15]
				-4.0	1780	
**3**[a]	*trans*-[PtMe(MeOH)(PMe_3)$_2$]$^+$			-6.4	2789	[15]
**4**[a, b]	*cis*-[PtMe(ac)(PMe_3)$_2$]$^+$			-25.2	4140	[16]
				-13.2	1687	
**5**[a, b]	*trans*-[PtMe(ac)(PMe_3)$_2$]$^+$			-6.7	2717	[16]
**6**	**A-CO**	1% CO, r. t.				This work
	≡SiO$^-$ [PtMeCO(PMe_3)$_2$]$^+$		2092	-11	2960	
	≡SiO-Pt(Me)(CO)(PMe_3)		2071, 2078 (shoulder)	-18 (?)		
**7**[a]	*trans*-[PtMeCO(PMe_3)$_2$]$^+$		2092	-13.6	2299	[16]
**8**	[Pt(Me)(I)(PRR'$_2$)(CO)] R, R': -Ph, -(*o*-MeC$_6$H$_4$)		2078			[17]
**9**	**A-He-200** ≡SiO-PtMe(PMe_3)$_2$ (trans)	Helium, 200 °C		-12	2884	This work
**10**	**A-He-200_CO**		2092			This work

**11**[c]	**A-H_2-150**	10% H_2, 150 °C				This work
	≡SiO-Pt(H)$(PMe_3)_2$ (trans)			-12	2747	
	$Pt_x(PMe_3)_y/SiO_2$					
**12**[b, d]	*trans*-$[Pt(H)(PMe_3)_2(ac)]PF_6$			-10.5	2540	[18]
**13**	**A-H_2-150_CO**					This work
	≡SiO-Pt(H)$(PMe_3)_2$ (trans)		Decomp.			
	$Pt_x(PMe_3)_{y-n}(CO)_n/SiO_2$		2065, 1990, 1780			
**14**	$Pt(CO)_2(PPh_3)_2$		1983, 1948			[19]
**15**	$Pt_3(CO)_3(PPh_2Bz)_3$		1784			[19]
**16**	$Pt_4(CO)_5(PEt_3)_4$		1980, 1875, 1845, 1785, 1755			[20]
**17**	**A-H_2-350**	10% H_2, 350°C				This work
	Pt_xP_y/SiO_2					
**18**	**A-H_2-350_CO**					This work
	$Pt_xP_y(CO)_n/SiO_2$		2040			

a BF_4^- as counter ion

b ac: acetone-d_6

c υ_{Pt-H}: 2230 cm^{-1}, δ_H: -22 ppm (Pt-H, $^1J_{Pt-H}$ = 1167 Hz)

d δ_H: -23.7 ppm (Pt-H, $^1J_{Pt-H}$ = 1457 Hz)

Supplementary material: Reactivity of A with carbon monoxide

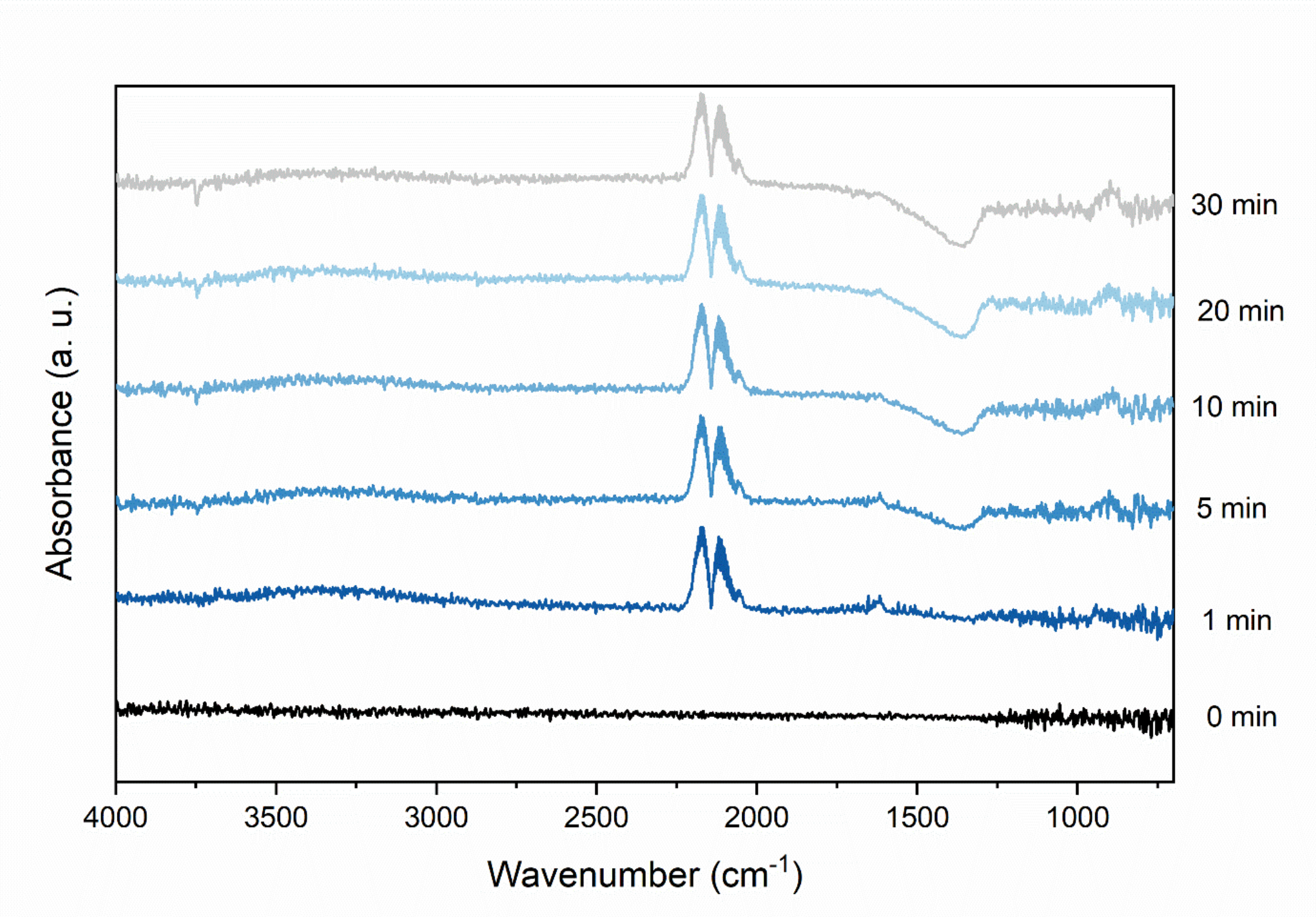

Figure S 19. Time-dependent IR spectra of a CO flow (1% in helium, 20 mLmin^{-1}) passing over a pellet of SiO_2^{700} at room temperature. The spectrum of the pellet was set as the background.

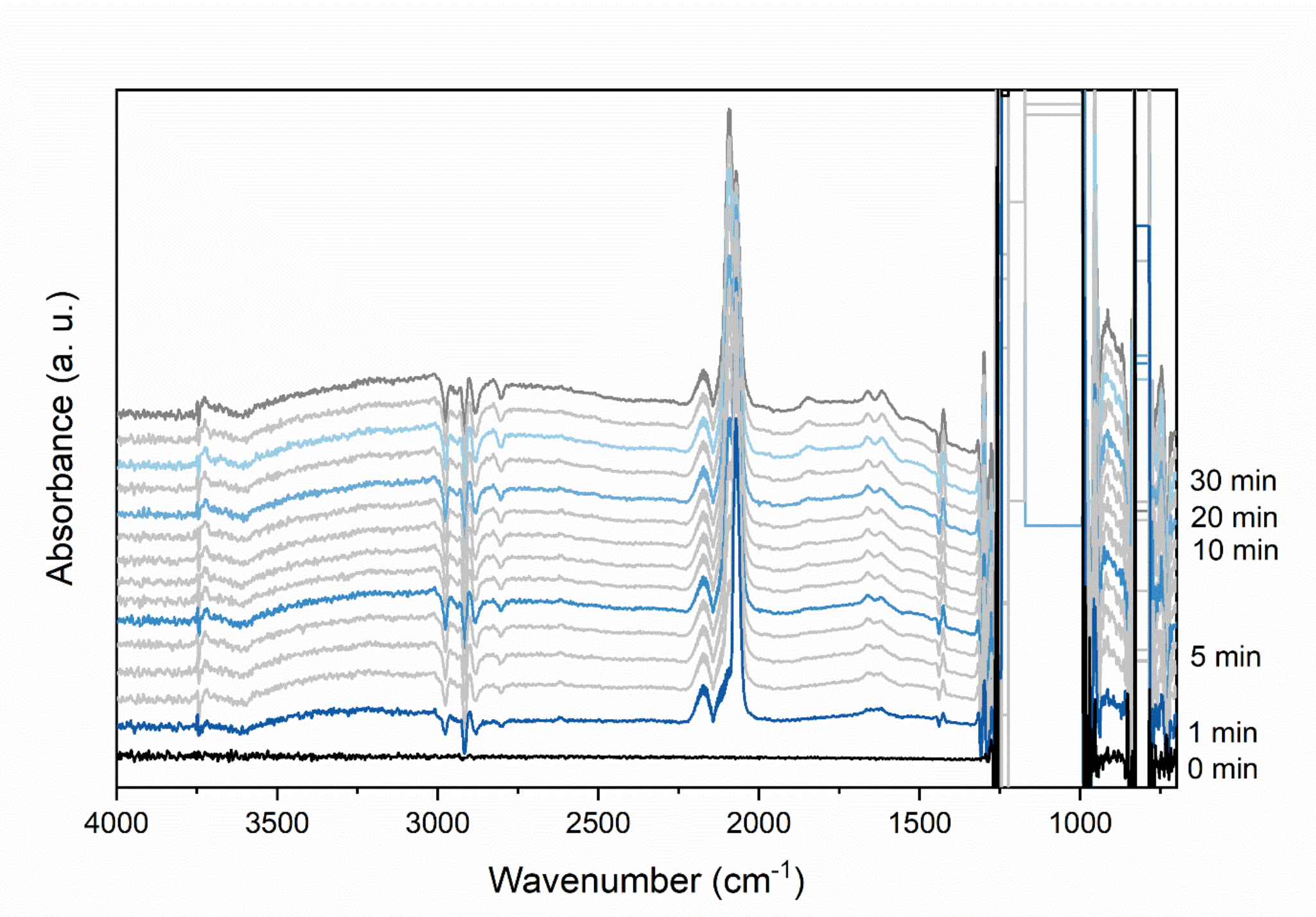

Figure S 20. Time-dependent IR spectra during the CO chemisorption on $PtMe(PMe_3)_2/SiO_2^{700}$ with a gas flow of 1% CO in helium (20 mLmin^{-1}) for 30 min at room temperature; a spectrum was collected every minute in the first 10 minutes, then it was collected every 5 min. These spectra were normalized to the band of gas phase CO at 2171 cm^{-1}, and the normalized intensities of the bands at 2092 cm^{-1} and 2071 cm^{-1} plotted vs time (see Results and Discussion).

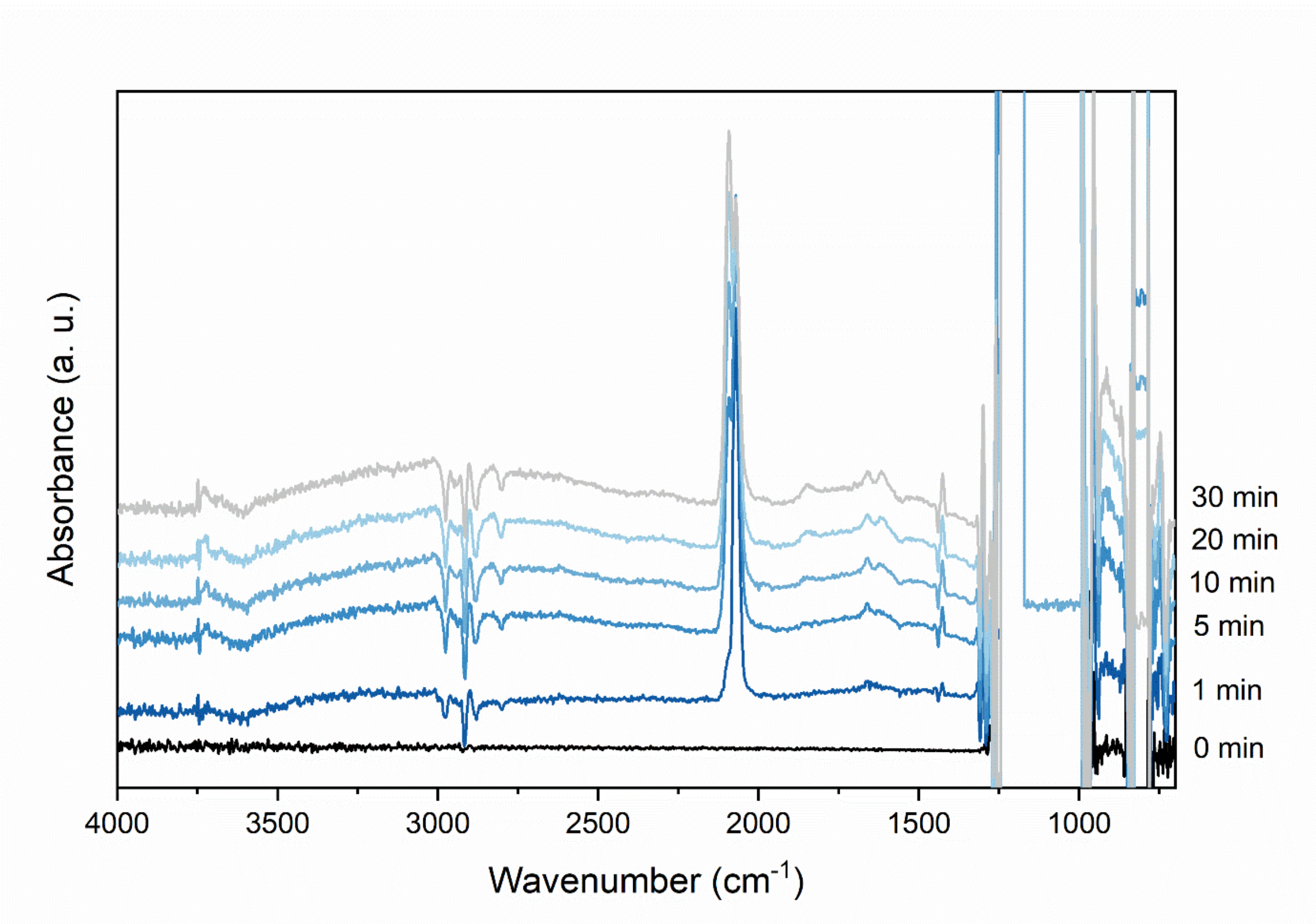

Figure S 21. Time-dependent spectra of the CO chemisorption of $PtMe(PMe_3)_2/SiO_2^{700}$, after subtracting the spectra of CO passing over SiO_2^{700}.

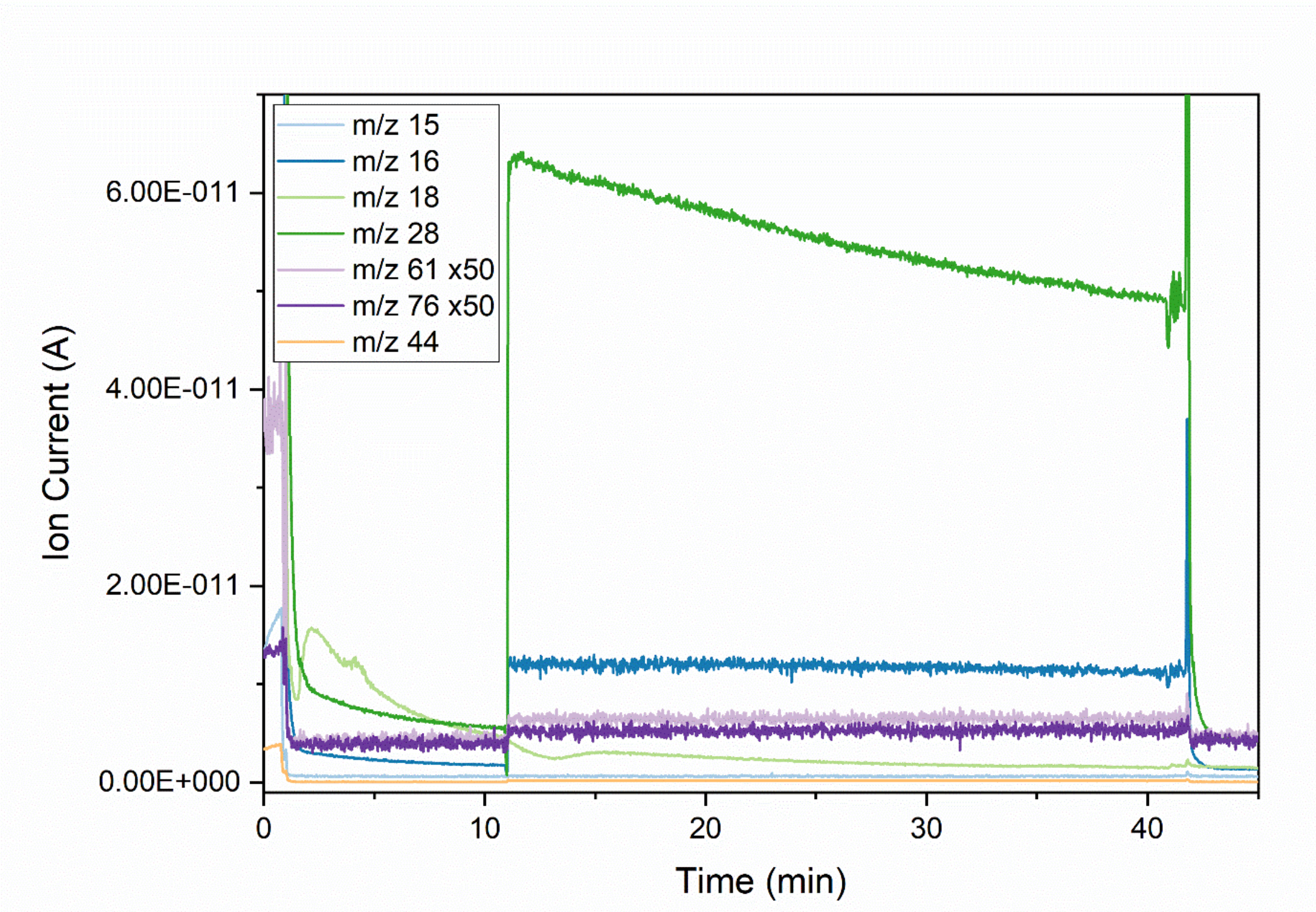

Figure S 22. Gas analysis of the out-stream by mass spectrometry: Helium purge for 11 min (50 mLmin^{-1}), followed by treatment of 100 mg $PtMe(PMe_3)_2/SiO_2^{700}$ under a flow of 1% CO in helium (50 mLmin^{-1}) at room temperature for 30 min; m/z 15 (methyl), m/z (O, fragment of CO), m/z 18 (H_2O), m/z 28 (CO), m/z 61, 76 (multiplied by 50, fragments of PMe_3), and m/z 44 (CO_2).

EXAFS analysis of $PtMe(PMe_3)_2/SiO_2^{700}$, pre-treated in carbon monoxide

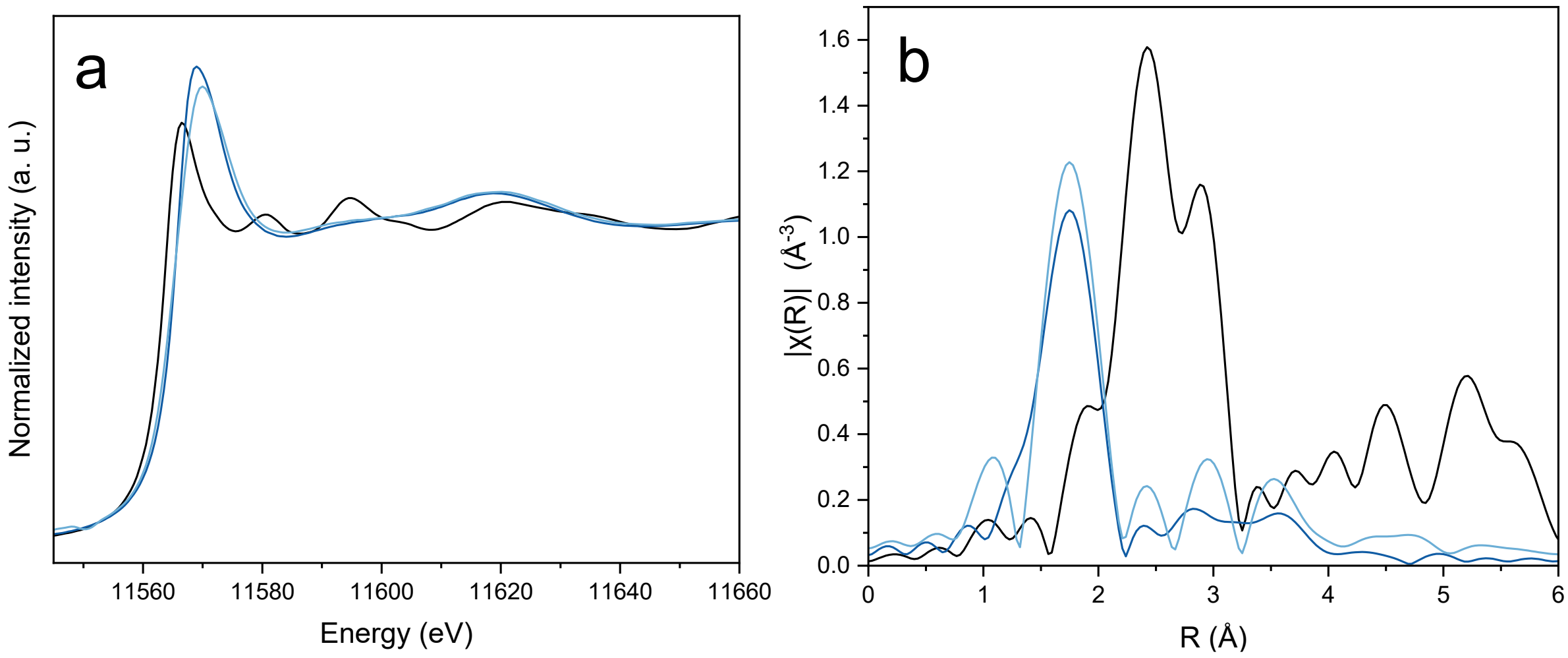

Figure S 23. a) Normalized X-ray absorption spectra at the Pt L_3-edge and b) the Fourier-transformations thereof for $PtMe(PMe_3)_2/SiO_2^{700}$ (**A**, blue), $PtMe(PMe_3)_2/SiO_2^{700}$ pre-treated in 1% CO in helium (50 $mLmin^{-1}$) for 20 min at room temperature (light blue), and a platinum foil (black).

Likewise, a pre-treatment under carbon monoxide (1% CO in helium, 50 $mLmin^{-1}$) at higher temperatures (100 °C or 150 °C for 20 min) did not lead to platinum clusters, since no Pt-Pt contacts are detected in the EXAFS spectra (Figure S 24).

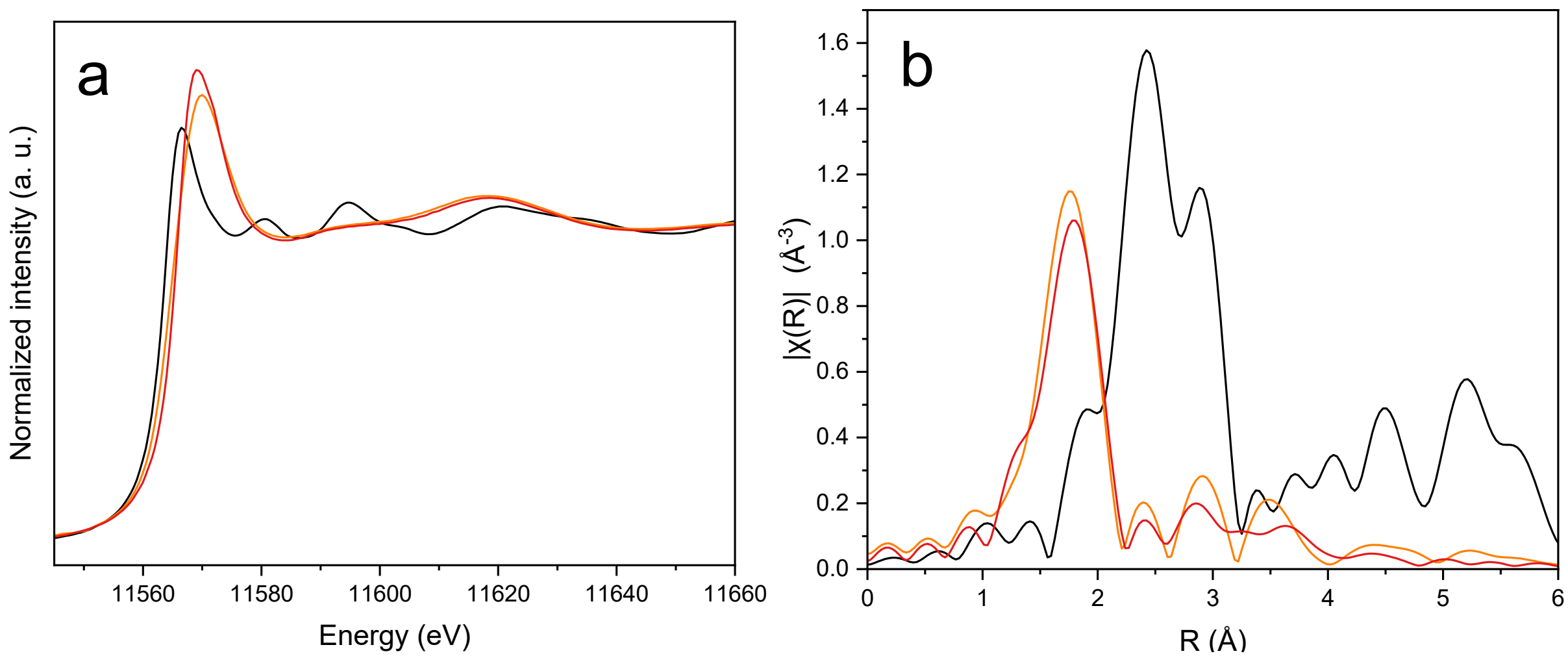

Figure S 24. a) Normalized X-ray absorption spectra at the Pt L_3-edge and b) the Fourier-transformations thereof for $PtMe(PMe_3)_2/SiO_2^{700}$ pre-treated in 1% CO in helium (50 $mLmin^{-1}$) for 20 min at 100 °C (**A-CO-100**, orange) and 150 °C (**A-CO-150**, red), and a platinum foil (black).

Supplementary material: Reactivity of A under inert gas

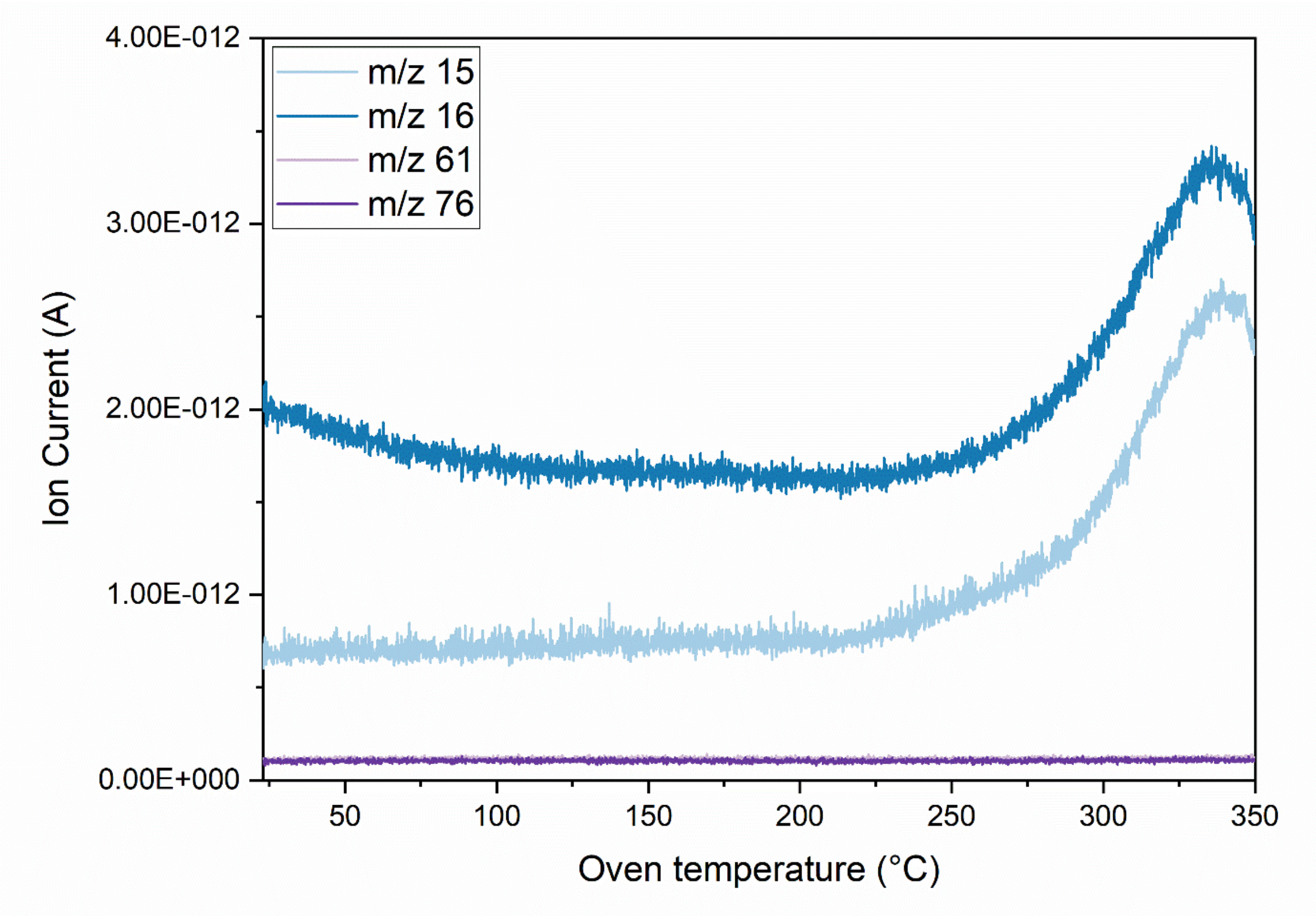

Figure S 25. Gas-analysis of out-stream by mass spectrometry: Heating of 100 mg $PtMe(PMe_3)_2/SiO_2^{700}$ placed in a U-shaped reactor with 5 $Kmin^{-1}$ in a helium flow (50 $mLmin^{-1}$); m/z 15, 16 (methyl, methane), m/z 61, 76 (fragments of PMe_3).

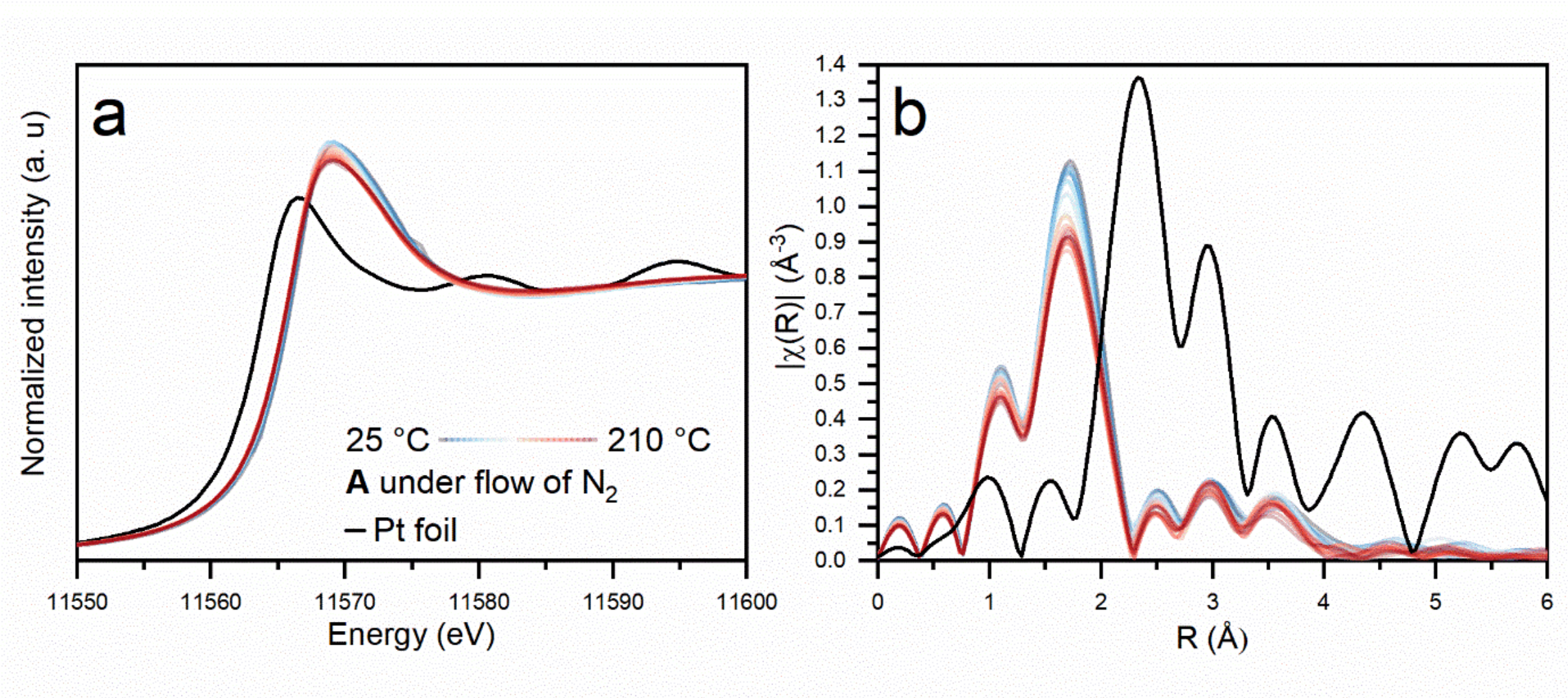

Figure S 26. a) *In situ* X-ray absorption spectra of **A** filled into a quartz glass capillary under a flow of N_2 (20 $mLmin^{-1}$) with 5 $Kmin^{-1}$ heating rate to 210 °C (color) in contrast to a spectrum of a platinum foil (black), and b) Fourier-transformed spectra thereof.

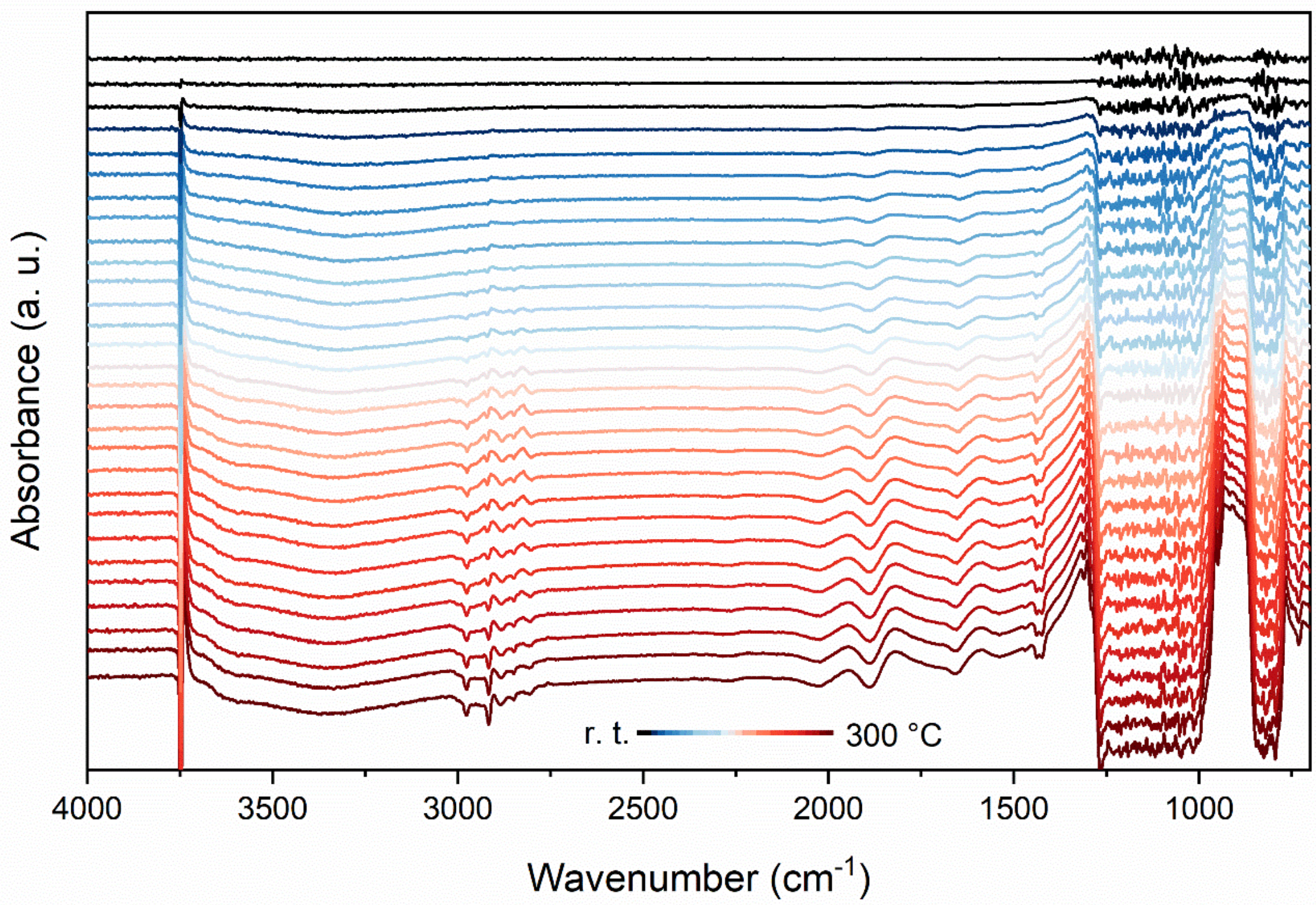

Figure S 27. Temperature-dependent FT-IR spectra of a pellet of **A** during heating with 5 $Kmin^{-1}$ in a flow of helium (20 $mLmin^{-1}$). A spectrum was collected every 10 K. The spectrum of the pellet before heating has been subtracted before.

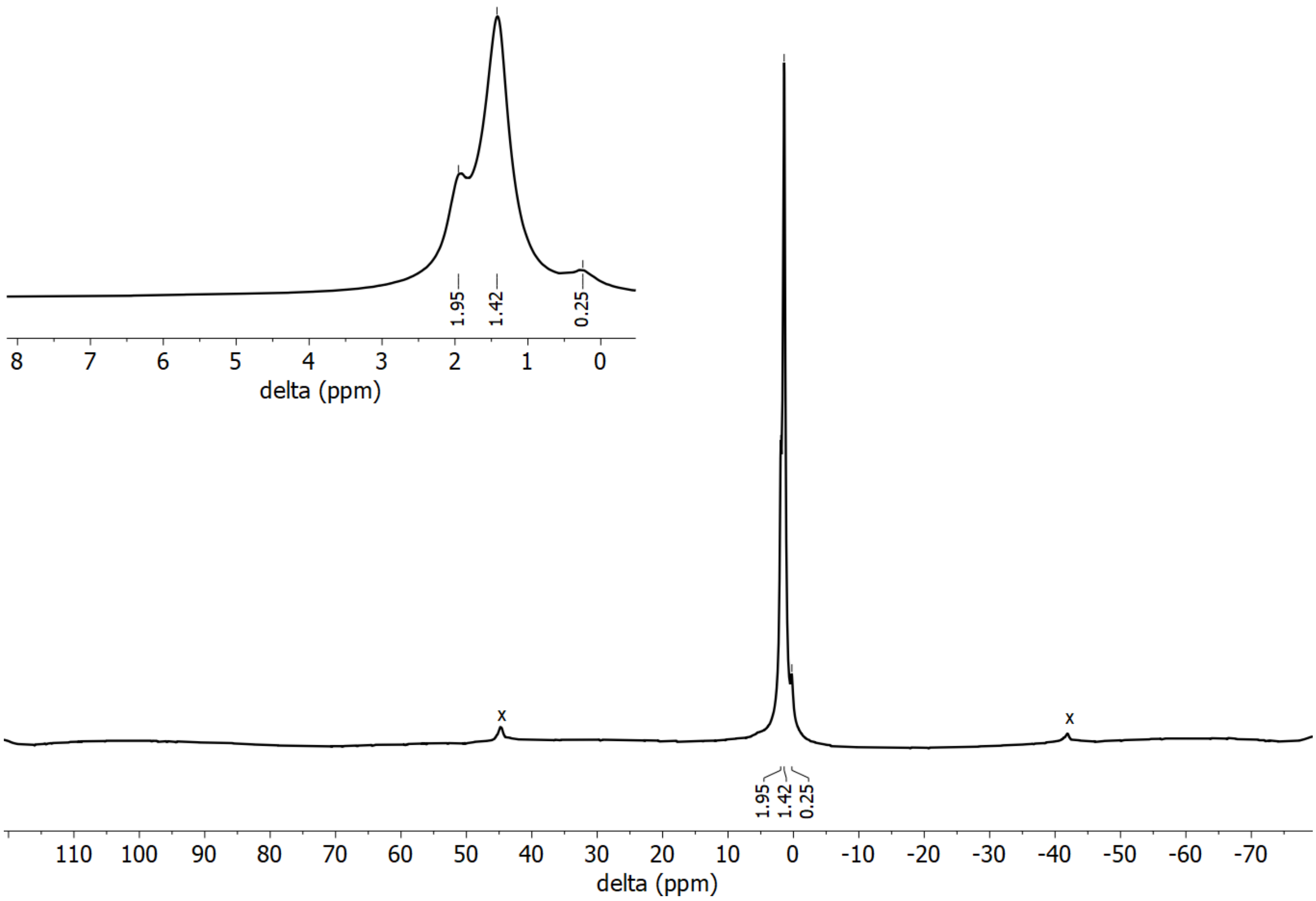

Figure S 28. Solid-state ^1H NMR spectrum (300 MHz) of $PtMe(PMe_3)_2/SiO_2{}^{700}$, pre-treated in a helium flow at 200 °C for 5 min (**A-He-200**); densely packed rotor (4 mm), magic-angle spinning 13 kHz, spinning side bands marked with x. The range between 0 and 8 ppm is enlarged in the upper left.

Supplementary material: Reactivity of A with hydrogen

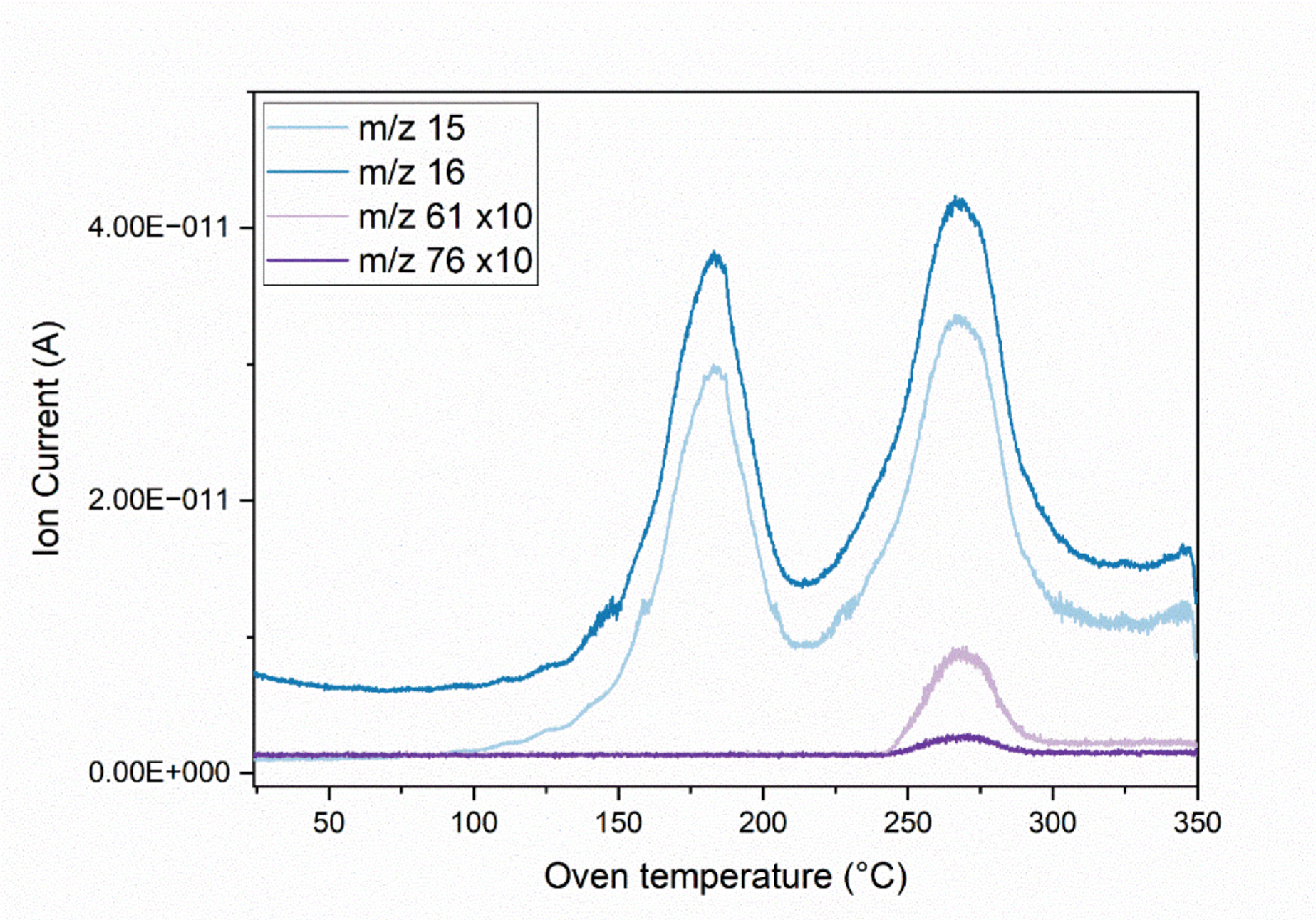

Figure S 29. Gas-analysis of out-stream by mass spectrometry: Heating of 100 mg $PtMe(PMe_3)_2/SiO_2^{700}$ placed in a U-shaped reactor with 5 $Kmin^{-1}$ in a H_2 (5 $mLmin^{-1}$) in helium flow (45 $mLmin^{-1}$); m/z 15, 16 (methyl, methane), m/z 61, 76 (multiplied by 10, fragments of PMe_3).

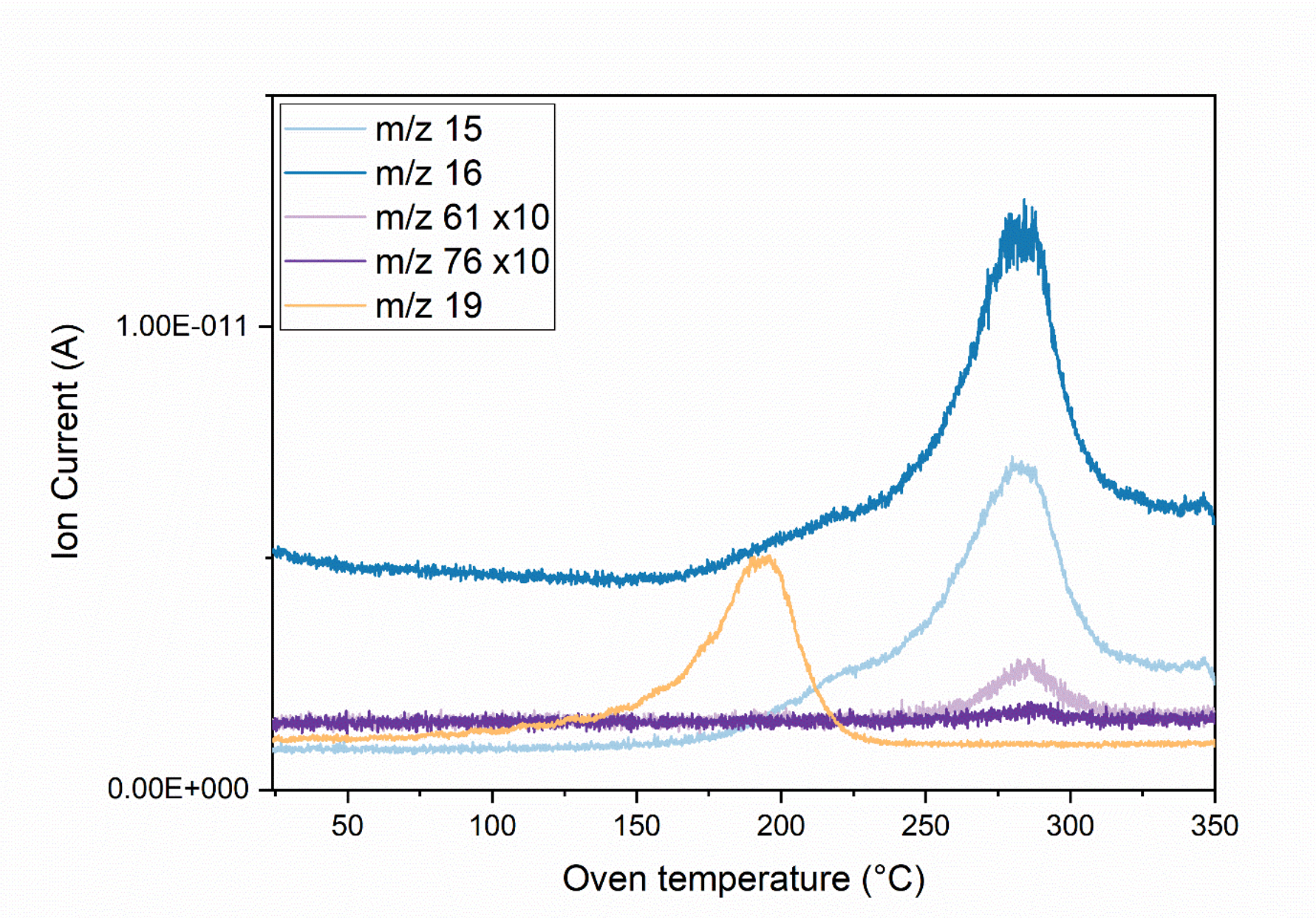

Figure S 30. Gas-analysis of out-stream by mass spectrometry: Heating of 50 mg $PtCD_3(PMe_3)_2/SiO_2^{700}$ placed in a U-shaped reactor with 5 $Kmin^{-1}$ in a H_2 (5 $mLmin^{-1}$) in helium flow (45 $mLmin^{-1}$); m/z 15, 16 (methyl, methane), m/z 19 (CD_3H), m/z 61, 76 (multiplied by 10, fragments of PMe_3).

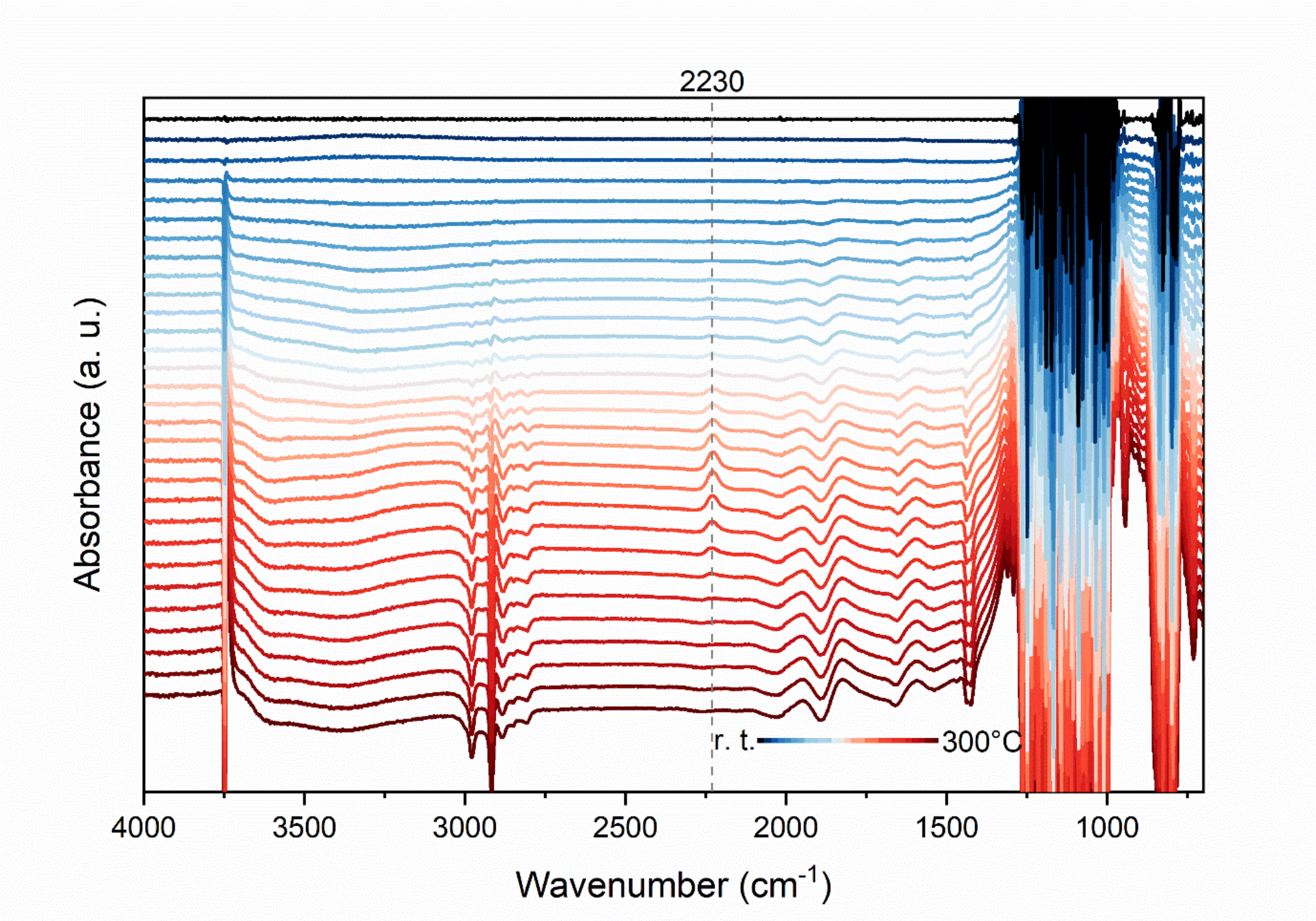

Figure S 31. Full temperature-dependent IR spectra of a self-supporting disc of **A** under a flow of H_2 (2 $mLmin^{-1}$, mixed with 18 $mLmin^{-1}$ helium) and heating with 5 $Kmin^{-1}$. A spectrum is collected every 10 K. The spectrum of the pellet of **A** before the treatment is subtracted.

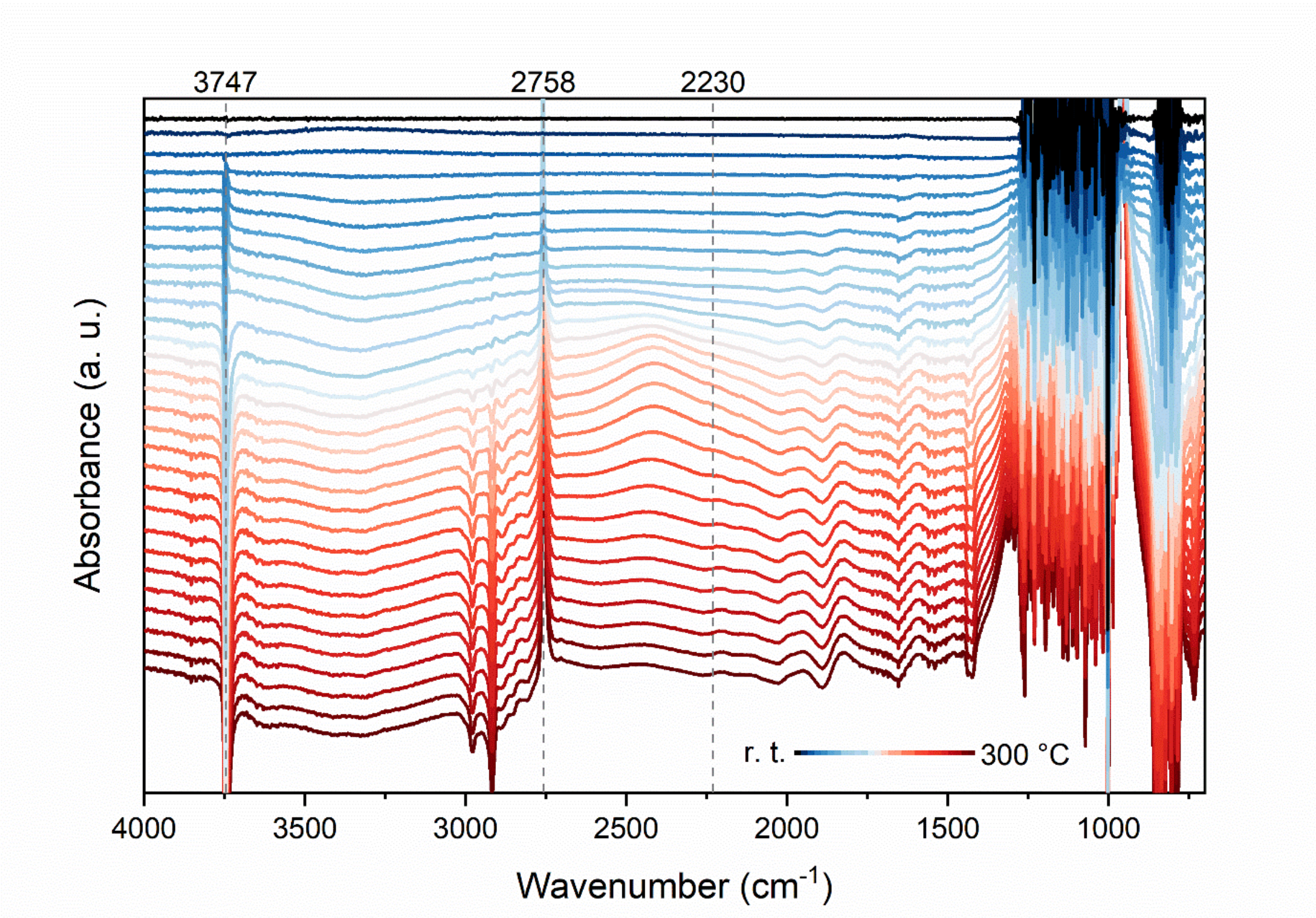

Figure S 32. Full temperature-dependent IR spectra of a self-supporting disc of **A** under a flow of D_2 (2 $mLmin^{-1}$, mixed with 18 $mLmin^{-1}$ helium) and heating with 5 $Kmin^{-1}$. A spectrum is collected every 10 K. The spectrum of the pellet of **A** before the treatment is subtracted.

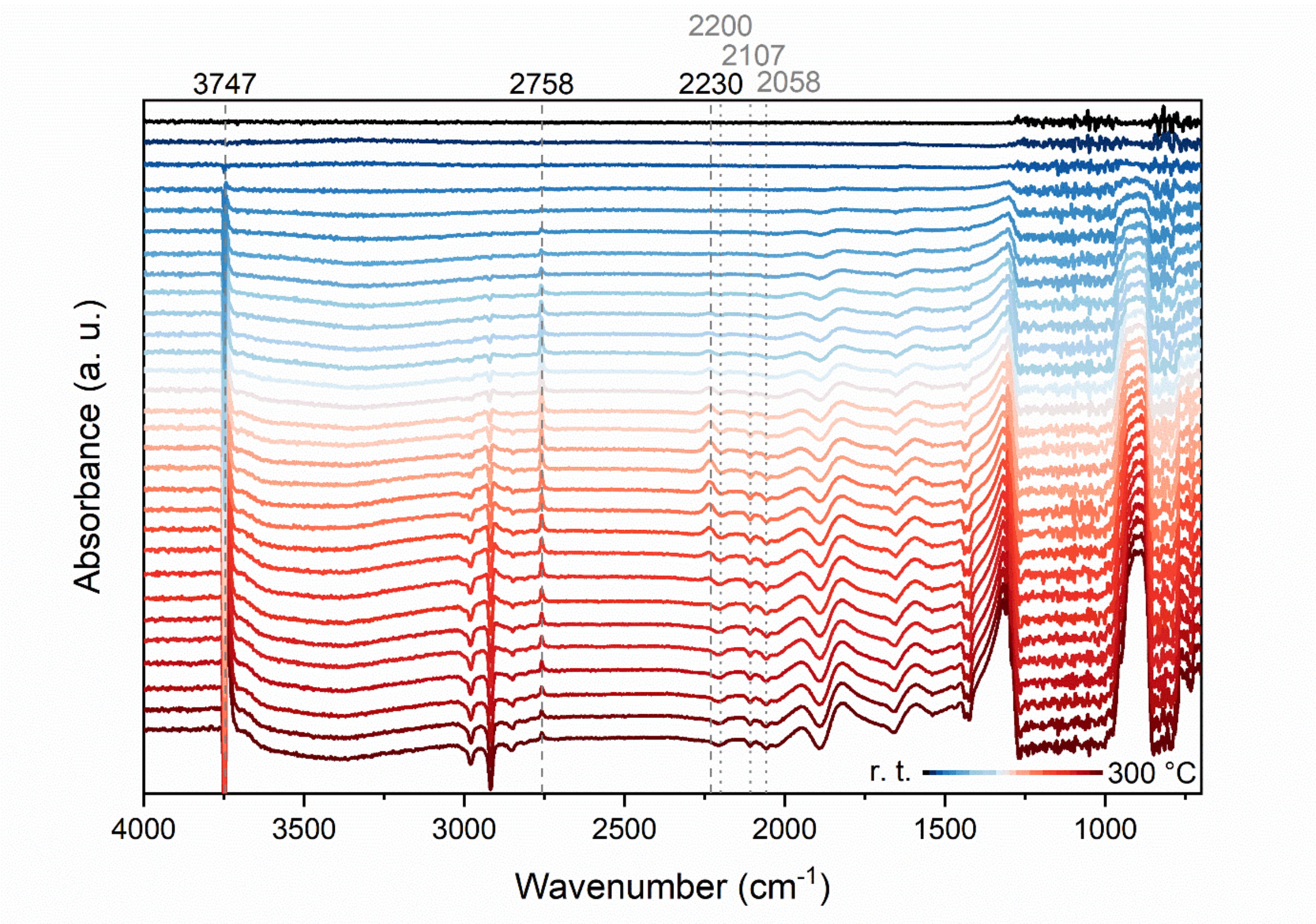

Figure S 33. Full temperature-dependent IR spectra of a self-supporting disc of **A-d$_3$** under a flow of H_2 (2 mLmin^{-1}, mixed with 18 mLmin^{-1} helium) and heating with 5 Kmin^{-1}. A spectrum is collected every 10 K. The spectrum of the pellet of **A-d$_3$** before the treatment is subtracted. C-D stretching bands are labeled in grey with dotted lines.

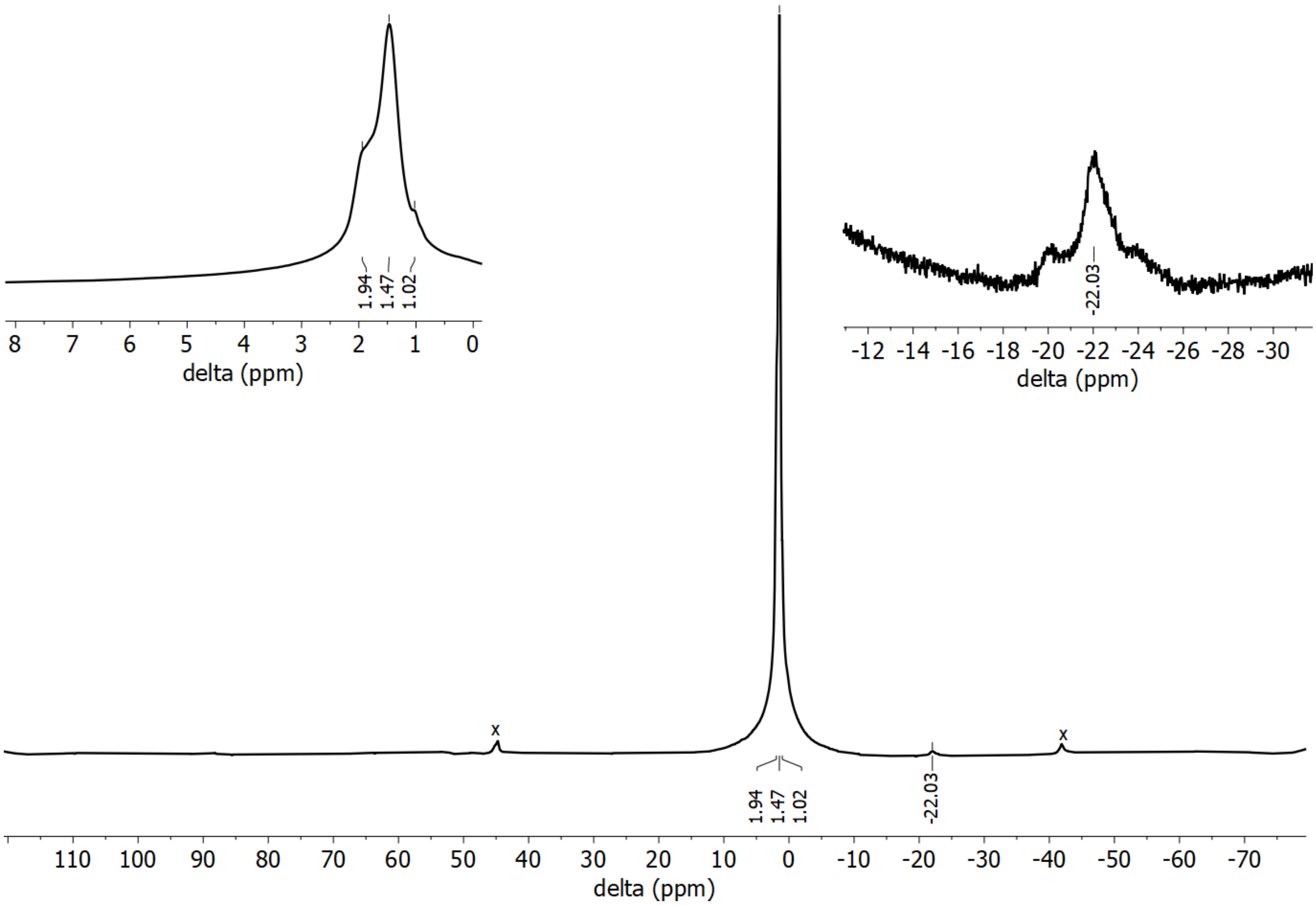

Figure S 34. Solid-state ^{1}H NMR spectrum (300 MHz) of $PtMe(PMe_3)_2/SiO_2^{700}$, pre-treated in a hydrogen (5 mLmin^{-1}) in helium (45 mLmin^{-1}) flow at 150 °C for 30 min (**A-H$_2$-150**); densely packed rotor (4 mm), magic-angle spinning 13 kHz, spinning side bands marked with x. The range between 0 and 8 ppm is enlarged on the upper left, the hydride signal is enlarged on the upper right.

EXAFS analysis of $PtMe(PMe_3)_2/SiO_2^{700}$, pre-treated in hydrogen

For the analysis of EXAFS data of $PtH(PMe_3)_2/SiO_2^{700}$ (**A-150-H$_2$**), the structural data of *cis*-[PtMe(OPh)(PMe$_3$)$_2$] (CCDC 1257257)[13] was used as the starting values for the fitting process. The complex describes the close surroundings of the Pt(II) scattering center in **A-150-H$_2$** partially (Table S 16). Inclusion of a Pt-H path was not considered, since hydrogen is a light element with a very low backscattering amplitude, resulting in a negligible contribution to the overall EXAFS signal. Additionally, platinum metal foil was not appropriate as a fit.

Table S 16. Coordination numbers and experimental interatomic distances for $PtH(PMe_3)_2/SiO_2^{700}$ by EXAFS analysis, compared to expected distances for the fit cis-[PtMe(OPh)(PMe$_3$)$_2$]

Scatter	Number of backscatterers (N_{BS})	$R_{expected}$ (Å)	$R_{experimental}$ (Å)	σ^2
Pt-P	2	2.36	2.26 ± 0.01	0.0013 ± 0.0028
Pt-O	1	2.15	1.96 ± 0.04	0.0061 ± 0.0039
S_0^2 = 0.7687; ΔE_0 = 1.22 ± 2.15 eV; X^2_{red} = 14.75; R^h = 0.0073; N(path) = 2; N(par) = 5; k-range: 3 – 10; R-range: 1 – 3.				

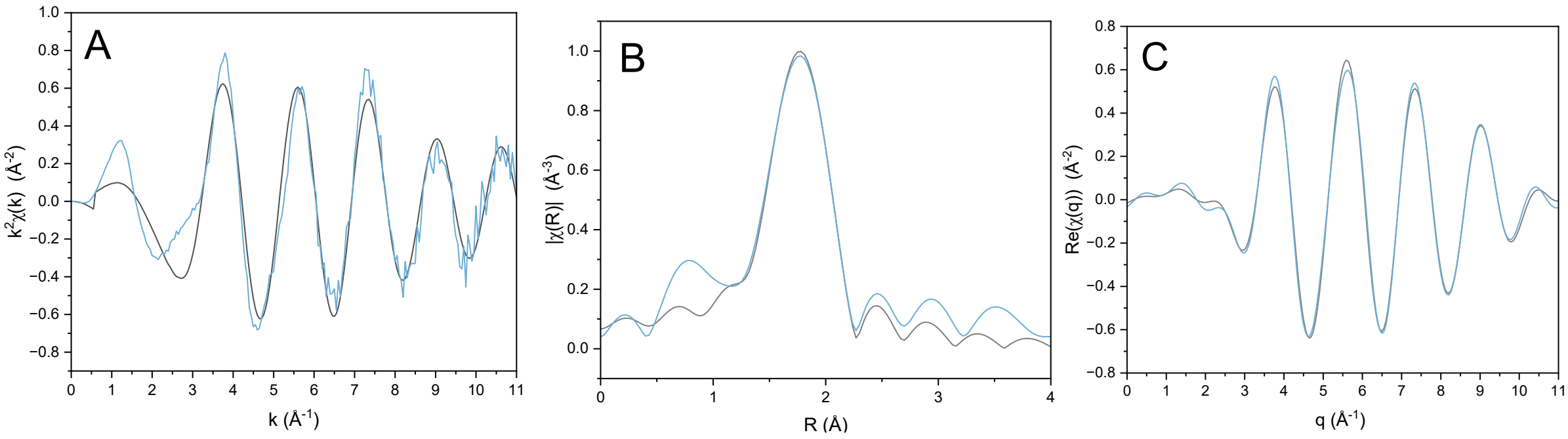

Figure S 35. Comparison of the Fourier-transformed EXAFS spectra for the surface complex $PtH(PMe_3)_2/SiO_2^{700}$ (light blue) and the best fit results based on the model complex $[PtMe(OPh)(PMe_3)_2]$ (grey). EXAFS spectra (A) Fourier-transformed EXAFS spectra (B) and the back transformation thereof (C).

Supplementary material: Reactivity of B and C with hydrogen

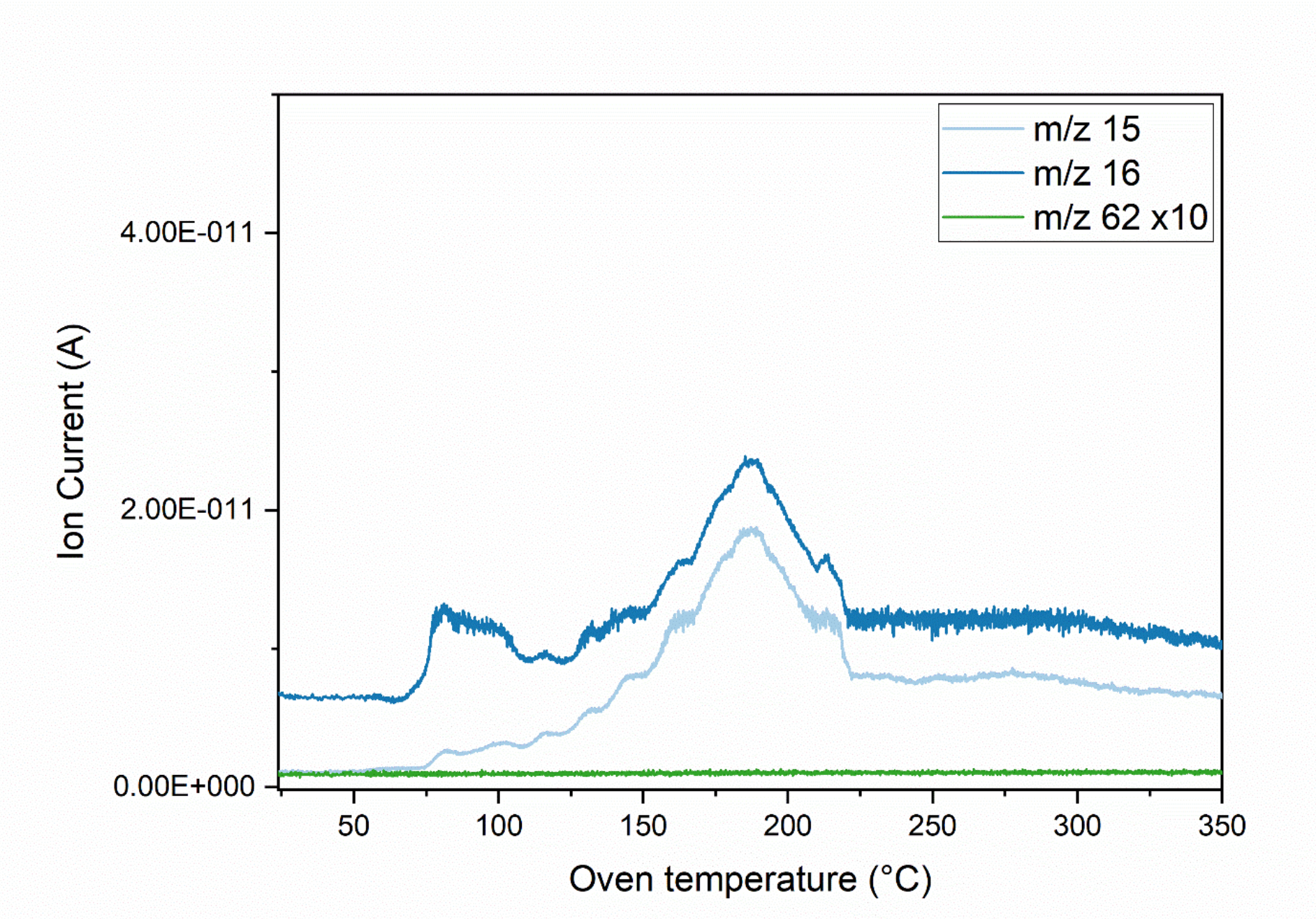

Figure S 36. Gas-analysis of out-stream by mass spectrometry: Heating of 100 mg PtMe(dmpe)/SiO_2^{700} placed in a U-shaped reactor with 5 $Kmin^{-1}$ in a H_2 (5 $mLmin^{-1}$) in helium flow (45 $mLmin^{-1}$); m/z 15, 16 (methyl, methane), m/z 62 (multiplied by 10, fragment of dmpe).

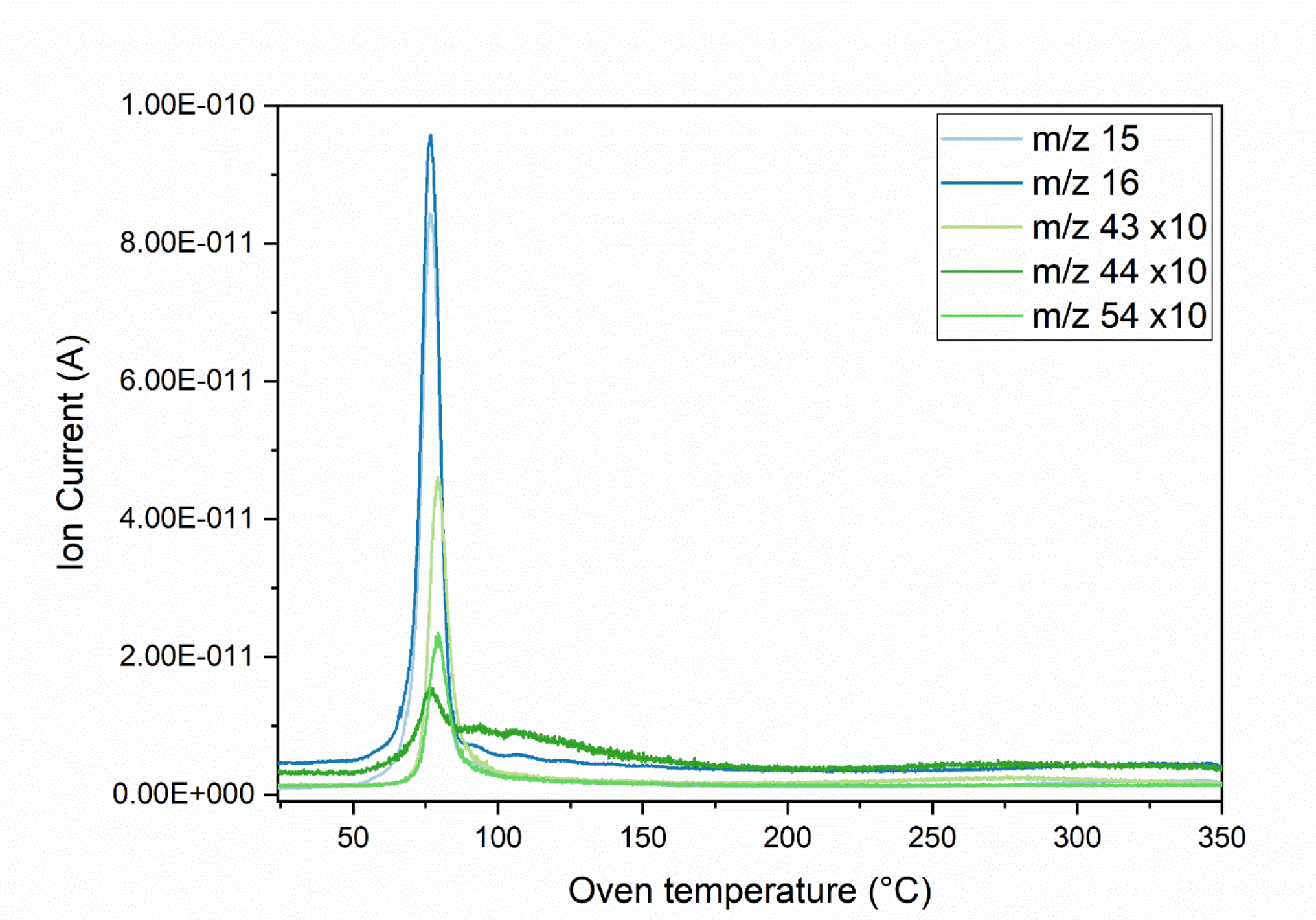

Figure S 37. Gas-analysis of out-stream by mass spectrometry: Heating of 100 mg PtMe(cod)/SiO_2^{700} placed in a U-shaped reactor with 5 $Kmin^{-1}$ in a H_2 (5 $mLmin^{-1}$) in helium flow (45 $mLmin^{-1}$); m/z 15, 16 (methyl, methane), m/z 43, 44, 54 (multiplied by 10, fragments of cod).

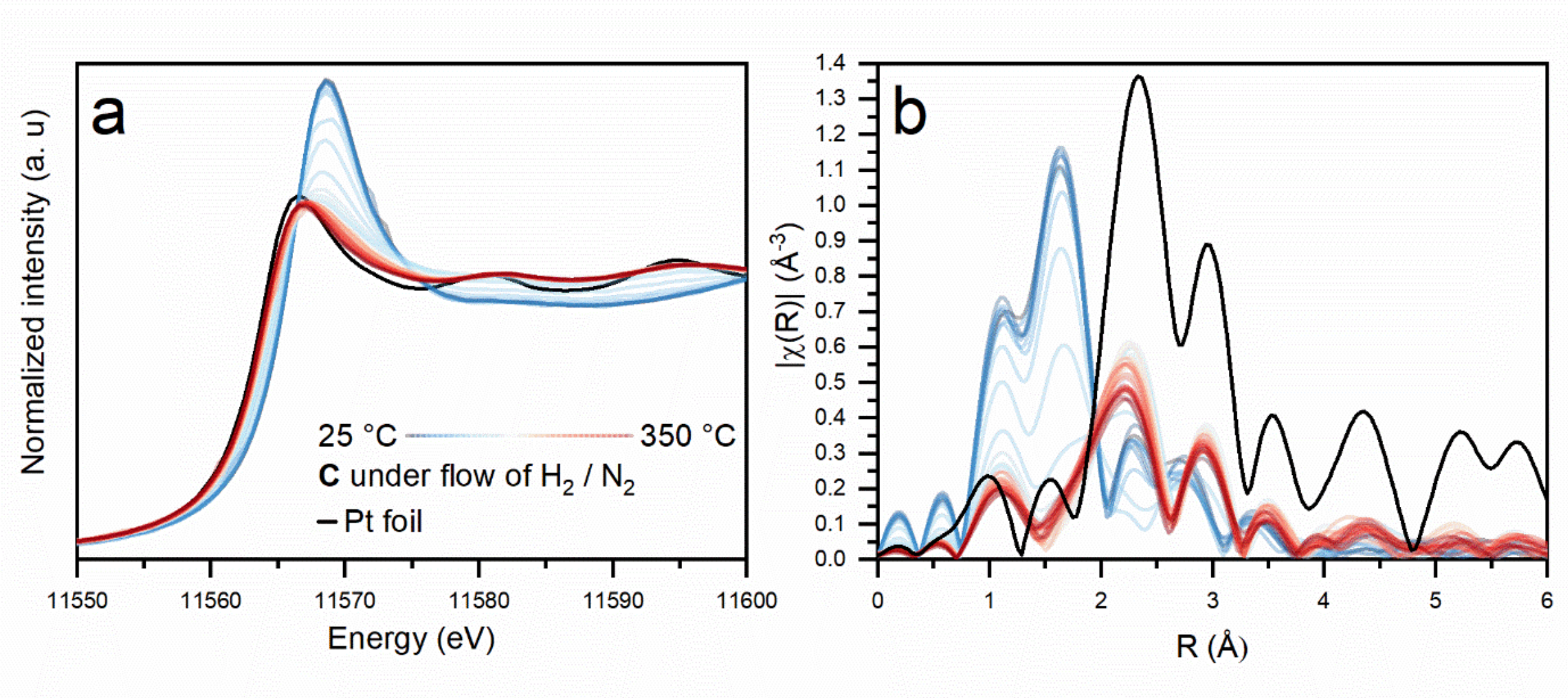

Figure S 38. a) *In situ* X-ray absorption spectra of **C** filled into a quartz glass capillary under a flow of H_2 (10% in nitrogen, 20 $mLmin^{-1}$) with 5 $Kmin^{-1}$ heating rate to 350 °C (color) in contrast to a spectrum of a platinum foil (black), and b) Fourier-transformations thereof.

After the reduction, the size of the platinum clusters (1.3 nm) was determined, based on ref.[21,22]

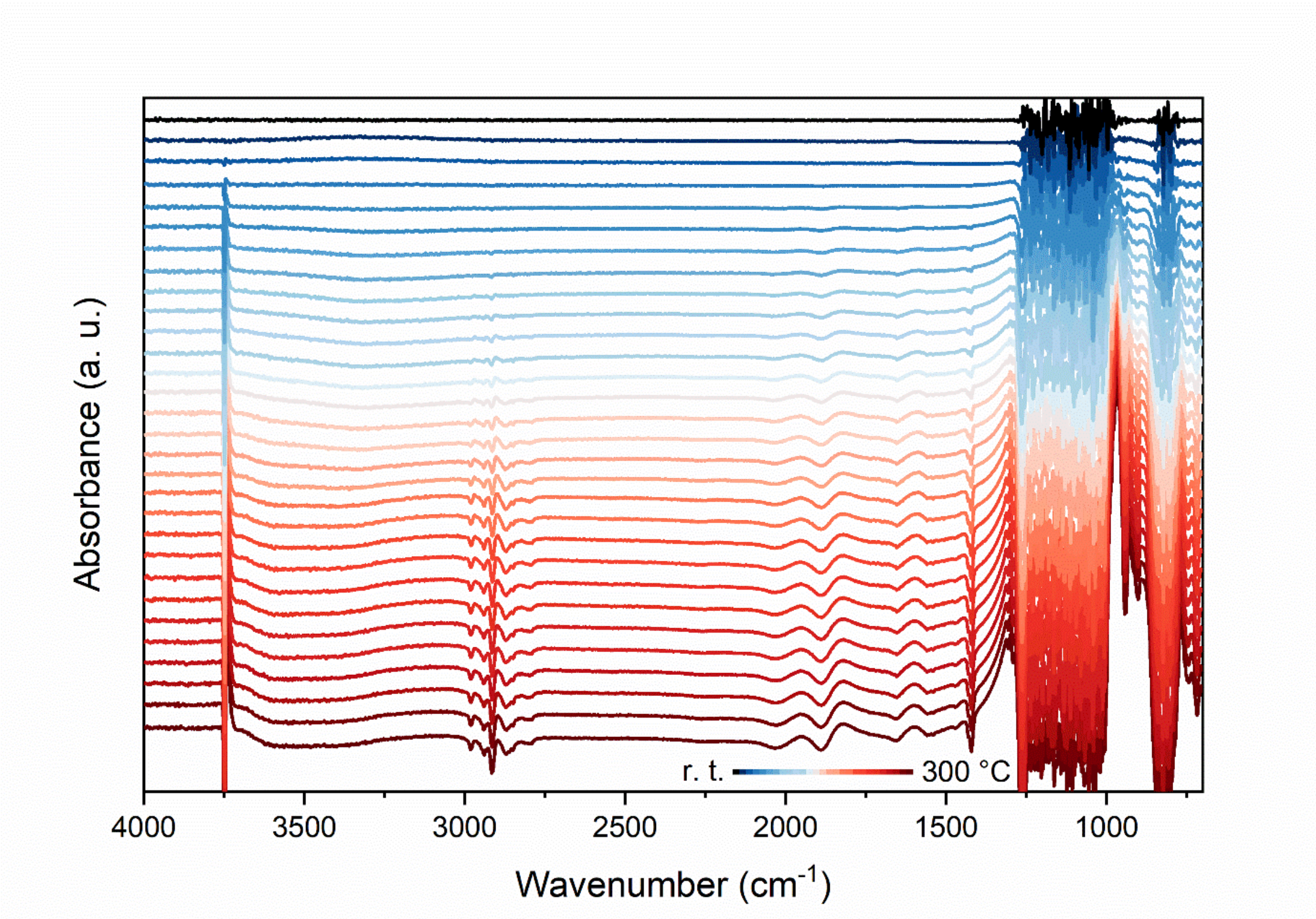

Figure S 39. Full temperature-dependent IR spectra of a self-supporting disc of **B** under a flow of H_2 (2 $mLmin^{-1}$, mixed with 18 $mLmin^{-1}$ helium) and heating with 5 $Kmin^{-1}$. A spectrum is collected every 10 K. The spectrum of the pellet of **B** before the treatment is subtracted.

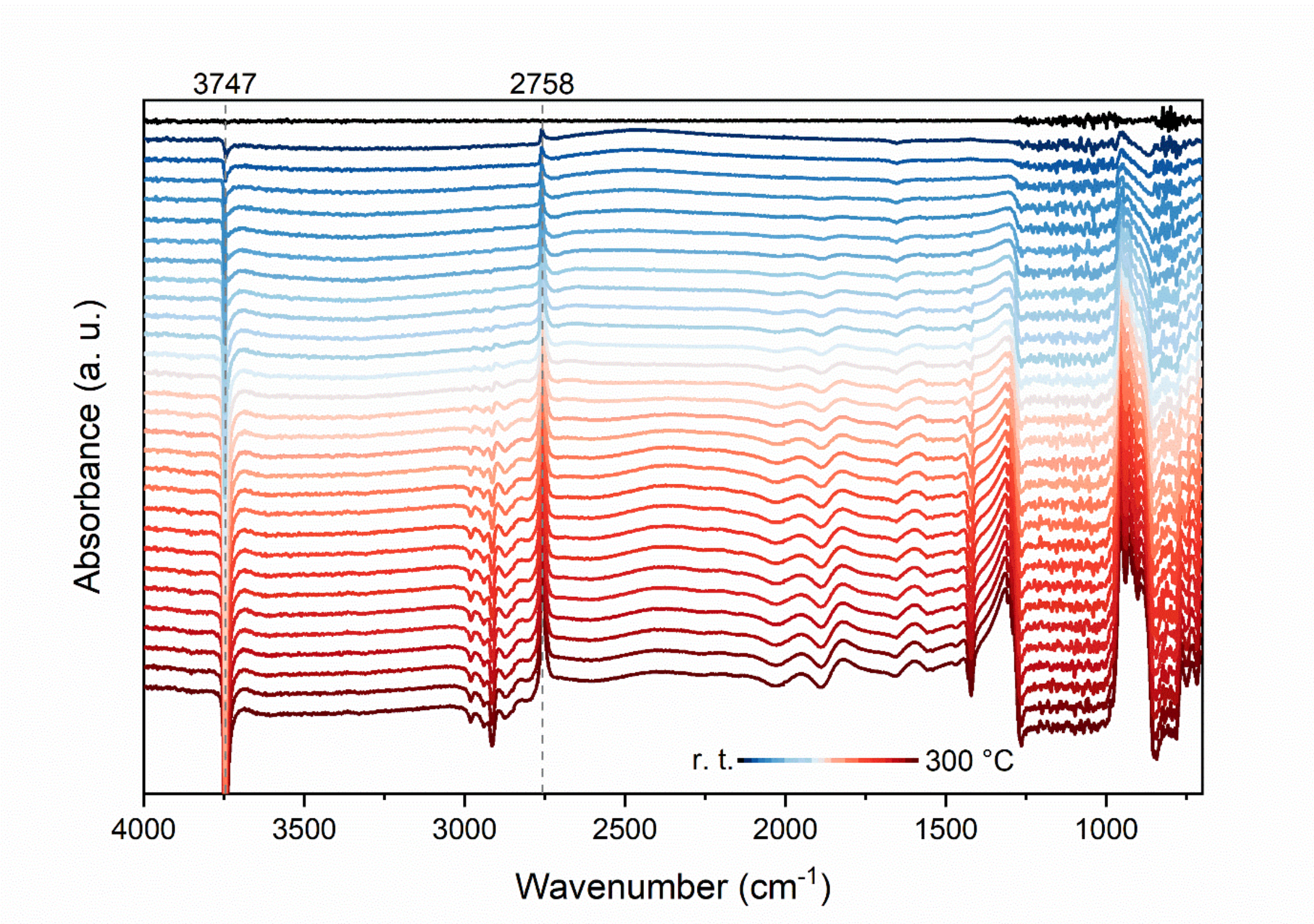

Figure S 40. Full, temperature-dependent IR spectra of a self-supporting disc of **B** under a flow of D_2 (2 mLmin^{-1}, mixed with 18 mLmin^{-1} helium) and heating with 5 Kmin^{-1}. A spectrum is collected every 10 K. The spectrum of the pellet of **B** before the treatment is subtracted.

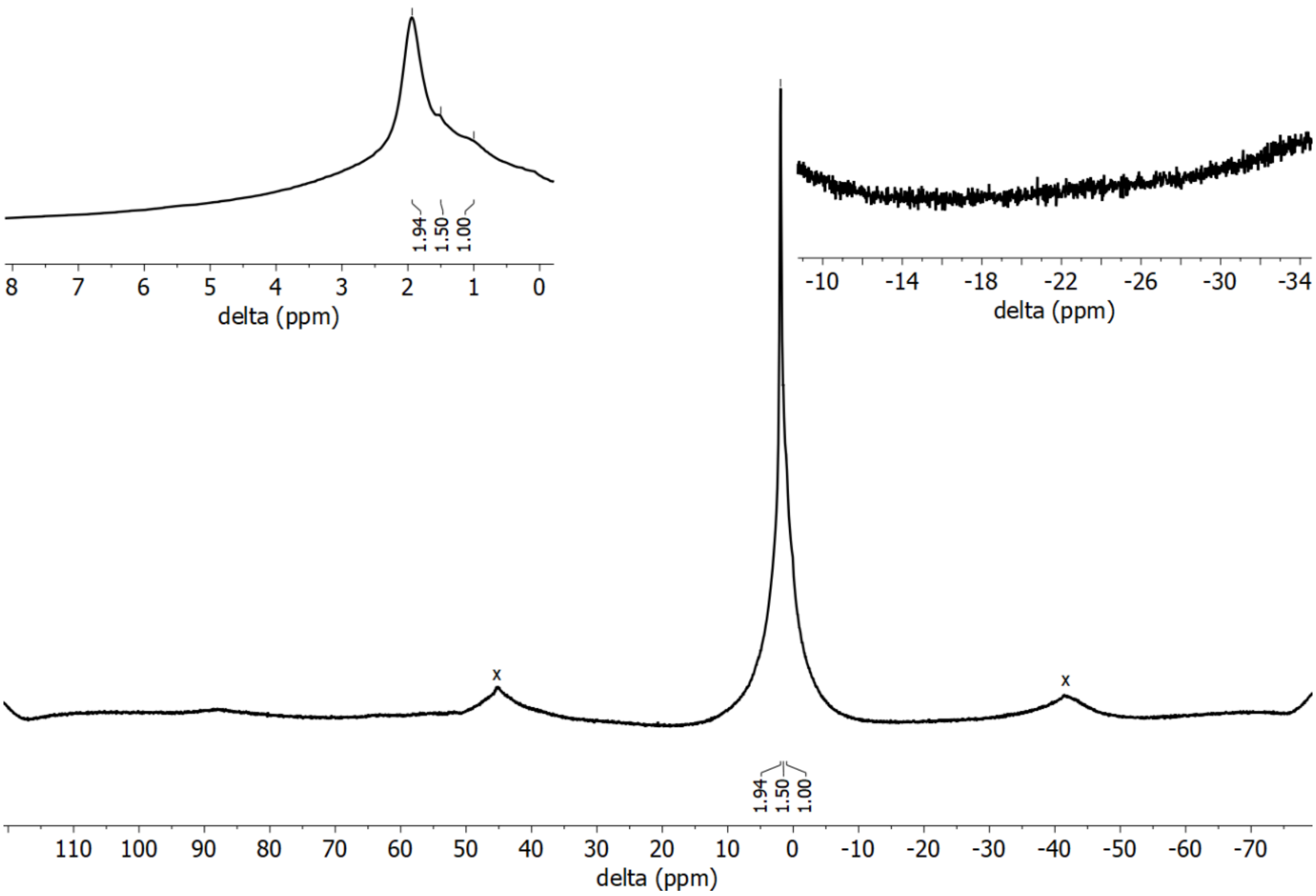

Figure S 41. Solid-state ^{1}H NMR spectrum (300 MHz) of PtMe(dmpe)/SiO_2^{700}, pre-treated in a hydrogen (5 mLmin^{-1}) in helium (45 mLmin^{-1}) flow at 150 °C for 30 min (**B-H$_2$-150**); densely packed rotor (4 mm), magic-angle spinning 13 kHz, spinning side bands marked with x. The range between 0 and 8 ppm is enlarged on the upper left, the region of hydride signals is enlarged on the upper right.

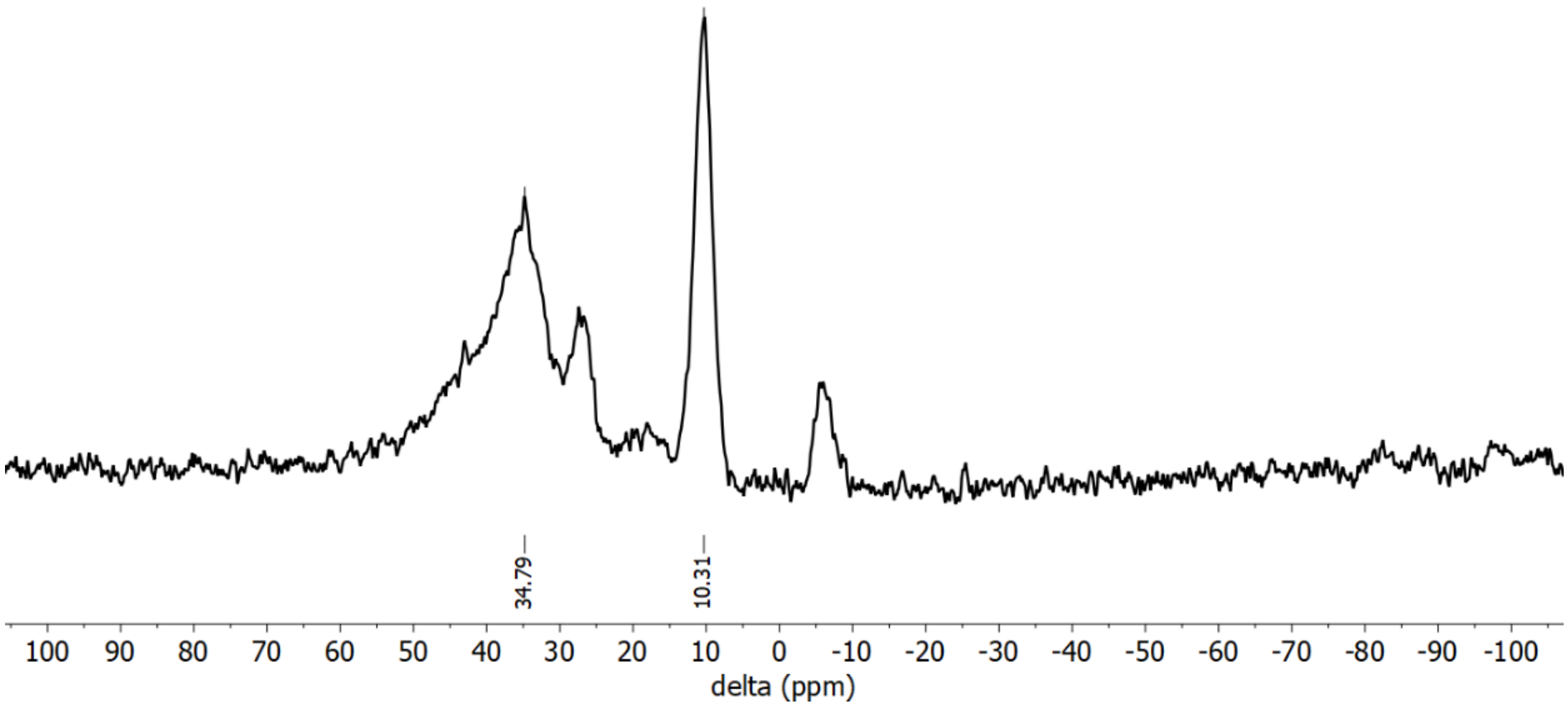

Figure S 42. Solid-state $^{31}P\{^{1}H\}$ NMR spectrum (121 MHz) of **B-H$_2$-150**; densely packed rotor (4 mm), magic-angle spinning 13 kHz.

Supplementary material: Test reaction: Isomerization of 1-hexene

The reaction was quantified by adding defined amounts of $FeCp_2$ to the reaction filtrate, which was analyzed by ^1H NMR spectroscopy. ^{31}P NMR spectroscopy of the filtrate did not give signals of leached compounds. The products 2- and 3-hexene give signals between 5.55-5.32 ppm (2- and 3-hexene), 1.62-1.58 ppm ((E)-2-hexene), 1.55-1.52 ppm ((Z)-2-hexene), and 0.97-0.92 ppm in the ^1H NMR spectrum in C_6D_6, which were used for determining conversion, selectivity, TON, and TOF given in Table 2 and Table S 18. The applied formulas are listed in Table S 17.

Table S 17. Formulas for determining conversions, selectivity, TON, and TOF of the test reaction

Quantity	Formula
Conversion (%)	$\frac{\text{Sum of the integrals of the products}}{\text{Sum of the integrals}} * 100$
$n_{2\text{- and 3-hexene}}$(mmol)	$\frac{\text{Integral}}{\text{Amount of H}} * n_{Ferrocene}(\text{mmol})$
Selectivity of 2 hexene (%)	$\frac{n_{2\text{-hexene}}(\text{mmol})}{n_{2\text{-and 3-hexene}}(\text{mmol})} * 100$
Turnover number (TON)	$\frac{n_{Products}(\text{mmol})}{n_{Pt}(\text{mmol})}$
Turnover frequency (TOF) (h^{-1})	$\frac{\text{TON}}{18}$

Table S 18. Summary of recycling experiments of the isomerization of 1-hexene with $PtMe(PMe_3)_2/SiO_2^{700}$, $PtMe(dmpe)/SiO_2^{700}$, and $PtMe(cod)/SiO_2^{700}$, pre-treated at 180 °C for 30 min in hydrogen (flow: 5 $mLmin^{-1}$, in helium flow: 45 $mLmin^{-1}$) (**A/B/C-H_2-180**)

	**Cycle 1** Conversion (%) / Selectivity 2-hexene (%)	**Cycle 2** Conversion (%) / Selectivity 2-hexene (%)	**Cycle 3** Conversion (%) / Selectivity 2-hexene (%)
**A-H_2-180**	8 / 92	7 / 88	3 / 89
**B-H_2-180**	49 / 87	36 / 93	26 / 92
**C-H_2-180**	1 / 86	1 / 81	1 / 78

As an example, an ^1H NMR spectrum of the reaction filtrate is shown in Figure S 43, which was obtained from using **B-H_2-180** as a catalyst in the first cycle. With this spectrum, the calculations for the values in Table 2 and Table S ***18*** can be comprehended.

- Conversion

$$\text{Conversion (\%)} = \frac{\text{Sum of the integrals of the products}}{\text{Sum of the integrals}} * 100 = \frac{(40.76+36.57+11.90+13.91)}{(2.01+40.76+3.82+49.88+36.57+11.90+18.24+13.91+32.46)} * 100$$

= 49.18%

- Molar amounts of 2- and 3-hexene

$$n_{2\text{- and 3-hexene}}(\text{mmol}) = \frac{\text{Integral}}{\text{Amount of H}} * n_{Ferrocene}(\text{mmol})$$

$$n_{2\text{-hexene}} = n_{2\text{-hexene (E)}} + n_{2\text{-hexene (Z)}} = \frac{36.57}{3} * 0.0134 \text{ mmol} + \frac{11.9}{3} * 0.0134 \text{ mmol} = 0.217 \text{ mmol}$$

$$n_{2\text{- and 3-hexene}} = n_{2\text{-hexene}} + \frac{13.91}{6} * 0.0134 \text{ mmol} = 0.248 \text{ mmol}$$

- Selectivity of 2-hexene

$$\text{Selectivity (\%)} = \frac{n_{2\text{-hexene}}(\text{mmol})}{n_{2\text{- and 3-hexene}}(\text{mmol})} * 100 = 87.45\%$$

- Turnover number (TON)

$$\text{TON} = \frac{n_{\text{Products}}(\text{mmol})}{n_{\text{Pt}}(\text{mmol})} = \frac{0.248 \text{ mmol}}{0.004 \text{ mmol}} = 62.09$$

- Turnover frequency (TOF)

$$\text{TOF } (h^{-1}) = \frac{\text{TON}}{18} = \frac{62.09}{18} = 3.4 \ h^{-1}$$

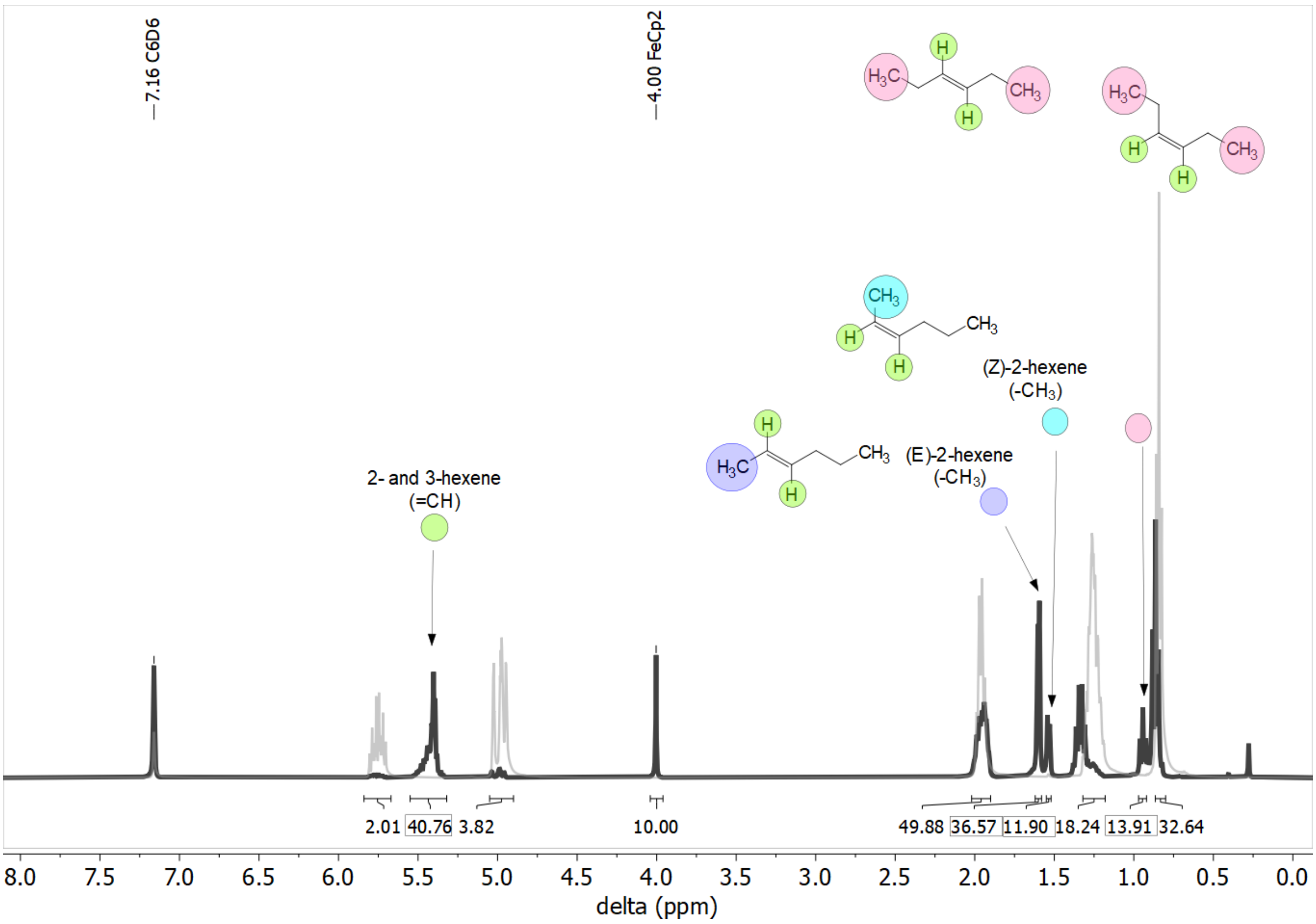

Figure S 43. ^{1}H NMR spectra (400 MHz, C_6D_6, r. t.) of 1-hexene (light grey) and the reaction filtrate of 1-hexene isomerization, using **B-H$_2$-180** as a catalyst in the first cycle (black). The given integrals refer to the reaction filtrate. The product signals (integrals with rectangles), which are included in the calculations, are marked with arrows with their assignments. For the quantification, 2.5 mg ferrocene were added to the reaction filtrate.

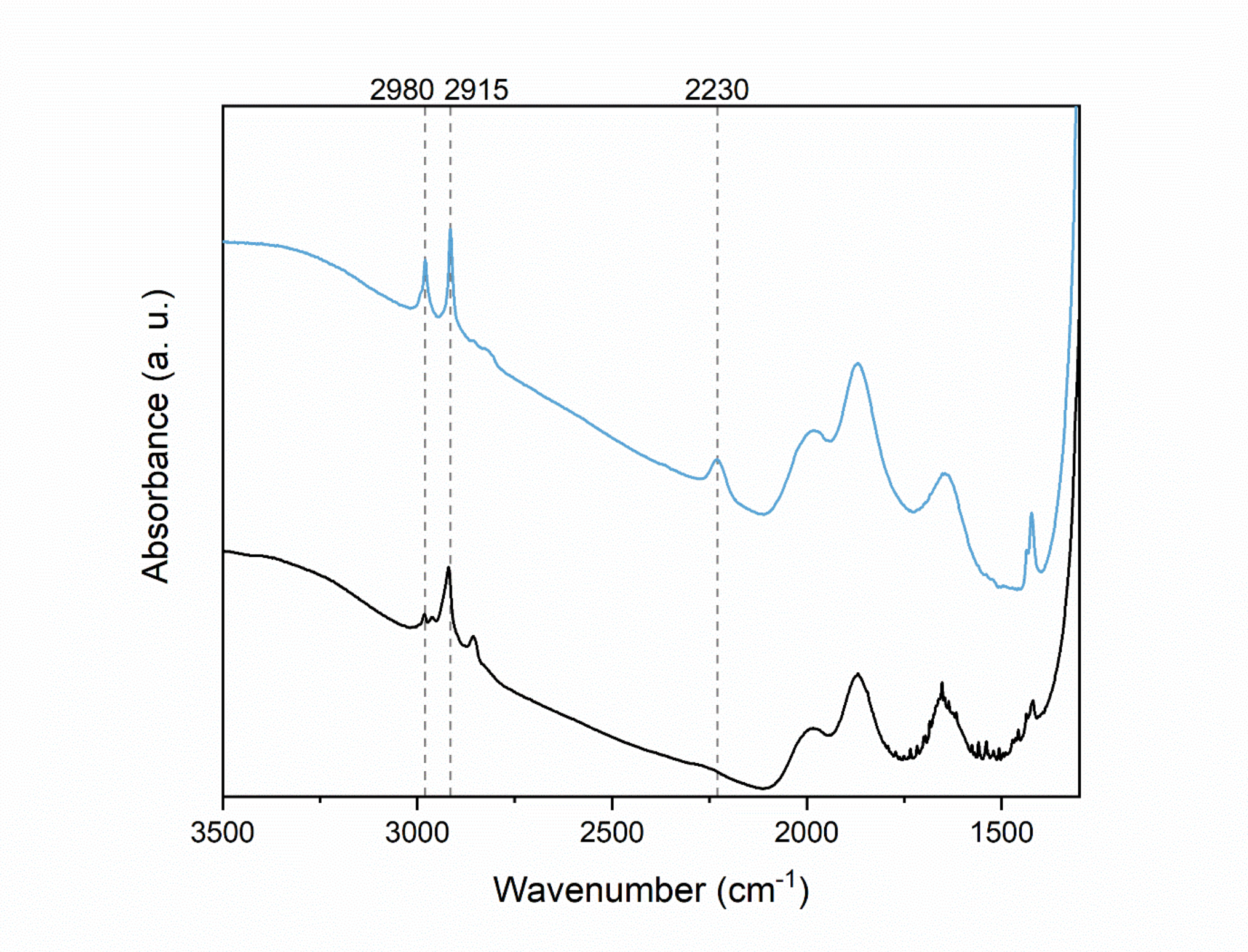

Figure S 44. FT-IR spectra of self-supporting discs of **A-H_2-150** (light blue) and **A-H_2-150** after one cycle of 1-hexene isomerization (black): Pt-H band at 2230 cm^{-1} is broad, suggesting insufficient reforming of the molecular species.

References

[1] Gmelin, “Handbuch der anorganischen Chemie, Platinum (D)” *8th Ed.* **1957**, no. 68, pp. 343–344.

[2] J. Chatt, B. L. Shaw, “Alkyls and Aryls of Transition Metals. Part II. Platinum(II) Derivatives.” *J. Chem. Soc.* **1959**, 4020–4033.

[3] R. Goikhman, M. Aizenberg, L. J. W. Shimon, D. Milstein, “Transition Metal-Catalyzed Silanone Generation” *J. Am. Chem. Soc.* **1996**, *118*, 10894–10895.

[4] D. A. Ruddy, J. Jarupatrakorn, R. M. Rioux, J. T. Miller, M. J. McMurdo, J. L. McBee, K. A. Tupper, T. Don Tilley, “Site-isolated Pt-SBA15 materials from tris(tert-butoxy)siloxy complexes of Pt(II) and Pt(IV)” *Chem. Mater.* **2008**, *20*, 6517–6527.

[5] S. L. Scott, P. Dufour, C. C. Santini, J.-M. Basset, “Surface Organometallic Chemistry on Oxides: Reaction of Trimethylphosphine with Bis(allyl)rhodium Grafted onto Silica” *Inorg. Chem.* **1996**, *35*, 869–875.

[6] M. J. Frisch, G. W. Trucks, H. B. Schlegel, G. E. Scuseria, M. A. Robb, J. R. Cheeseman, G. Scalmani, V. Barone, G. A. Petersson, H. Nakatsuji, X. Li, M. Caricato, A. V. Marenich, J. Bloino, B. G. Janesko, R. Gomperts, B. Mennucci, H. P. Hratchian, J. V. Ortiz, A. F. Izmaylov, J. L. Sonnenberg, D. Williams-Young, F. Ding, F. Lipparini, F. Egidi, J. Goings, B. Peng, A. Petrone, T. Henderson, D. Ranasinghe, V. G. Zakrzewski, J. Gao, N. Rega, G. Zheng, W. Liang, M. Hada, M. Ehara, K. Toyota, R. Fukuda, J. Hasegawa, M. Ishida, T. Nakajima, Y. Honda, O. Kitao, H. Nakai, T. Vreven, K. Throssell, J. A. Montgomery Jr., J. E. Peralta, F. Ogliaro, M. J. Bearpark, J. J. Heyd, E. N. Brothers, K. N. Kudin, V. N. Staroverov, T. A. Keith, R. Kobayashi, J. Normand, K. Raghavachari, A. P. Rendell, J. C. Burant, S. S. Iyengar, J. Tomasi, M. Cossi, J. M. Millam, M. Klene, C. Adamo, R. Cammi, J. W. Ochterski, R. L. Martin, K. Morokuma, O. Farkas, J. B. Foresman, D. J. Fox, Gaussian 16 Revision B.01, Gaussian Inc., Wallingford CT, **2016**.

[7] A. D. Becke, “Density-functional Thermochemistry. III. The Role of Exact Exchange” *J. Chem. Phys.* **1993**, *98*, 5648–5652.

[8] C. Lee, W. Yang, R. G. Parr, “ Accurate and Simple Analytic Representation of the Electron Gas Correlation Energy” *Phys. Rev. B* **1988**, *37*, 785–789.

[9] P. J. Hay, W. R. Wadt, “*Ab initio* effective core potentials for molecular calculations. Potentials for K to Au including the outermost core orbitals” *J. Chem. Phys.* **1985**, *82*, 299–310.

[10] M. J. Frisch, J. A. Pople, J. S. Binkley, “Self-consistent molecular orbital methods 25. Supplementary functions for Gaussian basis sets” *J. Chem. Phys.* **1984**, *80*, 3265–3269.

[11] “NIST Computational Chemistry Comparison and Benchmark Database” http://cccbdb.nist.gov/ DOI:10.18434/T47C7Z

[12] C. Copéret, M. Chabanas, R. Petroff Saint-Arroman, J.-M. Basset, “Homogeneous and Heterogeneous Catalysis: Bridging the Gap through Surface Organometallic Chemistry” *Angew. Chem. Int. Ed.* **2003**, *42*, 156–181.

[13] K. Osakada, Y.-J. Kim, A. Yamamoto, “Preparation and Properties of Methylplatinum Fluoroalkoxide and Phenoxide Complexes, PtMe(OR)(PMe3)2 and PtMe(OR)(HOR)(PMe3)2 (R = CH(CF3)2, C6H5)” *J. Organomet. Chem.* **1990**, *382*, 303–317.

[14] A. Klein, K. W. Klinkhammer, T. Scheiring, “Cyclooctadienemethylplatinum complexes: Synthesis, reactivity, molecular structure and spectroscopic properties of the organometallic hydroxoplatinum(II) complex [(COD)PtMe(OH)]” *J. Organomet. Chem.* **1999**, *592*, 128–135.

[15] R. Romeo, G. Alibrandi, “Structure-Reactivity Correlations for the Dissociative Uncatalyzed Isomerization of Monoalkylbis(phosphine)platinum(II) Solvento Complexes” *Inorg. Chem.* **1997**, *36*, 4822–4830.

[16] Y. Kayaki, H. Tsukamoto, M. Kaneko, I. Shimizu, A. Yamamoto, M. Tachikawa, T. Nakajima, "Experimental and theoretical studies on the course of CO insertion into Pt-C and Pd-C bonds in neutral and cationic complexes, [MR(Cl){P(CH3)3}2] and [MR{P(CH3)3}2(s)]+BF 4- (M=Pt, Pd, R=CH3, C6H5, s=coordinated solvent)" *J. Organomet. Chem.* **2001**, *622*, 199–209.

[17] C. J. Wilson, M. Green, R. J. Mawby, "Preparation and Reactions of Alkyl Carbonyl Complexes of Platinum(II)" *J. Chem. Soc., Dalton Trans.* **1974**, 421–423.

[18] D. Ramprasad, H. J. Yue, J. A. Marsella, "Synthesis and Characterization of trans-(PMe3)2PtHCl and an Investigation of its Role in Olefin Hydration Catalysis" *Inorg. Chem.* **1988**, *27*, 3151–3155.

[19] G. Booth, J. Chatt, P. Chini, "Platinum Carbonyls Substituted by Tertiary Phosphines" *Chem. Commun. (London)* **1965**, 639–640.

[20] J. Chatt, P. Chini, "Tri- and Tetra-nuclear Cluster Compounds of Platinum(0), which contain Tertiary Phosphines and Carbon Monoxide" *J. Chem. Soc. A* **1970**, 1538–1542.

[21] S. Calvin, M. M. Miller, R. Goswami, S. F. Cheng, S. P. Mulvaney, L. J. Whitman, V. G. Harris, "Determination of crystallite size in a magnetic nanocomposite using extended x-ray absorption fine structure" *J. Appl. Phys.* **2003**, *94*, 778–783.

[22] A. Jentys, "Estimation of mean size and shape of small metal particles by EXAFS" *Phys. Chem. Chem. Phys.* **1999**, *1*, 4059–4063.
